# Development of a deep learning model for predicting recurrence of hepatocellular carcinoma after liver transplantation

**DOI:** 10.3389/fmed.2024.1373005

**Published:** 2024-06-11

**Authors:** Seung Hyoung Ko, Jie Cao, Yong-kang Yang, Zhi-feng Xi, Hyun Wook Han, Meng Sha, Qiang Xia

**Affiliations:** ^1^Department of Liver Surgery, Renji Hospital, School of Medicine, Shanghai Jiao Tong University, Shanghai, China; ^2^Department of Medicine, CHA University, Seongnam-si, Republic of Korea

**Keywords:** deep learning, survival prediction, hepatocellular carcinoma, liver transplantation, tabular data

## Abstract

**Background:**

Liver transplantation (LT) is one of the main curative treatments for hepatocellular carcinoma (HCC). Milan criteria has long been applied to candidate LT patients with HCC. However, the application of Milan criteria failed to precisely predict patients at risk of recurrence. As a result, we aimed to establish and validate a deep learning model comparing with Milan criteria and better guide post-LT treatment.

**Methods:**

A total of 356 HCC patients who received LT with complete follow-up data were evaluated. The entire cohort was randomly divided into training set (*n* = 286) and validation set (*n* = 70). Multi-layer-perceptron model provided by pycox library was first used to construct the recurrence prediction model. Then tabular neural network (TabNet) that combines elements of deep learning and tabular data processing techniques was utilized to compare with Milan criteria and verify the performance of the model we proposed.

**Results:**

Patients with larger tumor size over 7 cm, poorer differentiation of tumor grade and multiple tumor numbers were first classified as high risk of recurrence. We trained a classification model with TabNet and our proposed model performed better than the Milan criteria in terms of accuracy (0.95 vs. 0.86, *p* < 0.05). In addition, our model showed better performance results with improved AUC, NRI and hazard ratio, proving the robustness of the model.

**Conclusion:**

A prognostic model had been proposed based on the use of TabNet on various parameters from HCC patients. The model performed well in post-LT recurrence prediction and the identification of high-risk subgroups.

## 1 Introduction

Hepatocellular carcinoma (HCC) is the most common primary liver cancer, ranks the third leading cause of cancer-associated mortality worldwide (1, 2). Liver transplantation (LT) is one of the main curative treatments for HCC, eliminating the tumor and the underlying liver disease simultaneously (3, 4). Milan criteria, first introduced by Mazzaferro in 1996, is the main policy to select patients with HCC for LT globally (5). Patients within Milan criteria are reported to achieve 5-year survival of approximately 80%. However, the strict selection criteria and another 20% of tumor recurrence after LT remains a major concern and impedes the curative chance for HCC patients (6).

Therefore, constructing a model better selecting patients and predicting tumor recurrence is a major issue in LT treatment. Several risk factors have been identified contributing to HCC recurrence after LT, including tumor morphological characteristics and pathological features (7). AFP, a commonly used serum marker, is highly specific for predicting HCC recurrence (8). Presence of microvascular invasion is also considered as an independent factor in recurrence prediction (9). Other crucial indicators consist of tumor differentiation, pre-transplant treatment, wait time, etc. Based on the above predictors, several models beyond Milan criteria have been constructed in predicting HCC recurrence (10–12). For example, Metroticket Model was developed based on tumor size, tumor number and AFP level to predict HCC recurrence using a Cox-PH regression analysis (13).

In the past few years, artificially intelligence (AI) has been increasingly used for the recurrence of HCC after liver resection. For instance, Ji et al. identified a three-feature signature using the machine-learning framework that demonstrated favorable prediction of HCC recurrence with C-index of 0.633–0.699 (14). Liu et al. proposed a prognostic classifier based on deep learning to identify high-risk recurrence of hepatectomy patients who may benefit from intensive management (15). With the advantage of integrating various risk factors, the advanced AI algorithms and techniques are also urgently needed in developing recurrence prediction models for LT patients with HCC.

Recently, the Google Cloud AI research team invented TabNet as a novel deep learning model for tabular data and it has been shown to be more effective in variety of tasks (16). In the present study, we proposed a model by utilizing TabNet for predicting tumor recurrence after LT. The new model was compared with the Milan criteria and aimed to better select patients for LT.

## 2 Methods

### 2.1 Data collection and follow-up

Patients who underwent liver transplantation in Renji Hospital, School of Medicine, Shanghai Jiao Tong University from January 2015 to December 2018 were retrospectively reviewed. Patients were excluded according to the criteria as follows: (1) pathological diagnosis of intrahepatic cholangiocarcinoma (ICC), combined HCC-ICC or other malignancies; (2) perioperative death due to infection, bleeding, organ failure, etc.; (3) incomplete medical records; and (4) loss of follow-up within 90 days after LT. This study was approved by the institutional ethics committee of Renji Hospital, School of Medicine, Shanghai Jiao Tong University.

Preoperative demographic data and serological examinations including age, gender, hepatitis virus infection, liver function, AFP level, and pre-transplant therapy (liver resection, TACE and RFA) were collected. Data of cirrhosis, tumor number, maximal diameter, satellite lesions, pathological grade, microvascular invasion and portal vein tumor thrombus were collected based on postoperative pathology.

All patients were followed up monthly during the 1^st^ year and every 3 months thereafter. The clinical testing included liver function, serum AFP level as well as abdominal ultrasound. To allow early detection of recurrence, CT or MRI scan of the chest and abdomen were performed once every 6 months. When tumor recurrence was suspected, PET-CT was conducted. Adjuvant therapy including transarterial chemoembolization (TACE), radiofrequency ablation, sorafenib or lenvatinib were permitted once tumor recurrence was confirmed. The main endpoint of this study was recurrence of tumor and death of patients. Data of overall (OS) and recurrence-free survival (RFS) were collected for all included patients.

### 2.2 Extraction of patient features for risk groups

**Figure 1:**
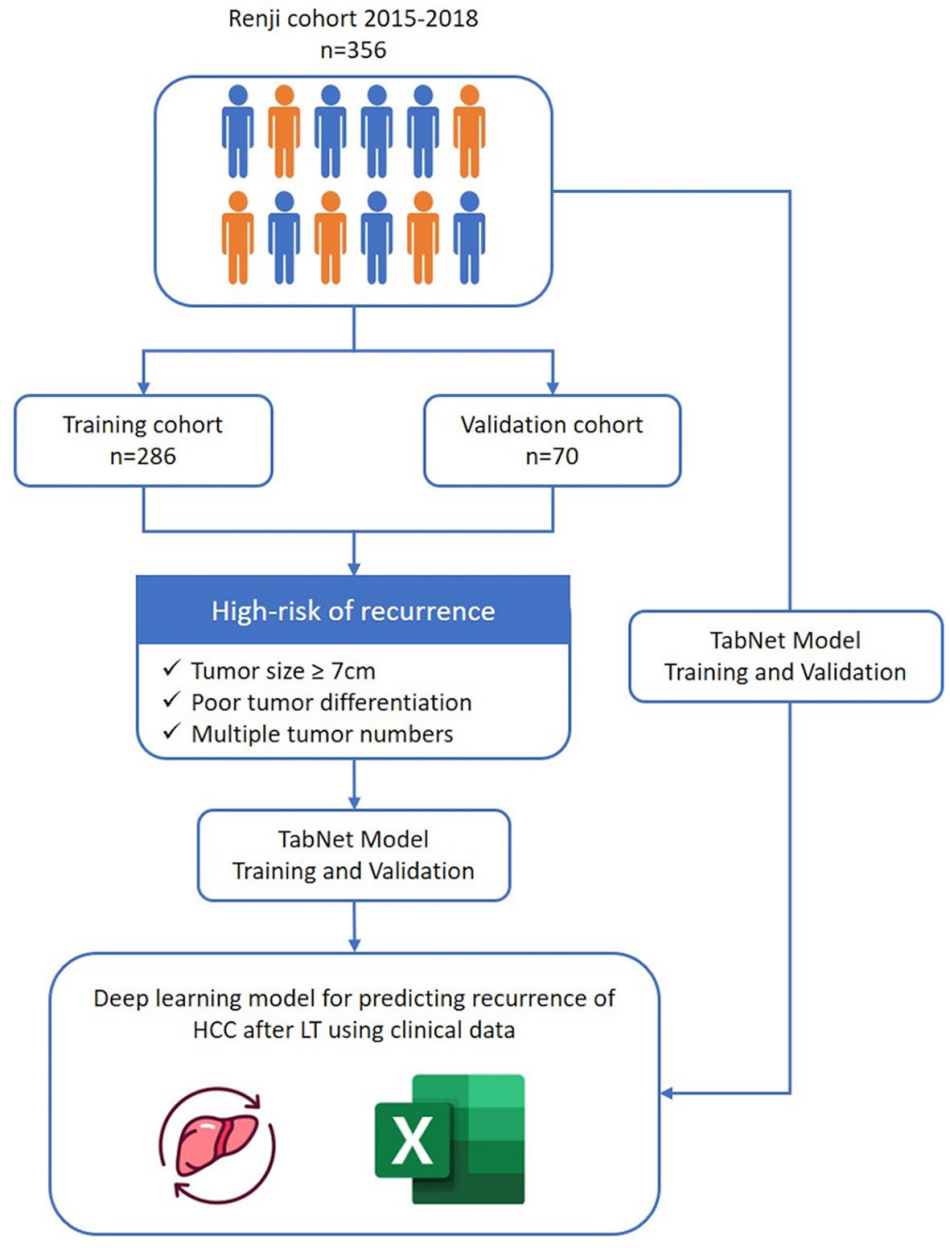
Total process of experiments (from extracting features to survival analysis).

The survival model calculated the probability for a period from a minimum of 3 months to a maximum of 95 months for each patient. We set the final survival period after liver transplantation at 5 years and used a survival analysis model with pycox to extract features of the low-risk group of patients whose 5-year survival time exceeds 0.7 and the high-risk group of patients whose survival probability lower than 0.2. Characteristics of high-risk patients were extracted. The model is a 48-node MLP (Multi-Layer Perceptron) and uses two hidden layers, ReLU activation function, a batch norm and dropout. Dropout rate is 0.1, optimized with Adam, and batch size is 256. Tumor size, pathological grade, and tumor number were considered as extracted features for high-risk and low-risk patient groups. The average value of the three variables in each patient group was calculated and set as the threshold value for classifying high-risk and low-risk patients (Figure 1).

### 2.3 TabNet

**Figure 2:**
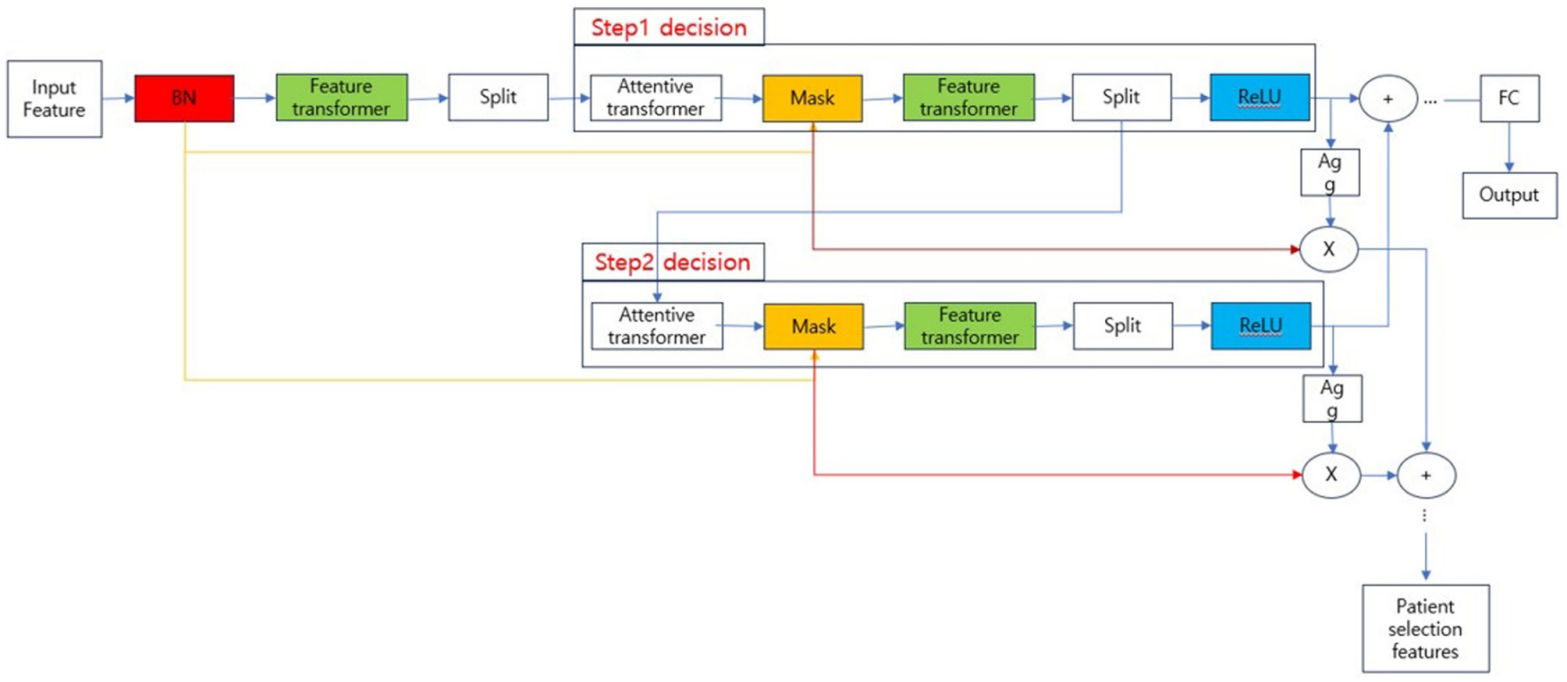
Tabnet architecture.

TabNet, the model used to train the classification model in our experiments, is known as a type of neural network that processes tabular data. The overall structure of TabNet is shown in Figure 2.

**Figure 3:**
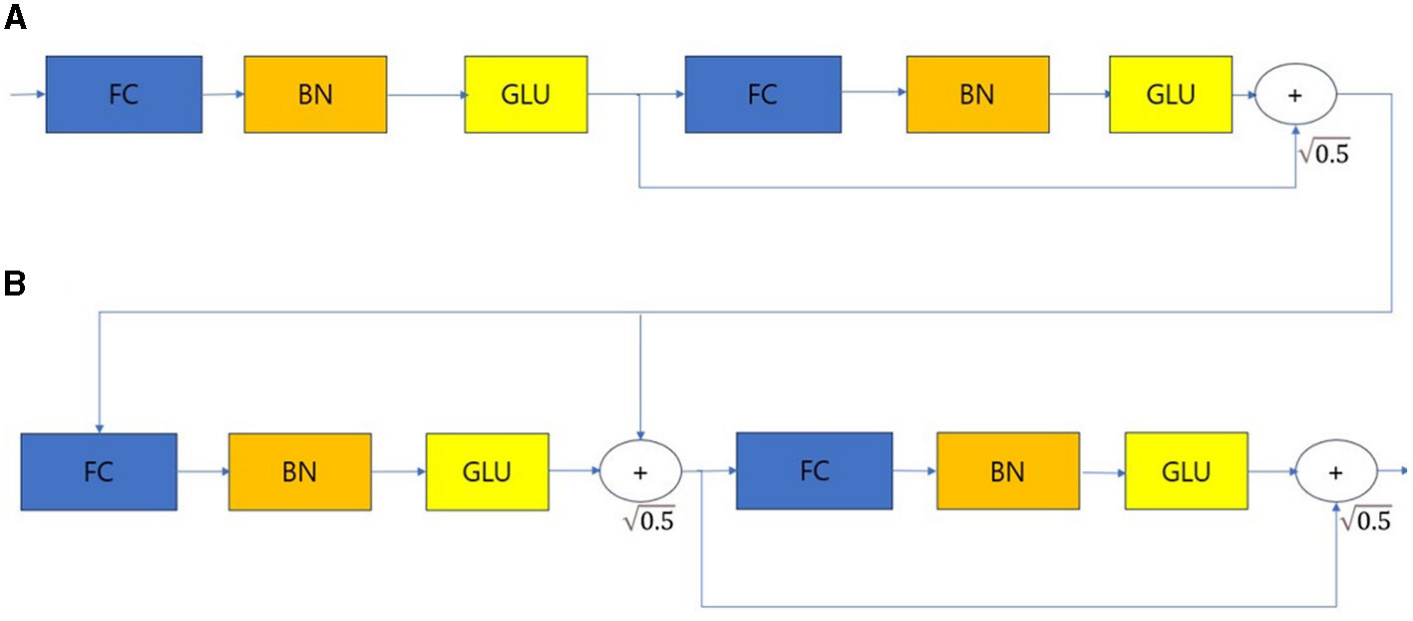
Feature transformer. **(A)** Shared across decision steps. **(B)** Decision step dependent steps. FC, Fully-connected layer; BN, Batch normalization; GLU, Gated linear unit.

The overall architecture is divided into the input part and decision steps from steps 1 to N, and each step consists of a feature transformer, attentive transformer, and feature masking. The split block divides the representation from the feature transformer into two, one sends to ReLU and the final output, and the other one is passed to the next attentive transformer. The mask block, which selects features, can provide information about how the features operate at each step, and the Agg block can inform us which features are important. Tabular data is used as input after calculating Min-Max or standard scaling, and normalization is replaced by the Batch Normalization layer. Normalized input is passed to the feature transformer block (17–20). The feature transformer block is composed as shown in Figure 3.

**Figure 4:**
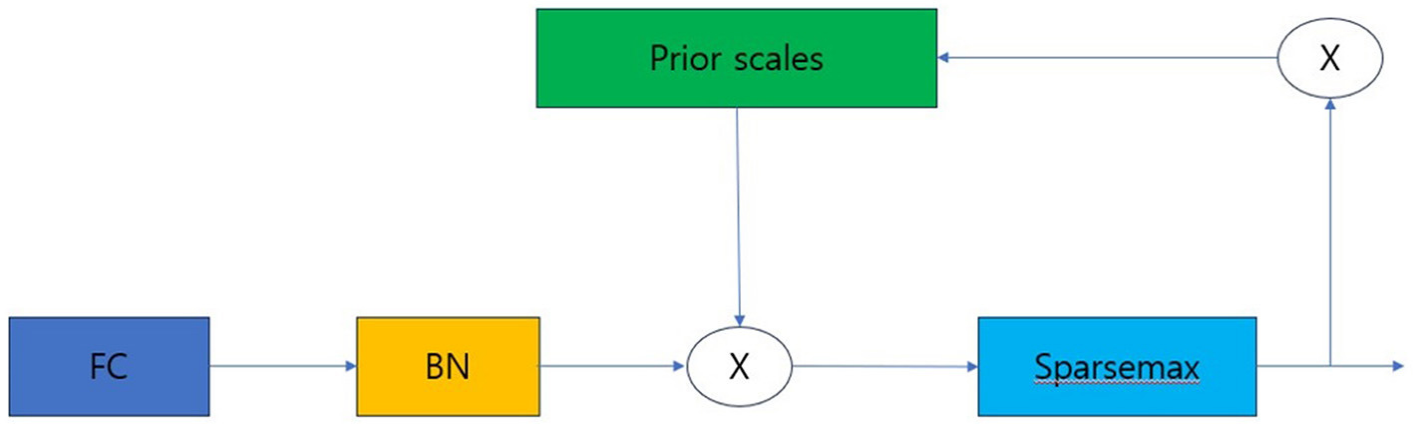
Attentive transformer. FC, Fully-connected layer; BN, Batch normalization.

The feature transformer consists of shared across decision steps and decision step dependent blocks. FC-BN-GLU is repeated four times. Among them, the first two blocks are shared in all decision steps, and the last two blocks are used only in decision step. The Attentive transformer block is structured as shown in Figure 4.

Prior scale is the information that aggregates how much each feature was used in previous decision steps. A Sparse Mask must be learned to select important features from the previous steps. Additionally, through masking, the influence of variables that do not have a significant impact on learning must be reduced during the decision step process. To implement such a mask, the attentive transformer is used. The notation for obtaining the mask is as follows.


(1) $$\begin{array}{l} M\left[i\right]=sparsemax\left(P\left[i-1\right]*hi\left(a\left[i-1\right]\right)\right) \end{array}$$

(2) $$\begin{array}{l} P\left[i\right]=\overset{i}{\underset{j=1}{\prod }}\gamma -M\left[j\right] \end{array}$$

In Equation (1), the Mask M[i] performs normalization called Sparsemax. This is a method that allows it to select the most salient features at each decision step. In Equation (2), P[i] denotes the prior scales, which creates a new mask by considering the influence of the feature and masks processed from the previous decision step. Gamma is a relaxation parameter. When gamma is 1, the feature is used only in one decision step. As gamma increases, the hyper-parameter allows the feature to be used in multiple decision steps (21–23).

To train TabNet, the entire cohort was randomly divided into a training cohort and a validation cohort (80:20). Data from the validation cohort was not used for training but was used to verify the performance of the trained model. In our experiment, classification accuracy was calculated as an indicator to verify the model's performance, which is derived from the confusion matrix. To train the TabNet model, training/validation/test data were randomly selected at the ratios of 70%, 20%, and 10%. The training epoch was 100 and the model was built using the TabNet classification function (version: 4.1.0).

### 2.4 Statistical analysis

Continuous variables are presented as median with range and categorical variables are expressed as numbers with ratio. Discrete variables (gender, hepatitis virus infection, cirrhosis, operation history, laparotomy, liver resection, splenectomy, TACE, RFA, satellite lesion, microvascular invasion, portal vein tumor thrombus, and biliary tumor thrombus) was defined as absence or presence, with absence mapped to 0 and presence to 1, respectively. These variables were used to train TabNet model. Continuous variables (age, AFP, CA199, blood loss, and blood transfusion) were entered into TabNet and survival analysis without preprocessing. In addition to improving classification accuracy through TabNet model training, we implemented a model that tracks patient survival using R (version 4.2.3) code to verify the performance of our newly built model. It was compared with the case where each condition of the Milan scale was applied, and the patient's survival probability was plotted against the survival period. As performance evaluation indicators for the model, C-index, Hazard ratio, *p*-value, AUC (Area under Curve), and NRI (Net Reclassification Index) were calculated (24, 25). All statistical tests were two-sided, and *p* values <0.05 were considered statistically significant.

## 3 Result

### 3.1 Patient clinicopathological characteristics

As shown in the flow chart (Figure 1), a total of 356 patients who underwent LT for HCC from January 2015 to December 2018 were enrolled in the study. The baseline characteristics of patients were described in Table 1. The median age of included patients was 52 years (22–78 years), and 315 (88.5%) were male. The majority of patients (322, 90.4%) had infection of hepatitis B virus. The median preoperative AFP level was 58.5 ng/ml. Nearly half of patients received pre-transplant surgery or therapy to control tumor development including liver resection, TACE and RFA. Through postoperative pathology, 312 patients had presence of cirrhosis (312, 87.6%). The median tumor max diameter was 4 cm (0.4–24 cm) and multiple tumors were present in 161 patients (45.2%) with 15.1% had satellite lesions. Further, microvascular invasion and portal vein tumor thrombus were confirmed in 151 (42.4%) and 75 patients (21.1%), respectively.

**Table 1:** Clinicopathological characteristics of HCC patients.

**Clinicopathological characteristics**	***n* (%)**
Total patients	356
Age (years) (median, range)	52 (22–78)
Gender (Male)	315 (88.5)
Gender (Female)	41 (11.5)
Presence of HBV infection	322 (90.4)
Presence of HCV infection	8 (2.2)
Preoperative AFP level (ng/ml) (median, range)	58.5 (0.7–27,594)
Preoperative CA199 level (U/ml) (median, range)	25.15 (0.6–2,492)
Presence of cirrhosis	312 (87.6)
Operation history	136 (38.2)
Presence of laparotomy	68 (19.1)
Liver resection	51 (13.6)
Splenectomy	14 (3.9)
TACE	71 (19.9)
RFA	30 (8.4)
Blood loss	500 (0–10,000)
Blood transfusion	0 (0–9,600)
Multiple tumor numbers	161 (45.2)
Presence of satellite lesion	54 (15.1)
Tumor max diameter(cm) (median, range)	4 (0.4–24)
**Pathological grade**	
I	7 (1.9)
I–II	14 (3.9)
II	201 (56.7)
II–III	66 (18.5)
III	67 (18.8)
III–IV	0 (0)
IV	1 (0.2)
Presence of microvascular invasion	151 (42.4)
Presence of portal vein tumor thrombus location of portal vein tumor thrombus	75 (21.1)
None	281 (78.9)
Second-order branches of the portal vein	13 (3.7)
Right or left portal vein	33 (9.3)
Main trunk	29 (8.1)
Presence of biliary tumor thrombus	21 (5.8)
Presence of vena cava invasion	12 (3.3)

### 3.2 Extraction of patient characteristics based on survival probability

A total of 356 HCC patients who received LT with complete follow-up data were evaluated. The entire cohort was randomly divided into training set (*n* = 286) and validation set (*n* = 70). Multi-layer-perceptron model provided by Pycox library was first used to construct the recurrence prediction model. Then validation data was input to verify the performance of the model. Patients were finally classified as group of high-risk recurrence with a 5-year survival probability of <20% and low-risk with survival more than 70%. Patients who had larger tumor size over 7 cm, poorer differentiation of tumor grade and multiple tumor numbers were determined as high-risk group, while patients with tumor size <2 cm, higher tumor differentiation grade and single tumor were regarded as low-risk group (Table 2). Compared with low-risk group, patients in the high-risk group were more likely to have higher level of preoperative AFP level, more operative blood loss and transfusion, more satellite lesion, microvascular invasion, presence of portal vein tumor thrombus, and biliary tumor thrombus (Table 3).

**Table 2:** Features of patients with different risks.

	**Low-risk**	**High-risk**
Tumor size (cm)	2.45	7.21
Tumor number	Single	Multi
Pathological grade	Lower than stage II	Higher than stage II-III

**Table 3:** The baseline comparison of patients with high and low risk of recurrence receiving LT with HCC.

**Variable**	**Low risk**	**High risk**	***P* value**
Age, median (years)	55	51.9	0.0183
Gender, male (%)	91	91.6	0.922
AFP, median (ng/ml)	193.2	674.5	0.021
CA199, median (U/ml)	33.1	49.9	0.425
HBV infection, (%)	100	97	0.831
HCV infection, (%)	0	8.3	0.818
Cirrhosis, (%)	100	92	0.470
Laparotomy, (%)	18.2	17	0.952
Liver resection, (%)	12	17	0.847
TACE, (%)	9.1	8.3	0.741
Blood loss, median, (ml)	400	966.7	0.014
Blood transfusion, median, (ml)	363.6	1,000	<0.05
Satellite lesion, (%)	12.3	41.6	0.006
Microvascular invasion, (%)	25.8	83.3	<0.001
Presence of portal vein tumor thrombus, (%)	0	50	<0.001
Presence of biliary tumor thrombus, (%)	0	16.7	<0.001

### 3.3 Results of classification by TabNet

**Figure 5:**
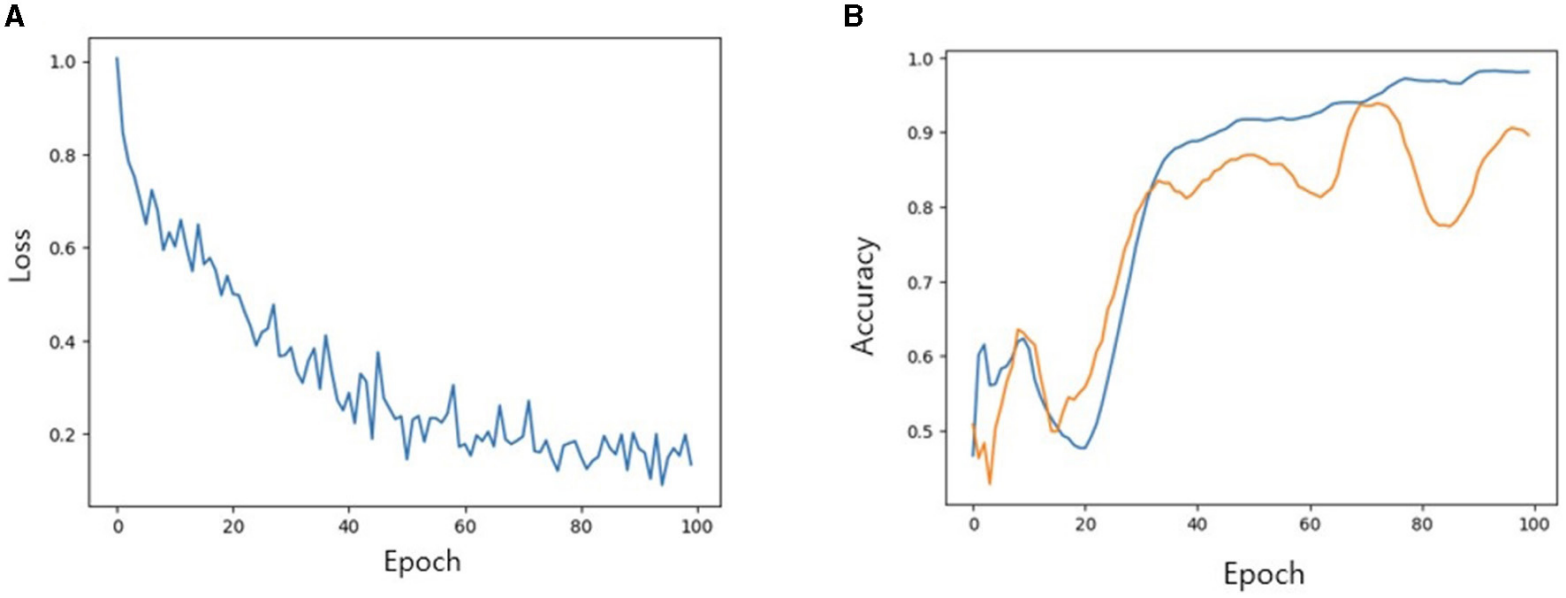
**(A)** Loss trend. **(B)** Accuracy of training and validation of models when Milan criteria is applied. In **(B)** blue line: training accuracy; yellow line: validation accuracy.

**Figure 6:**
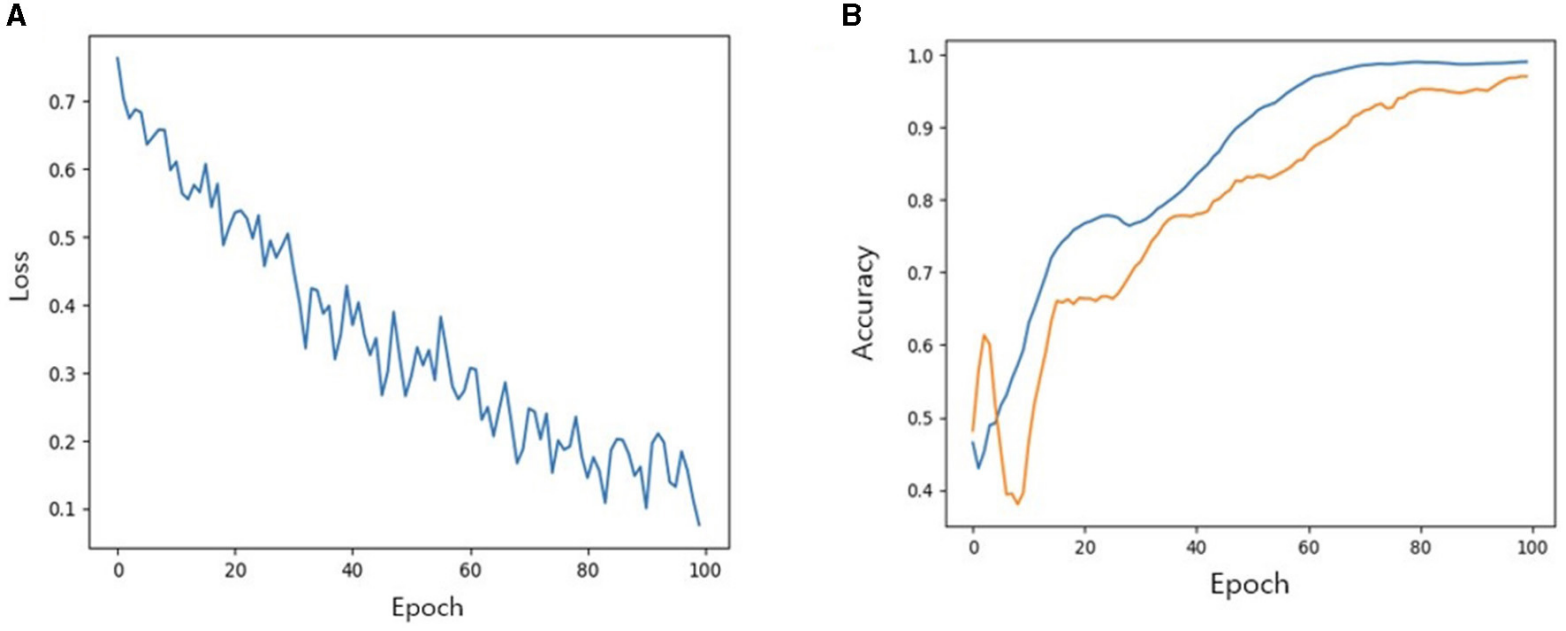
**(A)** Loss trend. **(B)** Accuracy of training and validation of models when new conditions is applied. In **(B)** blue line: training accuracy; yellow line: validation accuracy.

We then investigated the performance of our model with TabNet in improving the accuracy of the classification model. All cases were randomly split into training, validation and test set at a ratio of 7:2:1. The accuracy of training/validation set was observed for each epoch, and the final accuracy was calculated using test set. To increase training speed, a GPU (GeForce RTX 4060 TI) was used, and training/validation accuracy was observed for each epoch. As the epoch increased, loss tended to decrease, and training accuracy and verification accuracy gradually increased from negligible levels, which confirmed that the training was stable. As a result of measuring the final accuracy of the trained TabNet classification model by applying the Milan scale, the classification accuracy was 86% (Figure 5). On the other hand, after training our new condition based on risk classification, the accuracy was improved to 95% (Figure 6).

### 3.4 Survival analysis of the model by TabNet

**Figure 7:**
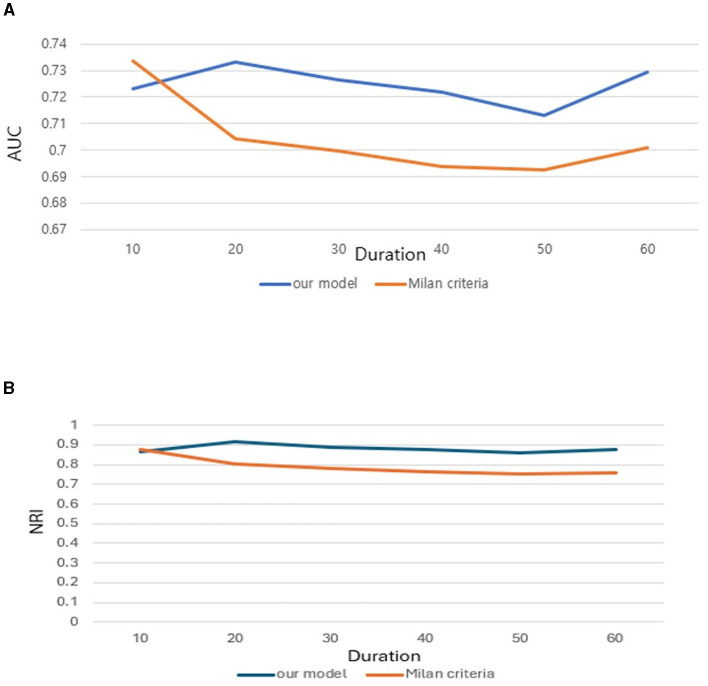
**(A)** Area Under Curve (AUC). **(B)** Net Reclassification Improvement (NRI) of our new conditions comparing with Milan criteria.

**Figure 8:**
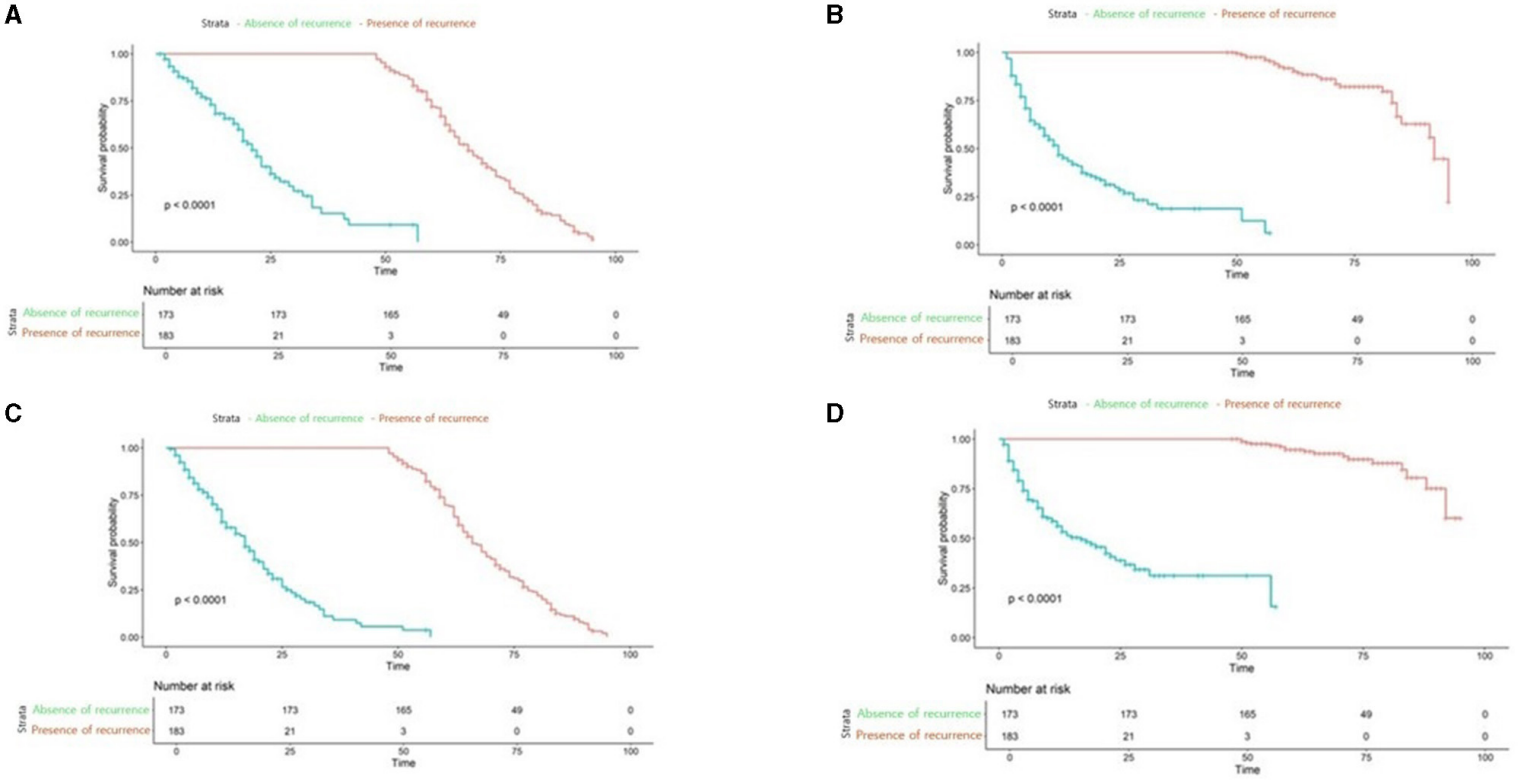
Survival analysis plot of applied to our new conditions, Milan criteria **(A)** our new model applied to Low-risk patients **(B)** our new model applied to high-risk patients **(C)** Milan Criteria applied to low-risk patients **(D)** Milan Criteria applied to High-risk patients.

Further we aimed to confirm how much tracking ability was improved in patient survival analysis and to compare the performance of the model with TabNet to that of the Milan criteria. Both time-dependent AUC value and NRI value were significant for our model, confirming its superiority over other conditions in patient survival analysis (Tables 4, 5). The AUC of the survival analysis model built for each condition was calculated, and the NRI was measured based on the model set based on whether the AFP exceeds 100 ng/ml (Figure 7). The patients' survival curve for each survival model was shown in Figure 8.

**Table 4:** Comparison of the AUC value of survival analysis between our model and Milan criteria.

**Duration (months)**	**Our model**	**Milan criteria**
10	0.7233 [0.6715–0.7751]	0.7334 [0.6812–0.7856]
20	0.7331 [0.6863–0.7799]	0.7041 [0.6582–0.75]
30	0.7263 [0.6806–0.772]	0.6998 [0.6563–0.7433]
40	0.7219 [0.6764–0.7674]	0.6938 [0.6509–0.7368]
50	0.7132 [0.666–0.7599]	0.6925 [0.6495–0.7357]
60	0.7296 [0.681–0.7783]	0.701 [0.6569–0.7457]

**Table 5:** Comparison of the time-dependent NRI value between our model and Milan criteria.

**Duration (months)**	**AFP (>100 ng/ml)**	**Our model**	**Milan criteria**
10	Reference	0.8637 [0.6059–0.9415]	0.8749 [0.6751–0.9796]
20	Reference	0.9147 [0.6658–1.032]	0.8024 [0.5501–0.9074]
30	Reference	0.8862 [0.5824–0.9985]	0.7818 [0.6336–0.8926]
40	Reference	0.8764 [0.7622–0.9845]	0.764 [0.5209–0.9254]
50	Reference	0.8585 [0.7043–1.0494]	0.751 [0.5354–0.8719]
60	Reference	0.8777 [0.642–1.042]	0.7565 [0.6554–0.9345]

## 4 Discussion

Though LT provides curative therapeutic option for HCC, tumor recurrence remains one of the leading problems for long-term prognosis of LT. Traditional Milan criteria based on morphological characteristics is unable to provide a quantification of HCC recurrence risk. Other prediction models on account of mathematical statistics analysis failed to predict tumor recurrence precisely as well (26–28). Therefore, a more efficient model predicting HCC recurrence after LT is urgently needed.

In this study including 356 HCC patients receiving LT, we proposed a survival prediction model based on deep learning algorithm with high accuracy, superior to the traditional Milan criteria. Our work first evaluated prognostic significance of clinical parameters and classified patients with larger tumor size over 7 cm, poorer differentiation of tumor grade and multiple tumor numbers as high risk of recurrence. Then a classification model was trained with TabNet and our proposed model outperformed the Milan criteria in terms of accuracy (0.95 vs. 0.86, *p* < 0.05). In addition, our model showed better performance results with improved AUC, NRI and hazard ratio, proving the robustness of the model.

Compared with previous HCC recurrence prediction models, our model based on TabNet was characterized by an improvement of methodology and prediction accuracy. TabNet is a novel deep learning model specialized for extracting a subset of semantically important features from tabular data such as patient information. Although several studies have been reported using TabNet, there are few reports in the medical field constructing a tumor recurrence prediction model (29, 30). Previous studies have established HCC prognostic models after resection. However, these models were based on weakly supervised network of categories and few reports were associated with HCC recurrence after LT (31, 32). In our study, more tumor categories instead of only morphological parameters were input to our model, which enhanced the diversity during feature extraction. In addition, the TabNet consisting of multiple-step decision units (attentive transformer, feature transformer, spilt, and ReLU function) greatly improved the automatic learning capability of providing predicting analyses. Therefore, we achieved better predictive performance and model convergence than the traditional Milan criteria.

Recently, Qu et al. established a deep pathomics score for predicting tumor recurrence after LT (33). The study mainly focused on structural and cellular significance of immune cells of LT patients, which was different from our study. Since multiple therapies including TACE, multi-kinase inhibitors and immune checkpoint inhibitors were increasingly used before LT, different tumor immune microenvironment predicting tumor recurrence should be given more attention. Additionally, subgroups of immune cells change with immunosuppressants after LT patients, which needs further exploration of its impact on prognosis. Future studies could focus on the mechanism of regulating immune cells and crosstalk between different cell grouping based on deep learning algorithm.

There are several limitations of our study to be noted. Firstly, it is a retrospective study with a limited number of patients coming from a single institution. External validation from other centers is needed to confirm the results. Second, the imbalance of clinicopathological characteristics including gender, HBV infection, etc. may cause selection bias and reduce the representativeness of our population. Additionally, medical imaging plays an important role and has been widely used in oncological deep learning, but was not explored in this study. Our future study will combine imaging information and immune-pathological profiles to further validate our prediction model via deep learning computing.

In conclusion, our study proposed an efficient tumor recurrence model for HCC patients after LT based on deep learning algorithm. Our model outperformed Milan criteria in guiding HCC surveillance strategies by predicting tumor recurrence and survival. Future studies should focus on the correlation between imaging information and immune-pathological profiles.

## Data availability statement

The original contributions presented in the study are included in the article/Supplementary material, further inquiries can be directed to the corresponding authors.

## Ethics statement

The manuscript presents research on animals that do not require ethical approval for their study.

## Author contributions

SK: Conceptualization, Data curation, Formal analysis, Investigation, Methodology, Project administration, Software, Validation, Visualization, Writing – original draft, Writing – review & editing. JC: Formal analysis, Project administration, Validation, Writing – review & editing. Y-kY: Data curation, Formal analysis, Methodology, Project administration, Validation, Writing – review & editing. Z-fX: Data curation, Formal analysis, Methodology, Project administration, Validation, Writing – review & editing. HH: Supervision, Writing – review & editing. MS: Conceptualization, Data curation, Formal analysis, Funding acquisition, Investigation, Methodology, Project administration, Resources, Supervision, Writing – original draft, Writing – review & editing. QX: Supervision, Writing – review & editing.
