# Supplementary material for: Development of a deep learning model for predicting recurrence of hepatocellular carcinoma after liver transplantation
Source: Front Med (Lausanne). 2024 Jun 11;11:1373005. doi: 10.3389/fmed.2024.1373005 (PMC11196752; doi:10.3389/fmed.2024.1373005)
Supplement: Supplementary file 1 [file Data_Sheet_1.ZIP › Raw data/source data and codes/codes/tabnet/docs/_modules/index.html]

Overview: module code — pytorch\_tabnet documentation


pytorch\_tabnet

Contents:

- README
- TabNet : Attentive Interpretable Tabular Learning
- Installation
- What is new ?
- Contributing
- What problems does pytorch-tabnet handle?
- How to use it?
- Semi-supervised pre-training
- Data augmentation on the fly
- Easy saving and loading
- Useful links
- pytorch\_tabnet package

pytorch\_tabnet

- »
- Overview: module code

---

# All modules for which code is available

- pytorch\_tabnet.abstract\_model
- pytorch\_tabnet.augmentations
- pytorch\_tabnet.callbacks
- pytorch\_tabnet.metrics
- pytorch\_tabnet.multiclass\_utils
- pytorch\_tabnet.multitask
- pytorch\_tabnet.pretraining
- pytorch\_tabnet.pretraining\_utils
- pytorch\_tabnet.sparsemax
- pytorch\_tabnet.tab\_model
- pytorch\_tabnet.tab\_network
- pytorch\_tabnet.utils
- torch.optim.adam

---

© Copyright 2019, Dreamquark

Built with Sphinx using a
theme
provided by Read the Docs.
