# Supplementary material for: Development of a deep learning model for predicting recurrence of hepatocellular carcinoma after liver transplantation
Source: Front Med (Lausanne). 2024 Jun 11;11:1373005. doi: 10.3389/fmed.2024.1373005 (PMC11196752; doi:10.3389/fmed.2024.1373005)
Supplement: Supplementary file 1 [file Data_Sheet_1.ZIP › Raw data/source data and codes/codes/tabnet/docs/_modules/pytorch_tabnet/abstract_model.html]

pytorch\_tabnet.abstract\_model — pytorch\_tabnet documentation


pytorch\_tabnet

Contents:

- README
- TabNet : Attentive Interpretable Tabular Learning
- Installation
- What is new ?
- Contributing
- What problems does pytorch-tabnet handle?
- How to use it?
- Semi-supervised pre-training
- Data augmentation on the fly
- Easy saving and loading
- Useful links
- pytorch\_tabnet package

pytorch\_tabnet

- »
- Module code »
- pytorch\_tabnet.abstract\_model

---

# Source code for pytorch\_tabnet.abstract\_model

```
from dataclasses import dataclass, field
from typing import List, Any, Dict
import torch
from torch.nn.utils import clip_grad_norm_
import numpy as np
from scipy.sparse import csc_matrix
from abc import abstractmethod
from pytorch_tabnet import tab_network
from pytorch_tabnet.utils import (
    SparsePredictDataset,
    PredictDataset,
    create_explain_matrix,
    validate_eval_set,
    create_dataloaders,
    define_device,
    ComplexEncoder,
    check_input,
    check_warm_start,
    create_group_matrix,
    check_embedding_parameters
)
from pytorch_tabnet.callbacks import (
    CallbackContainer,
    History,
    EarlyStopping,
    LRSchedulerCallback,
)
from pytorch_tabnet.metrics import MetricContainer, check_metrics
from sklearn.base import BaseEstimator

from torch.utils.data import DataLoader
import io
import json
from pathlib import Path
import shutil
import zipfile
import warnings
import copy
import scipy

[docs]@dataclass
class TabModel(BaseEstimator):
    """ Class for TabNet model."""

    n_d: int = 8
    n_a: int = 8
    n_steps: int = 3
    gamma: float = 1.3
    cat_idxs: List[int] = field(default_factory=list)
    cat_dims: List[int] = field(default_factory=list)
    cat_emb_dim: int = 1
    n_independent: int = 2
    n_shared: int = 2
    epsilon: float = 1e-15
    momentum: float = 0.02
    lambda_sparse: float = 1e-3
    seed: int = 0
    clip_value: int = 1
    verbose: int = 1
    optimizer_fn: Any = torch.optim.Adam
    optimizer_params: Dict = field(default_factory=lambda: dict(lr=2e-2))
    scheduler_fn: Any = None
    scheduler_params: Dict = field(default_factory=dict)
    mask_type: str = "sparsemax"
    input_dim: int = None
    output_dim: int = None
    device_name: str = "auto"
    n_shared_decoder: int = 1
    n_indep_decoder: int = 1
    grouped_features: List[List[int]] = field(default_factory=list)

    def __post_init__(self):
        # These are default values needed for saving model
        self.batch_size = 1024
        self.virtual_batch_size = 128

        torch.manual_seed(self.seed)
        # Defining device
        self.device = torch.device(define_device(self.device_name))
        if self.verbose != 0:
            warnings.warn(f"Device used : {self.device}")

        # create deep copies of mutable parameters
        self.optimizer_fn = copy.deepcopy(self.optimizer_fn)
        self.scheduler_fn = copy.deepcopy(self.scheduler_fn)

        updated_params = check_embedding_parameters(self.cat_dims,
                                                    self.cat_idxs,
                                                    self.cat_emb_dim)
        self.cat_dims, self.cat_idxs, self.cat_emb_dim = updated_params

    def __update__(self, **kwargs):
        """
        Updates parameters.
        If does not already exists, creates it.
        Otherwise overwrite with warnings.
        """
        update_list = [
            "cat_dims",
            "cat_emb_dim",
            "cat_idxs",
            "input_dim",
            "mask_type",
            "n_a",
            "n_d",
            "n_independent",
            "n_shared",
            "n_steps",
            "grouped_features",
        ]
        for var_name, value in kwargs.items():
            if var_name in update_list:
                try:
                    exec(f"global previous_val; previous_val = self.{var_name}")
                    if previous_val != value:  # noqa
                        wrn_msg = f"Pretraining: {var_name} changed from {previous_val} to {value}"  # noqa
                        warnings.warn(wrn_msg)
                        exec(f"self.{var_name} = value")
                except AttributeError:
                    exec(f"self.{var_name} = value")

[docs]    def fit(
        self,
        X_train,
        y_train,
        eval_set=None,
        eval_name=None,
        eval_metric=None,
        loss_fn=None,
        weights=0,
        max_epochs=100,
        patience=10,
        batch_size=1024,
        virtual_batch_size=128,
        num_workers=0,
        drop_last=True,
        callbacks=None,
        pin_memory=True,
        from_unsupervised=None,
        warm_start=False,
        augmentations=None,
        compute_importance=True
    ):
        """Train a neural network stored in self.network
        Using train_dataloader for training data and
        valid_dataloader for validation.

        Parameters
        ----------
        X_train : np.ndarray
            Train set
        y_train : np.array
            Train targets
        eval_set : list of tuple
            List of eval tuple set (X, y).
            The last one is used for early stopping
        eval_name : list of str
            List of eval set names.
        eval_metric : list of str
            List of evaluation metrics.
            The last metric is used for early stopping.
        loss_fn : callable or None
            a PyTorch loss function
        weights : bool or dictionnary
            0 for no balancing
            1 for automated balancing
            dict for custom weights per class
        max_epochs : int
            Maximum number of epochs during training
        patience : int
            Number of consecutive non improving epoch before early stopping
        batch_size : int
            Training batch size
        virtual_batch_size : int
            Batch size for Ghost Batch Normalization (virtual_batch_size < batch_size)
        num_workers : int
            Number of workers used in torch.utils.data.DataLoader
        drop_last : bool
            Whether to drop last batch during training
        callbacks : list of callback function
            List of custom callbacks
        pin_memory: bool
            Whether to set pin_memory to True or False during training
        from_unsupervised: unsupervised trained model
            Use a previously self supervised model as starting weights
        warm_start: bool
            If True, current model parameters are used to start training
        compute_importance : bool
            Whether to compute feature importance
        """
        # update model name

        self.max_epochs = max_epochs
        self.patience = patience
        self.batch_size = batch_size
        self.virtual_batch_size = virtual_batch_size
        self.num_workers = num_workers
        self.drop_last = drop_last
        self.input_dim = X_train.shape[1]
        self._stop_training = False
        self.pin_memory = pin_memory and (self.device.type != "cpu")
        self.augmentations = augmentations
        self.compute_importance = compute_importance

        if self.augmentations is not None:
            # This ensure reproducibility
            self.augmentations._set_seed()

        eval_set = eval_set if eval_set else []

        if loss_fn is None:
            self.loss_fn = self._default_loss
        else:
            self.loss_fn = loss_fn

        check_input(X_train)
        check_warm_start(warm_start, from_unsupervised)

        self.update_fit_params(
            X_train,
            y_train,
            eval_set,
            weights,
        )

        # Validate and reformat eval set depending on training data
        eval_names, eval_set = validate_eval_set(eval_set, eval_name, X_train, y_train)

        train_dataloader, valid_dataloaders = self._construct_loaders(
            X_train, y_train, eval_set
        )

        if from_unsupervised is not None:
            # Update parameters to match self pretraining
            self.__update__(**from_unsupervised.get_params())

        if not hasattr(self, "network") or not warm_start:
            # model has never been fitted before of warm_start is False
            self._set_network()
        self._update_network_params()
        self._set_metrics(eval_metric, eval_names)
        self._set_optimizer()
        self._set_callbacks(callbacks)

        if from_unsupervised is not None:
            self.load_weights_from_unsupervised(from_unsupervised)
            warnings.warn("Loading weights from unsupervised pretraining")
        # Call method on_train_begin for all callbacks
        self._callback_container.on_train_begin()

        # Training loop over epochs
        for epoch_idx in range(self.max_epochs):

            # Call method on_epoch_begin for all callbacks
            self._callback_container.on_epoch_begin(epoch_idx)

            self._train_epoch(train_dataloader)

            # Apply predict epoch to all eval sets
            for eval_name, valid_dataloader in zip(eval_names, valid_dataloaders):
                self._predict_epoch(eval_name, valid_dataloader)

            # Call method on_epoch_end for all callbacks
            self._callback_container.on_epoch_end(
                epoch_idx, logs=self.history.epoch_metrics
            )

            if self._stop_training:
                break

        # Call method on_train_end for all callbacks
        self._callback_container.on_train_end()
        self.network.eval()

        if self.compute_importance:
            # compute feature importance once the best model is defined
            self.feature_importances_ = self._compute_feature_importances(X_train)


[docs]    def predict(self, X):
        """
        Make predictions on a batch (valid)

        Parameters
        ----------
        X : a :tensor: `torch.Tensor` or matrix: `scipy.sparse.csr_matrix`
            Input data

        Returns
        -------
        predictions : np.array
            Predictions of the regression problem
        """
        self.network.eval()

        if scipy.sparse.issparse(X):
            dataloader = DataLoader(
                SparsePredictDataset(X),
                batch_size=self.batch_size,
                shuffle=False,
            )
        else:
            dataloader = DataLoader(
                PredictDataset(X),
                batch_size=self.batch_size,
                shuffle=False,
            )

        results = []
        for batch_nb, data in enumerate(dataloader):
            data = data.to(self.device).float()
            output, M_loss = self.network(data)
            predictions = output.cpu().detach().numpy()
            results.append(predictions)
        res = np.vstack(results)
        return self.predict_func(res)


[docs]    def explain(self, X, normalize=False):
        """
        Return local explanation

        Parameters
        ----------
        X : tensor: `torch.Tensor` or matrix: `scipy.sparse.csr_matrix`
            Input data
        normalize : bool (default False)
            Wheter to normalize so that sum of features are equal to 1

        Returns
        -------
        M_explain : matrix
            Importance per sample, per columns.
        masks : matrix
            Sparse matrix showing attention masks used by network.
        """
        self.network.eval()

        if scipy.sparse.issparse(X):
            dataloader = DataLoader(
                SparsePredictDataset(X),
                batch_size=self.batch_size,
                shuffle=False,
            )
        else:
            dataloader = DataLoader(
                PredictDataset(X),
                batch_size=self.batch_size,
                shuffle=False,
            )

        res_explain = []

        for batch_nb, data in enumerate(dataloader):
            data = data.to(self.device).float()

            M_explain, masks = self.network.forward_masks(data)
            for key, value in masks.items():
                masks[key] = csc_matrix.dot(
                    value.cpu().detach().numpy(), self.reducing_matrix
                )
            original_feat_explain = csc_matrix.dot(M_explain.cpu().detach().numpy(),
                                                   self.reducing_matrix)
            res_explain.append(original_feat_explain)

            if batch_nb == 0:
                res_masks = masks
            else:
                for key, value in masks.items():
                    res_masks[key] = np.vstack([res_masks[key], value])

        res_explain = np.vstack(res_explain)

        if normalize:
            res_explain /= np.sum(res_explain, axis=1)[:, None]

        return res_explain, res_masks


[docs]    def load_weights_from_unsupervised(self, unsupervised_model):
        update_state_dict = copy.deepcopy(self.network.state_dict())
        for param, weights in unsupervised_model.network.state_dict().items():
            if param.startswith("encoder"):
                # Convert encoder's layers name to match
                new_param = "tabnet." + param
            else:
                new_param = param
            if self.network.state_dict().get(new_param) is not None:
                # update only common layers
                update_state_dict[new_param] = weights

        self.network.load_state_dict(update_state_dict)


[docs]    def load_class_attrs(self, class_attrs):
        for attr_name, attr_value in class_attrs.items():
            setattr(self, attr_name, attr_value)


[docs]    def save_model(self, path):
        """Saving TabNet model in two distinct files.

        Parameters
        ----------
        path : str
            Path of the model.

        Returns
        -------
        str
            input filepath with ".zip" appended

        """
        saved_params = {}
        init_params = {}
        for key, val in self.get_params().items():
            if isinstance(val, type):
                # Don't save torch specific params
                continue
            else:
                init_params[key] = val
        saved_params["init_params"] = init_params

        class_attrs = {
            "preds_mapper": self.preds_mapper
        }
        saved_params["class_attrs"] = class_attrs

        # Create folder
        Path(path).mkdir(parents=True, exist_ok=True)

        # Save models params
        with open(Path(path).joinpath("model_params.json"), "w", encoding="utf8") as f:
            json.dump(saved_params, f, cls=ComplexEncoder)

        # Save state_dict
        torch.save(self.network.state_dict(), Path(path).joinpath("network.pt"))
        shutil.make_archive(path, "zip", path)
        shutil.rmtree(path)
        print(f"Successfully saved model at {path}.zip")
        return f"{path}.zip"


[docs]    def load_model(self, filepath):
        """Load TabNet model.

        Parameters
        ----------
        filepath : str
            Path of the model.
        """
        try:
            with zipfile.ZipFile(filepath) as z:
                with z.open("model_params.json") as f:
                    loaded_params = json.load(f)
                    loaded_params["init_params"]["device_name"] = self.device_name
                with z.open("network.pt") as f:
                    try:
                        saved_state_dict = torch.load(f, map_location=self.device)
                    except io.UnsupportedOperation:
                        # In Python <3.7, the returned file object is not seekable (which at least
                        # some versions of PyTorch require) - so we'll try buffering it in to a
                        # BytesIO instead:
                        saved_state_dict = torch.load(
                            io.BytesIO(f.read()),
                            map_location=self.device,
                        )
        except KeyError:
            raise KeyError("Your zip file is missing at least one component")

        self.__init__(**loaded_params["init_params"])

        self._set_network()
        self.network.load_state_dict(saved_state_dict)
        self.network.eval()
        self.load_class_attrs(loaded_params["class_attrs"])

        return

def _train_epoch(self, train_loader):
        """
        Trains one epoch of the network in self.network

        Parameters
        ----------
        train_loader : a :class: `torch.utils.data.Dataloader`
            DataLoader with train set
        """
        self.network.train()

        for batch_idx, (X, y) in enumerate(train_loader):
            self._callback_container.on_batch_begin(batch_idx)

            batch_logs = self._train_batch(X, y)

            self._callback_container.on_batch_end(batch_idx, batch_logs)

        epoch_logs = {"lr": self._optimizer.param_groups[-1]["lr"]}
        self.history.epoch_metrics.update(epoch_logs)

        return

    def _train_batch(self, X, y):
        """
        Trains one batch of data

        Parameters
        ----------
        X : torch.Tensor
            Train matrix
        y : torch.Tensor
            Target matrix

        Returns
        -------
        batch_outs : dict
            Dictionnary with "y": target and "score": prediction scores.
        batch_logs : dict
            Dictionnary with "batch_size" and "loss".
        """
        batch_logs = {"batch_size": X.shape[0]}

        X = X.to(self.device).float()
        y = y.to(self.device).float()

        if self.augmentations is not None:
            X, y = self.augmentations(X, y)

        for param in self.network.parameters():
            param.grad = None

        output, M_loss = self.network(X)

        loss = self.compute_loss(output, y)
        # Add the overall sparsity loss
        loss = loss - self.lambda_sparse * M_loss

        # Perform backward pass and optimization
        loss.backward()
        if self.clip_value:
            clip_grad_norm_(self.network.parameters(), self.clip_value)
        self._optimizer.step()

        batch_logs["loss"] = loss.cpu().detach().numpy().item()

        return batch_logs

    def _predict_epoch(self, name, loader):
        """
        Predict an epoch and update metrics.

        Parameters
        ----------
        name : str
            Name of the validation set
        loader : torch.utils.data.Dataloader
                DataLoader with validation set
        """
        # Setting network on evaluation mode
        self.network.eval()

        list_y_true = []
        list_y_score = []

        # Main loop
        for batch_idx, (X, y) in enumerate(loader):
            scores = self._predict_batch(X)
            list_y_true.append(y)
            list_y_score.append(scores)

        y_true, scores = self.stack_batches(list_y_true, list_y_score)

        metrics_logs = self._metric_container_dict[name](y_true, scores)
        self.network.train()
        self.history.epoch_metrics.update(metrics_logs)
        return

    def _predict_batch(self, X):
        """
        Predict one batch of data.

        Parameters
        ----------
        X : torch.Tensor
            Owned products

        Returns
        -------
        np.array
            model scores
        """
        X = X.to(self.device).float()

        # compute model output
        scores, _ = self.network(X)

        if isinstance(scores, list):
            scores = [x.cpu().detach().numpy() for x in scores]
        else:
            scores = scores.cpu().detach().numpy()

        return scores

    def _set_network(self):
        """Setup the network and explain matrix."""
        torch.manual_seed(self.seed)

        self.group_matrix = create_group_matrix(self.grouped_features, self.input_dim)

        self.network = tab_network.TabNet(
            self.input_dim,
            self.output_dim,
            n_d=self.n_d,
            n_a=self.n_a,
            n_steps=self.n_steps,
            gamma=self.gamma,
            cat_idxs=self.cat_idxs,
            cat_dims=self.cat_dims,
            cat_emb_dim=self.cat_emb_dim,
            n_independent=self.n_independent,
            n_shared=self.n_shared,
            epsilon=self.epsilon,
            virtual_batch_size=self.virtual_batch_size,
            momentum=self.momentum,
            mask_type=self.mask_type,
            group_attention_matrix=self.group_matrix.to(self.device),
        ).to(self.device)

        self.reducing_matrix = create_explain_matrix(
            self.network.input_dim,
            self.network.cat_emb_dim,
            self.network.cat_idxs,
            self.network.post_embed_dim,
        )

    def _set_metrics(self, metrics, eval_names):
        """Set attributes relative to the metrics.

        Parameters
        ----------
        metrics : list of str
            List of eval metric names.
        eval_names : list of str
            List of eval set names.

        """
        metrics = metrics or [self._default_metric]

        metrics = check_metrics(metrics)
        # Set metric container for each sets
        self._metric_container_dict = {}
        for name in eval_names:
            self._metric_container_dict.update(
                {name: MetricContainer(metrics, prefix=f"{name}_")}
            )

        self._metrics = []
        self._metrics_names = []
        for _, metric_container in self._metric_container_dict.items():
            self._metrics.extend(metric_container.metrics)
            self._metrics_names.extend(metric_container.names)

        # Early stopping metric is the last eval metric
        self.early_stopping_metric = (
            self._metrics_names[-1] if len(self._metrics_names) > 0 else None
        )

    def _set_callbacks(self, custom_callbacks):
        """Setup the callbacks functions.

        Parameters
        ----------
        custom_callbacks : list of func
            List of callback functions.

        """
        # Setup default callbacks history, early stopping and scheduler
        callbacks = []
        self.history = History(self, verbose=self.verbose)
        callbacks.append(self.history)
        if (self.early_stopping_metric is not None) and (self.patience > 0):
            early_stopping = EarlyStopping(
                early_stopping_metric=self.early_stopping_metric,
                is_maximize=(
                    self._metrics[-1]._maximize if len(self._metrics) > 0 else None
                ),
                patience=self.patience,
            )
            callbacks.append(early_stopping)
        else:
            wrn_msg = "No early stopping will be performed, last training weights will be used."
            warnings.warn(wrn_msg)

        if self.scheduler_fn is not None:
            # Add LR Scheduler call_back
            is_batch_level = self.scheduler_params.pop("is_batch_level", False)
            scheduler = LRSchedulerCallback(
                scheduler_fn=self.scheduler_fn,
                scheduler_params=self.scheduler_params,
                optimizer=self._optimizer,
                early_stopping_metric=self.early_stopping_metric,
                is_batch_level=is_batch_level,
            )
            callbacks.append(scheduler)

        if custom_callbacks:
            callbacks.extend(custom_callbacks)
        self._callback_container = CallbackContainer(callbacks)
        self._callback_container.set_trainer(self)

    def _set_optimizer(self):
        """Setup optimizer."""
        self._optimizer = self.optimizer_fn(
            self.network.parameters(), **self.optimizer_params
        )

    def _construct_loaders(self, X_train, y_train, eval_set):
        """Generate dataloaders for train and eval set.

        Parameters
        ----------
        X_train : np.array
            Train set.
        y_train : np.array
            Train targets.
        eval_set : list of tuple
            List of eval tuple set (X, y).

        Returns
        -------
        train_dataloader : `torch.utils.data.Dataloader`
            Training dataloader.
        valid_dataloaders : list of `torch.utils.data.Dataloader`
            List of validation dataloaders.

        """
        # all weights are not allowed for this type of model
        y_train_mapped = self.prepare_target(y_train)
        for i, (X, y) in enumerate(eval_set):
            y_mapped = self.prepare_target(y)
            eval_set[i] = (X, y_mapped)

        train_dataloader, valid_dataloaders = create_dataloaders(
            X_train,
            y_train_mapped,
            eval_set,
            self.updated_weights,
            self.batch_size,
            self.num_workers,
            self.drop_last,
            self.pin_memory,
        )
        return train_dataloader, valid_dataloaders

    def _compute_feature_importances(self, X):
        """Compute global feature importance.

        Parameters
        ----------
        loader : `torch.utils.data.Dataloader`
            Pytorch dataloader.

        """
        M_explain, _ = self.explain(X, normalize=False)
        sum_explain = M_explain.sum(axis=0)
        feature_importances_ = sum_explain / np.sum(sum_explain)
        return feature_importances_

    def _update_network_params(self):
        self.network.virtual_batch_size = self.virtual_batch_size

[docs]    @abstractmethod
    def update_fit_params(self, X_train, y_train, eval_set, weights):
        """
        Set attributes relative to fit function.

        Parameters
        ----------
        X_train : np.ndarray
            Train set
        y_train : np.array
            Train targets
        eval_set : list of tuple
            List of eval tuple set (X, y).
        weights : bool or dictionnary
            0 for no balancing
            1 for automated balancing
        """
        raise NotImplementedError(
            "users must define update_fit_params to use this base class"
        )


[docs]    @abstractmethod
    def compute_loss(self, y_score, y_true):
        """
        Compute the loss.

        Parameters
        ----------
        y_score : a :tensor: `torch.Tensor`
            Score matrix
        y_true : a :tensor: `torch.Tensor`
            Target matrix

        Returns
        -------
        float
            Loss value
        """
        raise NotImplementedError(
            "users must define compute_loss to use this base class"
        )


[docs]    @abstractmethod
    def prepare_target(self, y):
        """
        Prepare target before training.

        Parameters
        ----------
        y : a :tensor: `torch.Tensor`
            Target matrix.

        Returns
        -------
        `torch.Tensor`
            Converted target matrix.
        """
        raise NotImplementedError(
            "users must define prepare_target to use this base class"
        )
```

---

© Copyright 2019, Dreamquark

Built with Sphinx using a
theme
provided by Read the Docs.
