# Supplementary material for: Development of a deep learning model for predicting recurrence of hepatocellular carcinoma after liver transplantation
Source: Front Med (Lausanne). 2024 Jun 11;11:1373005. doi: 10.3389/fmed.2024.1373005 (PMC11196752; doi:10.3389/fmed.2024.1373005)
Supplement: Supplementary file 1 [file Data_Sheet_1.ZIP › Raw data/source data and codes/codes/tabnet/docs/_modules/pytorch_tabnet/augmentations.html]

pytorch\_tabnet.augmentations — pytorch\_tabnet documentation


pytorch\_tabnet

Contents:

- README
- TabNet : Attentive Interpretable Tabular Learning
- Installation
- What is new ?
- Contributing
- What problems does pytorch-tabnet handle?
- How to use it?
- Semi-supervised pre-training
- Data augmentation on the fly
- Easy saving and loading
- Useful links
- pytorch\_tabnet package

pytorch\_tabnet

- »
- Module code »
- pytorch\_tabnet.augmentations

---

# Source code for pytorch\_tabnet.augmentations

```
import torch
from pytorch_tabnet.utils import define_device
import numpy as np

[docs]class RegressionSMOTE():
    """
    Apply SMOTE

    This will average a percentage p of the elements in the batch with other elements.
    The target will be averaged as well (this might work with binary classification
    and certain loss), following a beta distribution.
    """
    def __init__(self, device_name="auto", p=0.8, alpha=0.5, beta=0.5, seed=0):
        ""
        self.seed = seed
        self._set_seed()
        self.device = define_device(device_name)
        self.alpha = alpha
        self.beta = beta
        self.p = p
        if (p < 0.) or (p > 1.0):
            raise ValueError("Value of p should be between 0. and 1.")

    def _set_seed(self):
        torch.manual_seed(self.seed)
        np.random.seed(self.seed)
        return

    def __call__(self, X, y):
        batch_size = X.shape[0]
        random_values = torch.rand(batch_size, device=self.device)
        idx_to_change = random_values < self.p

        # ensure that first element to switch has probability > 0.5
        np_betas = np.random.beta(self.alpha, self.beta, batch_size) / 2 + 0.5
        random_betas = torch.from_numpy(np_betas).to(self.device).float()
        index_permute = torch.randperm(batch_size, device=self.device)

        X[idx_to_change] = random_betas[idx_to_change, None] * X[idx_to_change]
        X[idx_to_change] += (1 - random_betas[idx_to_change, None]) * X[index_permute][idx_to_change].view(X[idx_to_change].size()) # noqa

        y[idx_to_change] = random_betas[idx_to_change, None] * y[idx_to_change]
        y[idx_to_change] += (1 - random_betas[idx_to_change, None]) * y[index_permute][idx_to_change].view(y[idx_to_change].size()) # noqa

        return X, y


[docs]class ClassificationSMOTE():
    """
    Apply SMOTE for classification tasks.

    This will average a percentage p of the elements in the batch with other elements.
    The target will stay unchanged and keep the value of the most important row in the mix.
    """
    def __init__(self, device_name="auto", p=0.8, alpha=0.5, beta=0.5, seed=0):
        ""
        self.seed = seed
        self._set_seed()
        self.device = define_device(device_name)
        self.alpha = alpha
        self.beta = beta
        self.p = p
        if (p < 0.) or (p > 1.0):
            raise ValueError("Value of p should be between 0. and 1.")

    def _set_seed(self):
        torch.manual_seed(self.seed)
        np.random.seed(self.seed)
        return

    def __call__(self, X, y):
        batch_size = X.shape[0]
        random_values = torch.rand(batch_size, device=self.device)
        idx_to_change = random_values < self.p

        # ensure that first element to switch has probability > 0.5
        np_betas = np.random.beta(self.alpha, self.beta, batch_size) / 2 + 0.5
        random_betas = torch.from_numpy(np_betas).to(self.device).float()
        index_permute = torch.randperm(batch_size, device=self.device)

        X[idx_to_change] = random_betas[idx_to_change, None] * X[idx_to_change]
        X[idx_to_change] += (1 - random_betas[idx_to_change, None]) * X[index_permute][idx_to_change].view(X[idx_to_change].size())  # noqa

        return X, y
```

---

© Copyright 2019, Dreamquark

Built with Sphinx using a
theme
provided by Read the Docs.
