# Supplementary material for: Development of a deep learning model for predicting recurrence of hepatocellular carcinoma after liver transplantation
Source: Front Med (Lausanne). 2024 Jun 11;11:1373005. doi: 10.3389/fmed.2024.1373005 (PMC11196752; doi:10.3389/fmed.2024.1373005)
Supplement: Supplementary file 1 [file Data_Sheet_1.ZIP › Raw data/source data and codes/codes/tabnet/docs/_modules/pytorch_tabnet/callbacks.html]

pytorch\_tabnet.callbacks — pytorch\_tabnet documentation


pytorch\_tabnet

Contents:

- README
- TabNet : Attentive Interpretable Tabular Learning
- Installation
- What is new ?
- Contributing
- What problems does pytorch-tabnet handle?
- How to use it?
- Semi-supervised pre-training
- Data augmentation on the fly
- Easy saving and loading
- Useful links
- pytorch\_tabnet package

pytorch\_tabnet

- »
- Module code »
- pytorch\_tabnet.callbacks

---

# Source code for pytorch\_tabnet.callbacks

```
import time
import datetime
import copy
import numpy as np
from dataclasses import dataclass, field
from typing import List, Any
import warnings

[docs]class Callback:
    """
    Abstract base class used to build new callbacks.
    """

    def __init__(self):
        pass

[docs]    def set_params(self, params):
        self.params = params


[docs]    def set_trainer(self, model):
        self.trainer = model


[docs]    def on_epoch_begin(self, epoch, logs=None):
        pass


[docs]    def on_epoch_end(self, epoch, logs=None):
        pass


[docs]    def on_batch_begin(self, batch, logs=None):
        pass


[docs]    def on_batch_end(self, batch, logs=None):
        pass


[docs]    def on_train_begin(self, logs=None):
        pass


[docs]    def on_train_end(self, logs=None):
        pass


[docs]@dataclass
class CallbackContainer:
    """
    Container holding a list of callbacks.
    """

    callbacks: List[Callback] = field(default_factory=list)

[docs]    def append(self, callback):
        self.callbacks.append(callback)


[docs]    def set_params(self, params):
        for callback in self.callbacks:
            callback.set_params(params)


[docs]    def set_trainer(self, trainer):
        self.trainer = trainer
        for callback in self.callbacks:
            callback.set_trainer(trainer)


[docs]    def on_epoch_begin(self, epoch, logs=None):
        logs = logs or {}
        for callback in self.callbacks:
            callback.on_epoch_begin(epoch, logs)


[docs]    def on_epoch_end(self, epoch, logs=None):
        logs = logs or {}
        for callback in self.callbacks:
            callback.on_epoch_end(epoch, logs)


[docs]    def on_batch_begin(self, batch, logs=None):
        logs = logs or {}
        for callback in self.callbacks:
            callback.on_batch_begin(batch, logs)


[docs]    def on_batch_end(self, batch, logs=None):
        logs = logs or {}
        for callback in self.callbacks:
            callback.on_batch_end(batch, logs)


[docs]    def on_train_begin(self, logs=None):
        logs = logs or {}
        logs["start_time"] = time.time()
        for callback in self.callbacks:
            callback.on_train_begin(logs)


[docs]    def on_train_end(self, logs=None):
        logs = logs or {}
        for callback in self.callbacks:
            callback.on_train_end(logs)


[docs]@dataclass
class EarlyStopping(Callback):
    """EarlyStopping callback to exit the training loop if early_stopping_metric
    does not improve by a certain amount for a certain
    number of epochs.

    Parameters
    ---------
    early_stopping_metric : str
        Early stopping metric name
    is_maximize : bool
        Whether to maximize or not early_stopping_metric
    tol : float
        minimum change in monitored value to qualify as improvement.
        This number should be positive.
    patience : integer
        number of epochs to wait for improvement before terminating.
        the counter be reset after each improvement

    """

    early_stopping_metric: str
    is_maximize: bool
    tol: float = 0.0
    patience: int = 5

    def __post_init__(self):
        self.best_epoch = 0
        self.stopped_epoch = 0
        self.wait = 0
        self.best_weights = None
        self.best_loss = np.inf
        if self.is_maximize:
            self.best_loss = -self.best_loss
        super().__init__()

[docs]    def on_epoch_end(self, epoch, logs=None):
        current_loss = logs.get(self.early_stopping_metric)
        if current_loss is None:
            return

        loss_change = current_loss - self.best_loss
        max_improved = self.is_maximize and loss_change > self.tol
        min_improved = (not self.is_maximize) and (-loss_change > self.tol)
        if max_improved or min_improved:
            self.best_loss = current_loss
            self.best_epoch = epoch
            self.wait = 1
            self.best_weights = copy.deepcopy(self.trainer.network.state_dict())
        else:
            if self.wait >= self.patience:
                self.stopped_epoch = epoch
                self.trainer._stop_training = True
            self.wait += 1


[docs]    def on_train_end(self, logs=None):
        self.trainer.best_epoch = self.best_epoch
        self.trainer.best_cost = self.best_loss

        if self.best_weights is not None:
            self.trainer.network.load_state_dict(self.best_weights)

        if self.stopped_epoch > 0:
            msg = f"\nEarly stopping occurred at epoch {self.stopped_epoch}"
            msg += (
                f" with best_epoch = {self.best_epoch} and "
                + f"best_{self.early_stopping_metric} = {round(self.best_loss, 5)}"
            )
            print(msg)
        else:
            msg = (
                f"Stop training because you reached max_epochs = {self.trainer.max_epochs}"
                + f" with best_epoch = {self.best_epoch} and "
                + f"best_{self.early_stopping_metric} = {round(self.best_loss, 5)}"
            )
            print(msg)
        wrn_msg = "Best weights from best epoch are automatically used!"
        warnings.warn(wrn_msg)


[docs]@dataclass
class History(Callback):
    """Callback that records events into a `History` object.
    This callback is automatically applied to
    every SuperModule.

    Parameters
    ---------
    trainer : DeepRecoModel
        Model class to train
    verbose : int
        Print results every verbose iteration

    """

    trainer: Any
    verbose: int = 1

    def __post_init__(self):
        super().__init__()
        self.samples_seen = 0.0
        self.total_time = 0.0

[docs]    def on_train_begin(self, logs=None):
        self.history = {"loss": []}
        self.history.update({"lr": []})
        self.history.update({name: [] for name in self.trainer._metrics_names})
        self.start_time = logs["start_time"]
        self.epoch_loss = 0.0


[docs]    def on_epoch_begin(self, epoch, logs=None):
        self.epoch_metrics = {"loss": 0.0}
        self.samples_seen = 0.0


[docs]    def on_epoch_end(self, epoch, logs=None):
        self.epoch_metrics["loss"] = self.epoch_loss
        for metric_name, metric_value in self.epoch_metrics.items():
            self.history[metric_name].append(metric_value)
        if self.verbose == 0:
            return
        if epoch % self.verbose != 0:
            return
        msg = f"epoch {epoch:<3}"
        for metric_name, metric_value in self.epoch_metrics.items():
            if metric_name != "lr":
                msg += f"| {metric_name:<3}: {np.round(metric_value, 5):<8}"
        self.total_time = int(time.time() - self.start_time)
        msg += f"|  {str(datetime.timedelta(seconds=self.total_time)) + 's':<6}"
        print(msg)


[docs]    def on_batch_end(self, batch, logs=None):
        batch_size = logs["batch_size"]
        self.epoch_loss = (
            self.samples_seen * self.epoch_loss + batch_size * logs["loss"]
        ) / (self.samples_seen + batch_size)
        self.samples_seen += batch_size

def __getitem__(self, name):
        return self.history[name]

    def __repr__(self):
        return str(self.history)

    def __str__(self):
        return str(self.history)


[docs]@dataclass
class LRSchedulerCallback(Callback):
    """Wrapper for most torch scheduler functions.

    Parameters
    ---------
    scheduler_fn : torch.optim.lr_scheduler
        Torch scheduling class
    scheduler_params : dict
        Dictionnary containing all parameters for the scheduler_fn
    is_batch_level : bool (default = False)
        If set to False : lr updates will happen at every epoch
        If set to True : lr updates happen at every batch
        Set this to True for OneCycleLR for example
    """

    scheduler_fn: Any
    optimizer: Any
    scheduler_params: dict
    early_stopping_metric: str
    is_batch_level: bool = False

    def __post_init__(
        self,
    ):
        self.is_metric_related = hasattr(self.scheduler_fn, "is_better")
        self.scheduler = self.scheduler_fn(self.optimizer, **self.scheduler_params)
        super().__init__()

[docs]    def on_batch_end(self, batch, logs=None):
        if self.is_batch_level:
            self.scheduler.step()
        else:
            pass


[docs]    def on_epoch_end(self, epoch, logs=None):
        current_loss = logs.get(self.early_stopping_metric)
        if current_loss is None:
            return
        if self.is_batch_level:
            pass
        else:
            if self.is_metric_related:
                self.scheduler.step(current_loss)
            else:
                self.scheduler.step()
```

---

© Copyright 2019, Dreamquark

Built with Sphinx using a
theme
provided by Read the Docs.
