# Supplementary material for: Development of a deep learning model for predicting recurrence of hepatocellular carcinoma after liver transplantation
Source: Front Med (Lausanne). 2024 Jun 11;11:1373005. doi: 10.3389/fmed.2024.1373005 (PMC11196752; doi:10.3389/fmed.2024.1373005)
Supplement: Supplementary file 1 [file Data_Sheet_1.ZIP › Raw data/source data and codes/codes/tabnet/docs/_modules/pytorch_tabnet/metrics.html]

pytorch\_tabnet.metrics — pytorch\_tabnet documentation


pytorch\_tabnet

Contents:

- README
- TabNet : Attentive Interpretable Tabular Learning
- Installation
- What is new ?
- Contributing
- What problems does pytorch-tabnet handle?
- How to use it?
- Semi-supervised pre-training
- Data augmentation on the fly
- Easy saving and loading
- Useful links
- pytorch\_tabnet package

pytorch\_tabnet

- »
- Module code »
- pytorch\_tabnet.metrics

---

# Source code for pytorch\_tabnet.metrics

```
from dataclasses import dataclass
from typing import List
import numpy as np
from sklearn.metrics import (
    roc_auc_score,
    mean_squared_error,
    mean_absolute_error,
    accuracy_score,
    log_loss,
    balanced_accuracy_score,
    mean_squared_log_error,
)
import torch

[docs]def UnsupervisedLoss(y_pred, embedded_x, obf_vars, eps=1e-9):
    """
    Implements unsupervised loss function.
    This differs from orginal paper as it's scaled to be batch size independent
    and number of features reconstructed independent (by taking the mean)

    Parameters
    ----------
    y_pred : torch.Tensor or np.array
        Reconstructed prediction (with embeddings)
    embedded_x : torch.Tensor
        Original input embedded by network
    obf_vars : torch.Tensor
        Binary mask for obfuscated variables.
        1 means the variable was obfuscated so reconstruction is based on this.
    eps : float
        A small floating point to avoid ZeroDivisionError
        This can happen in degenerated case when a feature has only one value

    Returns
    -------
    loss : torch float
        Unsupervised loss, average value over batch samples.
    """
    errors = y_pred - embedded_x
    reconstruction_errors = torch.mul(errors, obf_vars) ** 2
    batch_means = torch.mean(embedded_x, dim=0)
    batch_means[batch_means == 0] = 1

    batch_stds = torch.std(embedded_x, dim=0) ** 2
    batch_stds[batch_stds == 0] = batch_means[batch_stds == 0]
    features_loss = torch.matmul(reconstruction_errors, 1 / batch_stds)
    # compute the number of obfuscated variables to reconstruct
    nb_reconstructed_variables = torch.sum(obf_vars, dim=1)
    # take the mean of the reconstructed variable errors
    features_loss = features_loss / (nb_reconstructed_variables + eps)
    # here we take the mean per batch, contrary to the paper
    loss = torch.mean(features_loss)
    return loss


[docs]def UnsupervisedLossNumpy(y_pred, embedded_x, obf_vars, eps=1e-9):
    errors = y_pred - embedded_x
    reconstruction_errors = np.multiply(errors, obf_vars) ** 2
    batch_means = np.mean(embedded_x, axis=0)
    batch_means = np.where(batch_means == 0, 1, batch_means)

    batch_stds = np.std(embedded_x, axis=0, ddof=1) ** 2
    batch_stds = np.where(batch_stds == 0, batch_means, batch_stds)
    features_loss = np.matmul(reconstruction_errors, 1 / batch_stds)
    # compute the number of obfuscated variables to reconstruct
    nb_reconstructed_variables = np.sum(obf_vars, axis=1)
    # take the mean of the reconstructed variable errors
    features_loss = features_loss / (nb_reconstructed_variables + eps)
    # here we take the mean per batch, contrary to the paper
    loss = np.mean(features_loss)
    return loss


[docs]@dataclass
class UnsupMetricContainer:
    """Container holding a list of metrics.

    Parameters
    ----------
    y_pred : torch.Tensor or np.array
        Reconstructed prediction (with embeddings)
    embedded_x : torch.Tensor
        Original input embedded by network
    obf_vars : torch.Tensor
        Binary mask for obfuscated variables.
        1 means the variables was obfuscated so reconstruction is based on this.

    """

    metric_names: List[str]
    prefix: str = ""

    def __post_init__(self):
        self.metrics = Metric.get_metrics_by_names(self.metric_names)
        self.names = [self.prefix + name for name in self.metric_names]

    def __call__(self, y_pred, embedded_x, obf_vars):
        """Compute all metrics and store into a dict.

        Parameters
        ----------
        y_true : np.ndarray
            Target matrix or vector
        y_pred : np.ndarray
            Score matrix or vector

        Returns
        -------
        dict
            Dict of metrics ({metric_name: metric_value}).

        """
        logs = {}
        for metric in self.metrics:
            res = metric(y_pred, embedded_x, obf_vars)
            logs[self.prefix + metric._name] = res
        return logs


[docs]@dataclass
class MetricContainer:
    """Container holding a list of metrics.

    Parameters
    ----------
    metric_names : list of str
        List of metric names.
    prefix : str
        Prefix of metric names.

    """

    metric_names: List[str]
    prefix: str = ""

    def __post_init__(self):
        self.metrics = Metric.get_metrics_by_names(self.metric_names)
        self.names = [self.prefix + name for name in self.metric_names]

    def __call__(self, y_true, y_pred):
        """Compute all metrics and store into a dict.

        Parameters
        ----------
        y_true : np.ndarray
            Target matrix or vector
        y_pred : np.ndarray
            Score matrix or vector

        Returns
        -------
        dict
            Dict of metrics ({metric_name: metric_value}).

        """
        logs = {}
        for metric in self.metrics:
            if isinstance(y_pred, list):
                res = np.mean(
                    [metric(y_true[:, i], y_pred[i]) for i in range(len(y_pred))]
                )
            else:
                res = metric(y_true, y_pred)
            logs[self.prefix + metric._name] = res
        return logs


[docs]class Metric:
    def __call__(self, y_true, y_pred):
        raise NotImplementedError("Custom Metrics must implement this function")

[docs]    @classmethod
    def get_metrics_by_names(cls, names):
        """Get list of metric classes.

        Parameters
        ----------
        cls : Metric
            Metric class.
        names : list
            List of metric names.

        Returns
        -------
        metrics : list
            List of metric classes.

        """
        available_metrics = cls.__subclasses__()
        available_names = [metric()._name for metric in available_metrics]
        metrics = []
        for name in names:
            assert (
                name in available_names
            ), f"{name} is not available, choose in {available_names}"
            idx = available_names.index(name)
            metric = available_metrics[idx]()
            metrics.append(metric)
        return metrics


[docs]class AUC(Metric):
    """
    AUC.
    """

    def __init__(self):
        self._name = "auc"
        self._maximize = True

    def __call__(self, y_true, y_score):
        """
        Compute AUC of predictions.

        Parameters
        ----------
        y_true : np.ndarray
            Target matrix or vector
        y_score : np.ndarray
            Score matrix or vector

        Returns
        -------
        float
            AUC of predictions vs targets.
        """
        return roc_auc_score(y_true, y_score[:, 1])


[docs]class Accuracy(Metric):
    """
    Accuracy.
    """

    def __init__(self):
        self._name = "accuracy"
        self._maximize = True

    def __call__(self, y_true, y_score):
        """
        Compute Accuracy of predictions.

        Parameters
        ----------
        y_true: np.ndarray
            Target matrix or vector
        y_score: np.ndarray
            Score matrix or vector

        Returns
        -------
        float
            Accuracy of predictions vs targets.
        """
        y_pred = np.argmax(y_score, axis=1)
        return accuracy_score(y_true, y_pred)


[docs]class BalancedAccuracy(Metric):
    """
    Balanced Accuracy.
    """

    def __init__(self):
        self._name = "balanced_accuracy"
        self._maximize = True

    def __call__(self, y_true, y_score):
        """
        Compute Accuracy of predictions.

        Parameters
        ----------
        y_true : np.ndarray
            Target matrix or vector
        y_score : np.ndarray
            Score matrix or vector

        Returns
        -------
        float
            Accuracy of predictions vs targets.
        """
        y_pred = np.argmax(y_score, axis=1)
        return balanced_accuracy_score(y_true, y_pred)


[docs]class LogLoss(Metric):
    """
    LogLoss.
    """

    def __init__(self):
        self._name = "logloss"
        self._maximize = False

    def __call__(self, y_true, y_score):
        """
        Compute LogLoss of predictions.

        Parameters
        ----------
        y_true : np.ndarray
            Target matrix or vector
        y_score : np.ndarray
            Score matrix or vector

        Returns
        -------
        float
            LogLoss of predictions vs targets.
        """
        return log_loss(y_true, y_score)


[docs]class MAE(Metric):
    """
    Mean Absolute Error.
    """

    def __init__(self):
        self._name = "mae"
        self._maximize = False

    def __call__(self, y_true, y_score):
        """
        Compute MAE (Mean Absolute Error) of predictions.

        Parameters
        ----------
        y_true : np.ndarray
            Target matrix or vector
        y_score : np.ndarray
            Score matrix or vector

        Returns
        -------
        float
            MAE of predictions vs targets.
        """
        return mean_absolute_error(y_true, y_score)


[docs]class MSE(Metric):
    """
    Mean Squared Error.
    """

    def __init__(self):
        self._name = "mse"
        self._maximize = False

    def __call__(self, y_true, y_score):
        """
        Compute MSE (Mean Squared Error) of predictions.

        Parameters
        ----------
        y_true : np.ndarray
            Target matrix or vector
        y_score : np.ndarray
            Score matrix or vector

        Returns
        -------
        float
            MSE of predictions vs targets.
        """
        return mean_squared_error(y_true, y_score)


[docs]class RMSLE(Metric):
    """
    Root Mean squared logarithmic error regression loss.
    Scikit-implementation:
    https://scikit-learn.org/stable/modules/generated/sklearn.metrics.mean_squared_log_error.html
    Note: In order to avoid error, negative predictions are clipped to 0.
    This means that you should clip negative predictions manually after calling predict.
    """

    def __init__(self):
        self._name = "rmsle"
        self._maximize = False

    def __call__(self, y_true, y_score):
        """
        Compute RMSLE of predictions.

        Parameters
        ----------
        y_true : np.ndarray
            Target matrix or vector
        y_score : np.ndarray
            Score matrix or vector

        Returns
        -------
        float
            RMSLE of predictions vs targets.
        """
        y_score = np.clip(y_score, a_min=0, a_max=None)
        return np.sqrt(mean_squared_log_error(y_true, y_score))


[docs]class UnsupervisedMetric(Metric):
    """
    Unsupervised metric
    """

    def __init__(self):
        self._name = "unsup_loss"
        self._maximize = False

    def __call__(self, y_pred, embedded_x, obf_vars):
        """
        Compute MSE (Mean Squared Error) of predictions.

        Parameters
        ----------
        y_pred : torch.Tensor or np.array
            Reconstructed prediction (with embeddings)
        embedded_x : torch.Tensor
            Original input embedded by network
        obf_vars : torch.Tensor
            Binary mask for obfuscated variables.
            1 means the variables was obfuscated so reconstruction is based on this.

        Returns
        -------
        float
            MSE of predictions vs targets.
        """
        loss = UnsupervisedLoss(y_pred, embedded_x, obf_vars)
        return loss.item()


[docs]class UnsupervisedNumpyMetric(Metric):
    """
    Unsupervised metric
    """

    def __init__(self):
        self._name = "unsup_loss_numpy"
        self._maximize = False

    def __call__(self, y_pred, embedded_x, obf_vars):
        """
        Compute MSE (Mean Squared Error) of predictions.

        Parameters
        ----------
        y_pred : torch.Tensor or np.array
            Reconstructed prediction (with embeddings)
        embedded_x : torch.Tensor
            Original input embedded by network
        obf_vars : torch.Tensor
            Binary mask for obfuscated variables.
            1 means the variables was obfuscated so reconstruction is based on this.

        Returns
        -------
        float
            MSE of predictions vs targets.
        """
        return UnsupervisedLossNumpy(
            y_pred,
            embedded_x,
            obf_vars
        )


[docs]class RMSE(Metric):
    """
    Root Mean Squared Error.
    """

    def __init__(self):
        self._name = "rmse"
        self._maximize = False

    def __call__(self, y_true, y_score):
        """
        Compute RMSE (Root Mean Squared Error) of predictions.

        Parameters
        ----------
        y_true : np.ndarray
            Target matrix or vector
        y_score : np.ndarray
            Score matrix or vector

        Returns
        -------
        float
            RMSE of predictions vs targets.
        """
        return np.sqrt(mean_squared_error(y_true, y_score))


[docs]def check_metrics(metrics):
    """Check if custom metrics are provided.

    Parameters
    ----------
    metrics : list of str or classes
        List with built-in metrics (str) or custom metrics (classes).

    Returns
    -------
    val_metrics : list of str
        List of metric names.

    """
    val_metrics = []
    for metric in metrics:
        if isinstance(metric, str):
            val_metrics.append(metric)
        elif issubclass(metric, Metric):
            val_metrics.append(metric()._name)
        else:
            raise TypeError("You need to provide a valid metric format")
    return val_metrics
```

---

© Copyright 2019, Dreamquark

Built with Sphinx using a
theme
provided by Read the Docs.
