# Supplementary material for: Development of a deep learning model for predicting recurrence of hepatocellular carcinoma after liver transplantation
Source: Front Med (Lausanne). 2024 Jun 11;11:1373005. doi: 10.3389/fmed.2024.1373005 (PMC11196752; doi:10.3389/fmed.2024.1373005)
Supplement: Supplementary file 1 [file Data_Sheet_1.ZIP › Raw data/source data and codes/codes/tabnet/docs/_modules/pytorch_tabnet/multiclass_utils.html]

pytorch\_tabnet.multiclass\_utils — pytorch\_tabnet documentation


pytorch\_tabnet

Contents:

- README
- TabNet : Attentive Interpretable Tabular Learning
- Installation
- What is new ?
- Contributing
- What problems does pytorch-tabnet handle?
- How to use it?
- Semi-supervised pre-training
- Data augmentation on the fly
- Easy saving and loading
- Useful links
- pytorch\_tabnet package

pytorch\_tabnet

- »
- Module code »
- pytorch\_tabnet.multiclass\_utils

---

# Source code for pytorch\_tabnet.multiclass\_utils

```
# Author: Arnaud Joly, Joel Nothman, Hamzeh Alsalhi
#
# License: BSD 3 clause
"""
Multi-class / multi-label utility function
==========================================

"""
from collections.abc import Sequence
from itertools import chain

from scipy.sparse import issparse
from scipy.sparse.base import spmatrix
from scipy.sparse import dok_matrix
from scipy.sparse import lil_matrix
import scipy.sparse as sp

import numpy as np
import pandas as pd


def _assert_all_finite(X, allow_nan=False):
    """Like assert_all_finite, but only for ndarray."""

    X = np.asanyarray(X)
    # First try an O(n) time, O(1) space solution for the common case that
    # everything is finite; fall back to O(n) space np.isfinite to prevent
    # false positives from overflow in sum method. The sum is also calculated
    # safely to reduce dtype induced overflows.
    is_float = X.dtype.kind in "fc"
    if is_float and (np.isfinite(np.sum(X))):
        pass
    elif is_float:
        msg_err = "Input contains {} or a value too large for {!r}."
        if (
            allow_nan
            and np.isinf(X).any()
            or not allow_nan
            and not np.isfinite(X).all()
        ):
            type_err = "infinity" if allow_nan else "NaN, infinity"
            raise ValueError(msg_err.format(type_err, X.dtype))
    # for object dtype data, we only check for NaNs (GH-13254)
    elif X.dtype == np.dtype("object") and not allow_nan:
        if np.isnan(X).any():
            raise ValueError("Input contains NaN")

[docs]def assert_all_finite(X, allow_nan=False):
    """Throw a ValueError if X contains NaN or infinity.

    Parameters
    ----------
    X : array or sparse matrix
    allow_nan : bool
    """
    _assert_all_finite(X.data if sp.issparse(X) else X, allow_nan)

def _unique_multiclass(y):
    if hasattr(y, "__array__"):
        return np.unique(np.asarray(y))
    else:
        return set(y)


def _unique_indicator(y):
    """
    Not implemented
    """
    raise IndexError(
        f"""Given labels are of size {y.shape} while they should be (n_samples,) \n"""
        + """If attempting multilabel classification, try using TabNetMultiTaskClassification """
        + """or TabNetRegressor"""
    )


_FN_UNIQUE_LABELS = {
    "binary": _unique_multiclass,
    "multiclass": _unique_multiclass,
    "multilabel-indicator": _unique_indicator,
}

[docs]def unique_labels(*ys):
    """Extract an ordered array of unique labels

    We don't allow:
        - mix of multilabel and multiclass (single label) targets
        - mix of label indicator matrix and anything else,
          because there are no explicit labels)
        - mix of label indicator matrices of different sizes
        - mix of string and integer labels

    At the moment, we also don't allow "multiclass-multioutput" input type.

    Parameters
    ----------
    *ys : array-likes

    Returns
    -------
    out : numpy array of shape [n_unique_labels]
        An ordered array of unique labels.

    Examples
    --------
    >>> from sklearn.utils.multiclass import unique_labels
    >>> unique_labels([3, 5, 5, 5, 7, 7])
    array([3, 5, 7])
    >>> unique_labels([1, 2, 3, 4], [2, 2, 3, 4])
    array([1, 2, 3, 4])
    >>> unique_labels([1, 2, 10], [5, 11])
    array([ 1,  2,  5, 10, 11])
    """
    if not ys:
        raise ValueError("No argument has been passed.")
    # Check that we don't mix label format

    ys_types = set(type_of_target(x) for x in ys)
    if ys_types == {"binary", "multiclass"}:
        ys_types = {"multiclass"}

    if len(ys_types) > 1:
        raise ValueError("Mix type of y not allowed, got types %s" % ys_types)

    label_type = ys_types.pop()

    # Get the unique set of labels
    _unique_labels = _FN_UNIQUE_LABELS.get(label_type, None)
    if not _unique_labels:
        raise ValueError("Unknown label type: %s" % repr(ys))

    ys_labels = set(chain.from_iterable(_unique_labels(y) for y in ys))

    # Check that we don't mix string type with number type
    if len(set(isinstance(label, str) for label in ys_labels)) > 1:
        raise ValueError("Mix of label input types (string and number)")

    return np.array(sorted(ys_labels))

def _is_integral_float(y):
    return y.dtype.kind == "f" and np.all(y.astype(int) == y)

[docs]def is_multilabel(y):
    """Check if ``y`` is in a multilabel format.

    Parameters
    ----------
    y : numpy array of shape [n_samples]
        Target values.

    Returns
    -------
    out : bool
        Return ``True``, if ``y`` is in a multilabel format, else ```False``.

    Examples
    --------
    >>> import numpy as np
    >>> from sklearn.utils.multiclass import is_multilabel
    >>> is_multilabel([0, 1, 0, 1])
    False
    >>> is_multilabel([[1], [0, 2], []])
    False
    >>> is_multilabel(np.array([[1, 0], [0, 0]]))
    True
    >>> is_multilabel(np.array([[1], [0], [0]]))
    False
    >>> is_multilabel(np.array([[1, 0, 0]]))
    True
    """
    if hasattr(y, "__array__"):
        y = np.asarray(y)
    if not (hasattr(y, "shape") and y.ndim == 2 and y.shape[1] > 1):
        return False

    if issparse(y):
        if isinstance(y, (dok_matrix, lil_matrix)):
            y = y.tocsr()
        return (
            len(y.data) == 0
            or np.unique(y.data).size == 1
            and (
                y.dtype.kind in "biu"
                or _is_integral_float(np.unique(y.data))  # bool, int, uint
            )
        )
    else:
        labels = np.unique(y)

        return len(labels) < 3 and (
            y.dtype.kind in "biu" or _is_integral_float(labels)  # bool, int, uint
        )


[docs]def check_classification_targets(y):
    """Ensure that target y is of a non-regression type.

    Only the following target types (as defined in type_of_target) are allowed:
        'binary', 'multiclass', 'multiclass-multioutput',
        'multilabel-indicator', 'multilabel-sequences'

    Parameters
    ----------
    y : array-like
    """
    y_type = type_of_target(y)
    if y_type not in [
        "binary",
        "multiclass",
        "multiclass-multioutput",
        "multilabel-indicator",
        "multilabel-sequences",
    ]:
        raise ValueError("Unknown label type: %r" % y_type)


[docs]def type_of_target(y):
    """Determine the type of data indicated by the target.

    Note that this type is the most specific type that can be inferred.
    For example:

        * ``binary`` is more specific but compatible with ``multiclass``.
        * ``multiclass`` of integers is more specific but compatible with
          ``continuous``.
        * ``multilabel-indicator`` is more specific but compatible with
          ``multiclass-multioutput``.

    Parameters
    ----------
    y : array-like

    Returns
    -------
    target_type : string
        One of:

        * 'continuous': `y` is an array-like of floats that are not all
          integers, and is 1d or a column vector.
        * 'continuous-multioutput': `y` is a 2d array of floats that are
          not all integers, and both dimensions are of size > 1.
        * 'binary': `y` contains <= 2 discrete values and is 1d or a column
          vector.
        * 'multiclass': `y` contains more than two discrete values, is not a
          sequence of sequences, and is 1d or a column vector.
        * 'multiclass-multioutput': `y` is a 2d array that contains more
          than two discrete values, is not a sequence of sequences, and both
          dimensions are of size > 1.
        * 'multilabel-indicator': `y` is a label indicator matrix, an array
          of two dimensions with at least two columns, and at most 2 unique
          values.
        * 'unknown': `y` is array-like but none of the above, such as a 3d
          array, sequence of sequences, or an array of non-sequence objects.

    Examples
    --------
    >>> import numpy as np
    >>> type_of_target([0.1, 0.6])
    'continuous'
    >>> type_of_target([1, -1, -1, 1])
    'binary'
    >>> type_of_target(['a', 'b', 'a'])
    'binary'
    >>> type_of_target([1.0, 2.0])
    'binary'
    >>> type_of_target([1, 0, 2])
    'multiclass'
    >>> type_of_target([1.0, 0.0, 3.0])
    'multiclass'
    >>> type_of_target(['a', 'b', 'c'])
    'multiclass'
    >>> type_of_target(np.array([[1, 2], [3, 1]]))
    'multiclass-multioutput'
    >>> type_of_target([[1, 2]])
    'multiclass-multioutput'
    >>> type_of_target(np.array([[1.5, 2.0], [3.0, 1.6]]))
    'continuous-multioutput'
    >>> type_of_target(np.array([[0, 1], [1, 1]]))
    'multilabel-indicator'
    """
    valid = (
        isinstance(y, (Sequence, spmatrix)) or hasattr(y, "__array__")
    ) and not isinstance(y, str)

    if not valid:
        raise ValueError(
            "Expected array-like (array or non-string sequence), " "got %r" % y
        )

    sparseseries = y.__class__.__name__ == "SparseSeries"
    if sparseseries:
        raise ValueError("y cannot be class 'SparseSeries'.")

    if is_multilabel(y):
        return "multilabel-indicator"

    try:
        y = np.asarray(y)
    except ValueError:
        # Known to fail in numpy 1.3 for array of arrays
        return "unknown"

    # The old sequence of sequences format
    try:
        if (
            not hasattr(y[0], "__array__")
            and isinstance(y[0], Sequence)
            and not isinstance(y[0], str)
        ):
            raise ValueError(
                "You appear to be using a legacy multi-label data"
                " representation. Sequence of sequences are no"
                " longer supported; use a binary array or sparse"
                " matrix instead - the MultiLabelBinarizer"
                " transformer can convert to this format."
            )
    except IndexError:
        pass

    # Invalid inputs
    if y.ndim > 2 or (y.dtype == object and len(y) and not isinstance(y.flat[0], str)):
        return "unknown"  # [[[1, 2]]] or [obj_1] and not ["label_1"]

    if y.ndim == 2 and y.shape[1] == 0:
        return "unknown"  # [[]]

    if y.ndim == 2 and y.shape[1] > 1:
        suffix = "-multioutput"  # [[1, 2], [1, 2]]
    else:
        suffix = ""  # [1, 2, 3] or [[1], [2], [3]]

    # check float and contains non-integer float values
    if y.dtype.kind == "f" and np.any(y != y.astype(int)):
        # [.1, .2, 3] or [[.1, .2, 3]] or [[1., .2]] and not [1., 2., 3.]
        _assert_all_finite(y)
        return "continuous" + suffix

    if (len(np.unique(y)) > 2) or (y.ndim >= 2 and len(y[0]) > 1):
        return "multiclass" + suffix  # [1, 2, 3] or [[1., 2., 3]] or [[1, 2]]
    else:
        return "binary"  # [1, 2] or [["a"], ["b"]]


[docs]def check_unique_type(y):
    target_types = pd.Series(y).map(type).unique()
    if len(target_types) != 1:
        raise TypeError(
            f"Values on the target must have the same type. Target has types {target_types}"
        )


[docs]def infer_output_dim(y_train):
    """
    Infer output_dim from targets

    Parameters
    ----------
    y_train : np.array
        Training targets

    Returns
    -------
    output_dim : int
        Number of classes for output
    train_labels : list
        Sorted list of initial classes
    """
    check_unique_type(y_train)
    train_labels = unique_labels(y_train)
    output_dim = len(train_labels)

    return output_dim, train_labels


[docs]def check_output_dim(labels, y):
    if y is not None:
        check_unique_type(y)
        valid_labels = unique_labels(y)
        if not set(valid_labels).issubset(set(labels)):
            raise ValueError(
                f"""Valid set -- {set(valid_labels)} --
                             contains unkown targets from training --
                             {set(labels)}"""
            )
    return


[docs]def infer_multitask_output(y_train):
    """
    Infer output_dim from targets
    This is for multiple tasks.

    Parameters
    ----------
    y_train : np.ndarray
        Training targets

    Returns
    -------
    tasks_dims : list
        Number of classes for output
    tasks_labels : list
        List of sorted list of initial classes
    """

    if len(y_train.shape) < 2:
        raise ValueError(
            "y_train should be of shape (n_examples, n_tasks)"
            + f"but got {y_train.shape}"
        )
    nb_tasks = y_train.shape[1]
    tasks_dims = []
    tasks_labels = []
    for task_idx in range(nb_tasks):
        try:
            output_dim, train_labels = infer_output_dim(y_train[:, task_idx])
            tasks_dims.append(output_dim)
            tasks_labels.append(train_labels)
        except ValueError as err:
            raise ValueError(f"""Error for task {task_idx} : {err}""")
    return tasks_dims, tasks_labels
```

---

© Copyright 2019, Dreamquark

Built with Sphinx using a
theme
provided by Read the Docs.
