# Supplementary material for: Development of a deep learning model for predicting recurrence of hepatocellular carcinoma after liver transplantation
Source: Front Med (Lausanne). 2024 Jun 11;11:1373005. doi: 10.3389/fmed.2024.1373005 (PMC11196752; doi:10.3389/fmed.2024.1373005)
Supplement: Supplementary file 1 [file Data_Sheet_1.ZIP › Raw data/source data and codes/codes/tabnet/docs/_modules/pytorch_tabnet/multitask.html]

pytorch\_tabnet.multitask — pytorch\_tabnet documentation


pytorch\_tabnet

Contents:

- README
- TabNet : Attentive Interpretable Tabular Learning
- Installation
- What is new ?
- Contributing
- What problems does pytorch-tabnet handle?
- How to use it?
- Semi-supervised pre-training
- Data augmentation on the fly
- Easy saving and loading
- Useful links
- pytorch\_tabnet package

pytorch\_tabnet

- »
- Module code »
- pytorch\_tabnet.multitask

---

# Source code for pytorch\_tabnet.multitask

```
import torch
import numpy as np
from scipy.special import softmax
from pytorch_tabnet.utils import SparsePredictDataset, PredictDataset, filter_weights
from pytorch_tabnet.abstract_model import TabModel
from pytorch_tabnet.multiclass_utils import infer_multitask_output, check_output_dim
from torch.utils.data import DataLoader
import scipy

[docs]class TabNetMultiTaskClassifier(TabModel):
    def __post_init__(self):
        super(TabNetMultiTaskClassifier, self).__post_init__()
        self._task = 'classification'
        self._default_loss = torch.nn.functional.cross_entropy
        self._default_metric = 'logloss'

[docs]    def prepare_target(self, y):
        y_mapped = y.copy()
        for task_idx in range(y.shape[1]):
            task_mapper = self.target_mapper[task_idx]
            y_mapped[:, task_idx] = np.vectorize(task_mapper.get)(y[:, task_idx])
        return y_mapped


[docs]    def compute_loss(self, y_pred, y_true):
        """
        Computes the loss according to network output and targets

        Parameters
        ----------
        y_pred : list of tensors
            Output of network
        y_true : LongTensor
            Targets label encoded

        Returns
        -------
        loss : torch.Tensor
            output of loss function(s)

        """
        loss = 0
        y_true = y_true.long()
        if isinstance(self.loss_fn, list):
            # if you specify a different loss for each task
            for task_loss, task_output, task_id in zip(
                self.loss_fn, y_pred, range(len(self.loss_fn))
            ):
                loss += task_loss(task_output, y_true[:, task_id])
        else:
            # same loss function is applied to all tasks
            for task_id, task_output in enumerate(y_pred):
                loss += self.loss_fn(task_output, y_true[:, task_id])

        loss /= len(y_pred)
        return loss


[docs]    def stack_batches(self, list_y_true, list_y_score):
        y_true = np.vstack(list_y_true)
        y_score = []
        for i in range(len(self.output_dim)):
            score = np.vstack([x[i] for x in list_y_score])
            score = softmax(score, axis=1)
            y_score.append(score)
        return y_true, y_score


[docs]    def update_fit_params(self, X_train, y_train, eval_set, weights):
        output_dim, train_labels = infer_multitask_output(y_train)
        for _, y in eval_set:
            for task_idx in range(y.shape[1]):
                check_output_dim(train_labels[task_idx], y[:, task_idx])
        self.output_dim = output_dim
        self.classes_ = train_labels
        self.target_mapper = [
            {class_label: index for index, class_label in enumerate(classes)}
            for classes in self.classes_
        ]
        self.preds_mapper = [
            {str(index): str(class_label) for index, class_label in enumerate(classes)}
            for classes in self.classes_
        ]
        self.updated_weights = weights
        filter_weights(self.updated_weights)


[docs]    def predict(self, X):
        """
        Make predictions on a batch (valid)

        Parameters
        ----------
        X : a :tensor: `torch.Tensor` or matrix: `scipy.sparse.csr_matrix`
            Input data

        Returns
        -------
        results : np.array
            Predictions of the most probable class
        """
        self.network.eval()

        if scipy.sparse.issparse(X):
            dataloader = DataLoader(
                SparsePredictDataset(X),
                batch_size=self.batch_size,
                shuffle=False,
            )
        else:
            dataloader = DataLoader(
                PredictDataset(X),
                batch_size=self.batch_size,
                shuffle=False,
            )

        results = {}
        for data in dataloader:
            data = data.to(self.device).float()
            output, _ = self.network(data)
            predictions = [
                torch.argmax(torch.nn.Softmax(dim=1)(task_output), dim=1)
                .cpu()
                .detach()
                .numpy()
                .reshape(-1)
                for task_output in output
            ]

            for task_idx in range(len(self.output_dim)):
                results[task_idx] = results.get(task_idx, []) + [predictions[task_idx]]
        # stack all task individually
        results = [np.hstack(task_res) for task_res in results.values()]
        # map all task individually
        results = [
            np.vectorize(self.preds_mapper[task_idx].get)(task_res.astype(str))
            for task_idx, task_res in enumerate(results)
        ]
        return results


[docs]    def predict_proba(self, X):
        """
        Make predictions for classification on a batch (valid)

        Parameters
        ----------
        X : a :tensor: `torch.Tensor` or matrix: `scipy.sparse.csr_matrix`
            Input data

        Returns
        -------
        res : list of np.ndarray

        """
        self.network.eval()

        if scipy.sparse.issparse(X):
            dataloader = DataLoader(
                SparsePredictDataset(X),
                batch_size=self.batch_size,
                shuffle=False,
            )
        else:
            dataloader = DataLoader(
                PredictDataset(X),
                batch_size=self.batch_size,
                shuffle=False,
            )

        results = {}
        for data in dataloader:
            data = data.to(self.device).float()
            output, _ = self.network(data)
            predictions = [
                torch.nn.Softmax(dim=1)(task_output).cpu().detach().numpy()
                for task_output in output
            ]
            for task_idx in range(len(self.output_dim)):
                results[task_idx] = results.get(task_idx, []) + [predictions[task_idx]]
        res = [np.vstack(task_res) for task_res in results.values()]
        return res
```

---

© Copyright 2019, Dreamquark

Built with Sphinx using a
theme
provided by Read the Docs.
