# Supplementary material for: Development of a deep learning model for predicting recurrence of hepatocellular carcinoma after liver transplantation
Source: Front Med (Lausanne). 2024 Jun 11;11:1373005. doi: 10.3389/fmed.2024.1373005 (PMC11196752; doi:10.3389/fmed.2024.1373005)
Supplement: Supplementary file 1 [file Data_Sheet_1.ZIP › Raw data/source data and codes/codes/tabnet/docs/_modules/pytorch_tabnet/pretraining.html]

pytorch\_tabnet.pretraining — pytorch\_tabnet documentation


pytorch\_tabnet

Contents:

- README
- TabNet : Attentive Interpretable Tabular Learning
- Installation
- What is new ?
- Contributing
- What problems does pytorch-tabnet handle?
- How to use it?
- Semi-supervised pre-training
- Data augmentation on the fly
- Easy saving and loading
- Useful links
- pytorch\_tabnet package

pytorch\_tabnet

- »
- Module code »
- pytorch\_tabnet.pretraining

---

# Source code for pytorch\_tabnet.pretraining

```
import torch
import numpy as np
from torch.utils.data import DataLoader
from pytorch_tabnet import tab_network
from pytorch_tabnet.utils import (
    create_explain_matrix,
    filter_weights,
    SparsePredictDataset,
    PredictDataset,
    check_input,
    create_group_matrix,
)
from torch.nn.utils import clip_grad_norm_
from pytorch_tabnet.pretraining_utils import (
    create_dataloaders,
    validate_eval_set,
)
from pytorch_tabnet.metrics import (
    UnsupMetricContainer,
    check_metrics,
    UnsupervisedLoss,
)
from pytorch_tabnet.abstract_model import TabModel
import scipy

[docs]class TabNetPretrainer(TabModel):
    def __post_init__(self):
        super(TabNetPretrainer, self).__post_init__()
        self._task = 'unsupervised'
        self._default_loss = UnsupervisedLoss
        self._default_metric = 'unsup_loss_numpy'

[docs]    def prepare_target(self, y):
        return y


[docs]    def compute_loss(self, output, embedded_x, obf_vars):
        return self.loss_fn(output, embedded_x, obf_vars)


[docs]    def update_fit_params(
        self,
        weights,
    ):
        self.updated_weights = weights
        filter_weights(self.updated_weights)
        self.preds_mapper = None


[docs]    def fit(
        self,
        X_train,
        eval_set=None,
        eval_name=None,
        loss_fn=None,
        pretraining_ratio=0.5,
        weights=0,
        max_epochs=100,
        patience=10,
        batch_size=1024,
        virtual_batch_size=128,
        num_workers=0,
        drop_last=True,
        callbacks=None,
        pin_memory=True,
        warm_start=False
    ):
        """Train a neural network stored in self.network
        Using train_dataloader for training data and
        valid_dataloader for validation.

        Parameters
        ----------
        X_train : np.ndarray
            Train set to reconstruct in self supervision
        eval_set : list of np.array
            List of evaluation set
            The last one is used for early stopping
        eval_name : list of str
            List of eval set names.
        eval_metric : list of str
            List of evaluation metrics.
            The last metric is used for early stopping.
        loss_fn : callable or None
            a PyTorch loss function
            should be left to None for self supervised and non experts
        pretraining_ratio : float
            Between 0 and 1, percentage of feature to mask for reconstruction
        weights : np.array
            Sampling weights for each example.
        max_epochs : int
            Maximum number of epochs during training
        patience : int
            Number of consecutive non improving epoch before early stopping
        batch_size : int
            Training batch size
        virtual_batch_size : int
            Batch size for Ghost Batch Normalization (virtual_batch_size < batch_size)
        num_workers : int
            Number of workers used in torch.utils.data.DataLoader
        drop_last : bool
            Whether to drop last batch during training
        callbacks : list of callback function
            List of custom callbacks
        pin_memory: bool
            Whether to set pin_memory to True or False during training
        """
        # update model name

        self.max_epochs = max_epochs
        self.patience = patience
        self.batch_size = batch_size
        self.virtual_batch_size = virtual_batch_size
        self.num_workers = num_workers
        self.drop_last = drop_last
        self.input_dim = X_train.shape[1]
        self._stop_training = False
        self.pin_memory = pin_memory and (self.device.type != "cpu")
        self.pretraining_ratio = pretraining_ratio
        eval_set = eval_set if eval_set else []

        if loss_fn is None:
            self.loss_fn = self._default_loss
        else:
            self.loss_fn = loss_fn

        check_input(X_train)

        self.update_fit_params(
            weights,
        )

        # Validate and reformat eval set depending on training data
        eval_names = validate_eval_set(eval_set, eval_name, X_train)
        train_dataloader, valid_dataloaders = self._construct_loaders(
            X_train, eval_set
        )

        if not hasattr(self, "network") or not warm_start:
            # model has never been fitted before of warm_start is False
            self._set_network()

        self._update_network_params()
        self._set_metrics(eval_names)
        self._set_optimizer()
        self._set_callbacks(callbacks)

        # Call method on_train_begin for all callbacks
        self._callback_container.on_train_begin()

        # Training loop over epochs
        for epoch_idx in range(self.max_epochs):

            # Call method on_epoch_begin for all callbacks
            self._callback_container.on_epoch_begin(epoch_idx)

            self._train_epoch(train_dataloader)

            # Apply predict epoch to all eval sets
            for eval_name, valid_dataloader in zip(eval_names, valid_dataloaders):
                self._predict_epoch(eval_name, valid_dataloader)

            # Call method on_epoch_end for all callbacks
            self._callback_container.on_epoch_end(
                epoch_idx, logs=self.history.epoch_metrics
            )

            if self._stop_training:
                break

        # Call method on_train_end for all callbacks
        self._callback_container.on_train_end()
        self.network.eval()

def _set_network(self):
        """Setup the network and explain matrix."""
        if not hasattr(self, 'pretraining_ratio'):
            self.pretraining_ratio = 0.5
        torch.manual_seed(self.seed)

        self.group_matrix = create_group_matrix(self.grouped_features, self.input_dim)

        self.network = tab_network.TabNetPretraining(
            self.input_dim,
            pretraining_ratio=self.pretraining_ratio,
            n_d=self.n_d,
            n_a=self.n_a,
            n_steps=self.n_steps,
            gamma=self.gamma,
            cat_idxs=self.cat_idxs,
            cat_dims=self.cat_dims,
            cat_emb_dim=self.cat_emb_dim,
            n_independent=self.n_independent,
            n_shared=self.n_shared,
            n_shared_decoder=self.n_shared_decoder,
            n_indep_decoder=self.n_indep_decoder,
            epsilon=self.epsilon,
            virtual_batch_size=self.virtual_batch_size,
            momentum=self.momentum,
            mask_type=self.mask_type,
            group_attention_matrix=self.group_matrix.to(self.device),
        ).to(self.device)

        self.reducing_matrix = create_explain_matrix(
            self.network.input_dim,
            self.network.cat_emb_dim,
            self.network.cat_idxs,
            self.network.post_embed_dim,
        )

    def _update_network_params(self):
        self.network.virtual_batch_size = self.virtual_batch_size
        self.network.pretraining_ratio = self.pretraining_ratio

    def _set_metrics(self, eval_names):
        """Set attributes relative to the metrics.

        Parameters
        ----------
        metrics : list of str
            List of eval metric names.
        eval_names : list of str
            List of eval set names.

        """
        metrics = [self._default_metric]

        metrics = check_metrics(metrics)
        # Set metric container for each sets
        self._metric_container_dict = {}
        for name in eval_names:
            self._metric_container_dict.update(
                {name: UnsupMetricContainer(metrics, prefix=f"{name}_")}
            )

        self._metrics = []
        self._metrics_names = []
        for _, metric_container in self._metric_container_dict.items():
            self._metrics.extend(metric_container.metrics)
            self._metrics_names.extend(metric_container.names)

        # Early stopping metric is the last eval metric
        self.early_stopping_metric = (
            self._metrics_names[-1] if len(self._metrics_names) > 0 else None
        )

    def _construct_loaders(self, X_train, eval_set):
        """Generate dataloaders for unsupervised train and eval set.

        Parameters
        ----------
        X_train : np.array
            Train set.
        eval_set : list of tuple
            List of eval tuple set (X, y).

        Returns
        -------
        train_dataloader : `torch.utils.data.Dataloader`
            Training dataloader.
        valid_dataloaders : list of `torch.utils.data.Dataloader`
            List of validation dataloaders.

        """
        train_dataloader, valid_dataloaders = create_dataloaders(
            X_train,
            eval_set,
            self.updated_weights,
            self.batch_size,
            self.num_workers,
            self.drop_last,
            self.pin_memory,
        )
        return train_dataloader, valid_dataloaders

    def _train_epoch(self, train_loader):
        """
        Trains one epoch of the network in self.network

        Parameters
        ----------
        train_loader : a :class: `torch.utils.data.Dataloader`
            DataLoader with train set
        """
        self.network.train()

        for batch_idx, X in enumerate(train_loader):
            self._callback_container.on_batch_begin(batch_idx)

            batch_logs = self._train_batch(X)

            self._callback_container.on_batch_end(batch_idx, batch_logs)

        epoch_logs = {"lr": self._optimizer.param_groups[-1]["lr"]}
        self.history.epoch_metrics.update(epoch_logs)

        return

    def _train_batch(self, X):
        """
        Trains one batch of data

        Parameters
        ----------
        X : torch.Tensor
            Train matrix

        Returns
        -------
        batch_outs : dict
            Dictionnary with "y": target and "score": prediction scores.
        batch_logs : dict
            Dictionnary with "batch_size" and "loss".
        """
        batch_logs = {"batch_size": X.shape[0]}

        X = X.to(self.device).float()

        for param in self.network.parameters():
            param.grad = None

        output, embedded_x, obf_vars = self.network(X)
        loss = self.compute_loss(output, embedded_x, obf_vars)

        # Perform backward pass and optimization
        loss.backward()
        if self.clip_value:
            clip_grad_norm_(self.network.parameters(), self.clip_value)
        self._optimizer.step()

        batch_logs["loss"] = loss.cpu().detach().numpy().item()

        return batch_logs

    def _predict_epoch(self, name, loader):
        """
        Predict an epoch and update metrics.

        Parameters
        ----------
        name : str
            Name of the validation set
        loader : torch.utils.data.Dataloader
                DataLoader with validation set
        """
        # Setting network on evaluation mode
        self.network.eval()

        list_output = []
        list_embedded_x = []
        list_obfuscation = []
        # Main loop
        for batch_idx, X in enumerate(loader):
            output, embedded_x, obf_vars = self._predict_batch(X)
            list_output.append(output.cpu().detach().numpy())
            list_embedded_x.append(embedded_x.cpu().detach().numpy())
            list_obfuscation.append(obf_vars.cpu().detach().numpy())

        output, embedded_x, obf_vars = self.stack_batches(list_output,
                                                          list_embedded_x,
                                                          list_obfuscation)

        metrics_logs = self._metric_container_dict[name](output, embedded_x, obf_vars)
        self.network.train()
        self.history.epoch_metrics.update(metrics_logs)
        return

    def _predict_batch(self, X):
        """
        Predict one batch of data.

        Parameters
        ----------
        X : torch.Tensor
            Owned products

        Returns
        -------
        np.array
            model scores
        """
        X = X.to(self.device).float()
        return self.network(X)

[docs]    def stack_batches(self, list_output, list_embedded_x, list_obfuscation):
        output = np.vstack(list_output)
        embedded_x = np.vstack(list_embedded_x)
        obf_vars = np.vstack(list_obfuscation)
        return output, embedded_x, obf_vars


[docs]    def predict(self, X):
        """
        Make predictions on a batch (valid)

        Parameters
        ----------
        X : a :tensor: `torch.Tensor` or matrix: `scipy.sparse.csr_matrix`
            Input data

        Returns
        -------
        predictions : np.array
            Predictions of the regression problem
        """
        self.network.eval()

        if scipy.sparse.issparse(X):
            dataloader = DataLoader(
                SparsePredictDataset(X),
                batch_size=self.batch_size,
                shuffle=False,
            )
        else:
            dataloader = DataLoader(
                PredictDataset(X),
                batch_size=self.batch_size,
                shuffle=False,
            )

        results = []
        embedded_res = []
        for batch_nb, data in enumerate(dataloader):
            data = data.to(self.device).float()
            output, embeded_x, _ = self.network(data)
            predictions = output.cpu().detach().numpy()
            results.append(predictions)
            embedded_res.append(embeded_x.cpu().detach().numpy())
        res_output = np.vstack(results)
        embedded_inputs = np.vstack(embedded_res)
        return res_output, embedded_inputs
```

---

© Copyright 2019, Dreamquark

Built with Sphinx using a
theme
provided by Read the Docs.
