# Supplementary material for: Development of a deep learning model for predicting recurrence of hepatocellular carcinoma after liver transplantation
Source: Front Med (Lausanne). 2024 Jun 11;11:1373005. doi: 10.3389/fmed.2024.1373005 (PMC11196752; doi:10.3389/fmed.2024.1373005)
Supplement: Supplementary file 1 [file Data_Sheet_1.ZIP › Raw data/source data and codes/codes/tabnet/docs/_modules/pytorch_tabnet/pretraining_utils.html]

pytorch\_tabnet.pretraining\_utils — pytorch\_tabnet documentation


pytorch\_tabnet

Contents:

- README
- TabNet : Attentive Interpretable Tabular Learning
- Installation
- What is new ?
- Contributing
- What problems does pytorch-tabnet handle?
- How to use it?
- Semi-supervised pre-training
- Data augmentation on the fly
- Easy saving and loading
- Useful links
- pytorch\_tabnet package

pytorch\_tabnet

- »
- Module code »
- pytorch\_tabnet.pretraining\_utils

---

# Source code for pytorch\_tabnet.pretraining\_utils

```
from torch.utils.data import DataLoader
from pytorch_tabnet.utils import (
    create_sampler,
    SparsePredictDataset,
    PredictDataset,
    check_input
)
import scipy

[docs]def create_dataloaders(
    X_train, eval_set, weights, batch_size, num_workers, drop_last, pin_memory
):
    """
    Create dataloaders with or without subsampling depending on weights and balanced.

    Parameters
    ----------
    X_train : np.ndarray or scipy.sparse.csr_matrix
        Training data
    eval_set : list of np.array (for Xs and ys) or scipy.sparse.csr_matrix (for Xs)
        List of eval sets
    weights : either 0, 1, dict or iterable
        if 0 (default) : no weights will be applied
        if 1 : classification only, will balanced class with inverse frequency
        if dict : keys are corresponding class values are sample weights
        if iterable : list or np array must be of length equal to nb elements
                      in the training set
    batch_size : int
        how many samples per batch to load
    num_workers : int
        how many subprocesses to use for data loading. 0 means that the data
        will be loaded in the main process
    drop_last : bool
        set to True to drop the last incomplete batch, if the dataset size is not
        divisible by the batch size. If False and the size of dataset is not
        divisible by the batch size, then the last batch will be smaller
    pin_memory : bool
        Whether to pin GPU memory during training

    Returns
    -------
    train_dataloader, valid_dataloader : torch.DataLoader, torch.DataLoader
        Training and validation dataloaders
    """
    need_shuffle, sampler = create_sampler(weights, X_train)

    if scipy.sparse.issparse(X_train):
        train_dataloader = DataLoader(
            SparsePredictDataset(X_train),
            batch_size=batch_size,
            sampler=sampler,
            shuffle=need_shuffle,
            num_workers=num_workers,
            drop_last=drop_last,
            pin_memory=pin_memory,
        )
    else:
        train_dataloader = DataLoader(
            PredictDataset(X_train),
            batch_size=batch_size,
            sampler=sampler,
            shuffle=need_shuffle,
            num_workers=num_workers,
            drop_last=drop_last,
            pin_memory=pin_memory,
        )

    valid_dataloaders = []
    for X in eval_set:
        if scipy.sparse.issparse(X):
            valid_dataloaders.append(
                DataLoader(
                    SparsePredictDataset(X),
                    batch_size=batch_size,
                    sampler=sampler,
                    shuffle=need_shuffle,
                    num_workers=num_workers,
                    drop_last=drop_last,
                    pin_memory=pin_memory,
                )
            )
        else:
            valid_dataloaders.append(
                DataLoader(
                    PredictDataset(X),
                    batch_size=batch_size,
                    sampler=sampler,
                    shuffle=need_shuffle,
                    num_workers=num_workers,
                    drop_last=drop_last,
                    pin_memory=pin_memory,
                )
            )

    return train_dataloader, valid_dataloaders


[docs]def validate_eval_set(eval_set, eval_name, X_train):
    """Check if the shapes of eval_set are compatible with X_train.

    Parameters
    ----------
    eval_set : List of numpy array
        The list evaluation set.
        The last one is used for early stopping
    X_train : np.ndarray
        Train owned products

    Returns
    -------
    eval_names : list of str
        Validated list of eval_names.

    """
    eval_names = eval_name or [f"val_{i}" for i in range(len(eval_set))]
    assert len(eval_set) == len(
        eval_names
    ), "eval_set and eval_name have not the same length"

    for set_nb, X in enumerate(eval_set):
        check_input(X)
        msg = (
            f"Number of columns is different between eval set {set_nb}"
            + f"({X.shape[1]}) and X_train ({X_train.shape[1]})"
        )
        assert X.shape[1] == X_train.shape[1], msg
    return eval_names
```

---

© Copyright 2019, Dreamquark

Built with Sphinx using a
theme
provided by Read the Docs.
