# Supplementary material for: Development of a deep learning model for predicting recurrence of hepatocellular carcinoma after liver transplantation
Source: Front Med (Lausanne). 2024 Jun 11;11:1373005. doi: 10.3389/fmed.2024.1373005 (PMC11196752; doi:10.3389/fmed.2024.1373005)
Supplement: Supplementary file 1 [file Data_Sheet_1.ZIP › Raw data/source data and codes/codes/tabnet/docs/_modules/pytorch_tabnet/sparsemax.html]

pytorch\_tabnet.sparsemax — pytorch\_tabnet documentation


pytorch\_tabnet

Contents:

- README
- TabNet : Attentive Interpretable Tabular Learning
- Installation
- What is new ?
- Contributing
- What problems does pytorch-tabnet handle?
- How to use it?
- Semi-supervised pre-training
- Data augmentation on the fly
- Easy saving and loading
- Useful links
- pytorch\_tabnet package

pytorch\_tabnet

- »
- Module code »
- pytorch\_tabnet.sparsemax

---

# Source code for pytorch\_tabnet.sparsemax

```
from torch import nn
from torch.autograd import Function
import torch.nn.functional as F

import torch

"""
Other possible implementations:
https://github.com/KrisKorrel/sparsemax-pytorch/blob/master/sparsemax.py
https://github.com/msobroza/SparsemaxPytorch/blob/master/mnist/sparsemax.py
https://github.com/vene/sparse-structured-attention/blob/master/pytorch/torchsparseattn/sparsemax.py
"""


# credits to Yandex https://github.com/Qwicen/node/blob/master/lib/nn_utils.py
def _make_ix_like(input, dim=0):
    d = input.size(dim)
    rho = torch.arange(1, d + 1, device=input.device, dtype=input.dtype)
    view = [1] * input.dim()
    view[0] = -1
    return rho.view(view).transpose(0, dim)

[docs]class SparsemaxFunction(Function):
    """
    An implementation of sparsemax (Martins & Astudillo, 2016). See
    :cite:`DBLP:journals/corr/MartinsA16` for detailed description.
    By Ben Peters and Vlad Niculae
    """

[docs]    @staticmethod
    def forward(ctx, input, dim=-1):
        """sparsemax: normalizing sparse transform (a la softmax)

        Parameters
        ----------
        ctx : torch.autograd.function._ContextMethodMixin
        input : torch.Tensor
            any shape
        dim : int
            dimension along which to apply sparsemax

        Returns
        -------
        output : torch.Tensor
            same shape as input

        """
        ctx.dim = dim
        max_val, _ = input.max(dim=dim, keepdim=True)
        input -= max_val  # same numerical stability trick as for softmax
        tau, supp_size = SparsemaxFunction._threshold_and_support(input, dim=dim)
        output = torch.clamp(input - tau, min=0)
        ctx.save_for_backward(supp_size, output)
        return output


[docs]    @staticmethod
    def backward(ctx, grad_output):
        supp_size, output = ctx.saved_tensors
        dim = ctx.dim
        grad_input = grad_output.clone()
        grad_input[output == 0] = 0

        v_hat = grad_input.sum(dim=dim) / supp_size.to(output.dtype).squeeze()
        v_hat = v_hat.unsqueeze(dim)
        grad_input = torch.where(output != 0, grad_input - v_hat, grad_input)
        return grad_input, None

@staticmethod
    def _threshold_and_support(input, dim=-1):
        """Sparsemax building block: compute the threshold

        Parameters
        ----------
        input: torch.Tensor
            any dimension
        dim : int
            dimension along which to apply the sparsemax

        Returns
        -------
        tau : torch.Tensor
            the threshold value
        support_size : torch.Tensor

        """

        input_srt, _ = torch.sort(input, descending=True, dim=dim)
        input_cumsum = input_srt.cumsum(dim) - 1
        rhos = _make_ix_like(input, dim)
        support = rhos * input_srt > input_cumsum

        support_size = support.sum(dim=dim).unsqueeze(dim)
        tau = input_cumsum.gather(dim, support_size - 1)
        tau /= support_size.to(input.dtype)
        return tau, support_size

sparsemax = SparsemaxFunction.apply

[docs]class Sparsemax(nn.Module):

    def __init__(self, dim=-1):
        self.dim = dim
        super(Sparsemax, self).__init__()

[docs]    def forward(self, input):
        return sparsemax(input, self.dim)


[docs]class Entmax15Function(Function):
    """
    An implementation of exact Entmax with alpha=1.5 (B. Peters, V. Niculae, A. Martins). See
    :cite:`https://arxiv.org/abs/1905.05702 for detailed description.
    Source: https://github.com/deep-spin/entmax
    """

[docs]    @staticmethod
    def forward(ctx, input, dim=-1):
        ctx.dim = dim

        max_val, _ = input.max(dim=dim, keepdim=True)
        input = input - max_val  # same numerical stability trick as for softmax
        input = input / 2  # divide by 2 to solve actual Entmax

        tau_star, _ = Entmax15Function._threshold_and_support(input, dim)
        output = torch.clamp(input - tau_star, min=0) ** 2
        ctx.save_for_backward(output)
        return output


[docs]    @staticmethod
    def backward(ctx, grad_output):
        Y, = ctx.saved_tensors
        gppr = Y.sqrt()  # = 1 / g'' (Y)
        dX = grad_output * gppr
        q = dX.sum(ctx.dim) / gppr.sum(ctx.dim)
        q = q.unsqueeze(ctx.dim)
        dX -= q * gppr
        return dX, None

@staticmethod
    def _threshold_and_support(input, dim=-1):
        Xsrt, _ = torch.sort(input, descending=True, dim=dim)

        rho = _make_ix_like(input, dim)
        mean = Xsrt.cumsum(dim) / rho
        mean_sq = (Xsrt ** 2).cumsum(dim) / rho
        ss = rho * (mean_sq - mean ** 2)
        delta = (1 - ss) / rho

        # NOTE this is not exactly the same as in reference algo
        # Fortunately it seems the clamped values never wrongly
        # get selected by tau <= sorted_z. Prove this!
        delta_nz = torch.clamp(delta, 0)
        tau = mean - torch.sqrt(delta_nz)

        support_size = (tau <= Xsrt).sum(dim).unsqueeze(dim)
        tau_star = tau.gather(dim, support_size - 1)
        return tau_star, support_size


[docs]class Entmoid15(Function):
    """ A highly optimized equivalent of lambda x: Entmax15([x, 0]) """

[docs]    @staticmethod
    def forward(ctx, input):
        output = Entmoid15._forward(input)
        ctx.save_for_backward(output)
        return output

@staticmethod
    def _forward(input):
        input, is_pos = abs(input), input >= 0
        tau = (input + torch.sqrt(F.relu(8 - input ** 2))) / 2
        tau.masked_fill_(tau <= input, 2.0)
        y_neg = 0.25 * F.relu(tau - input, inplace=True) ** 2
        return torch.where(is_pos, 1 - y_neg, y_neg)

[docs]    @staticmethod
    def backward(ctx, grad_output):
        return Entmoid15._backward(ctx.saved_tensors[0], grad_output)

@staticmethod
    def _backward(output, grad_output):
        gppr0, gppr1 = output.sqrt(), (1 - output).sqrt()
        grad_input = grad_output * gppr0
        q = grad_input / (gppr0 + gppr1)
        grad_input -= q * gppr0
        return grad_input

entmax15 = Entmax15Function.apply
entmoid15 = Entmoid15.apply

[docs]class Entmax15(nn.Module):

    def __init__(self, dim=-1):
        self.dim = dim
        super(Entmax15, self).__init__()

[docs]    def forward(self, input):
        return entmax15(input, self.dim)

# Credits were lost...
# def _make_ix_like(input, dim=0):
#     d = input.size(dim)
#     rho = torch.arange(1, d + 1, device=input.device, dtype=input.dtype)
#     view = [1] * input.dim()
#     view[0] = -1
#     return rho.view(view).transpose(0, dim)
#
#
# def _threshold_and_support(input, dim=0):
#     """Sparsemax building block: compute the threshold
#     Args:
#         input: any dimension
#         dim: dimension along which to apply the sparsemax
#     Returns:
#         the threshold value
#     """
#
#     input_srt, _ = torch.sort(input, descending=True, dim=dim)
#     input_cumsum = input_srt.cumsum(dim) - 1
#     rhos = _make_ix_like(input, dim)
#     support = rhos * input_srt > input_cumsum
#
#     support_size = support.sum(dim=dim).unsqueeze(dim)
#     tau = input_cumsum.gather(dim, support_size - 1)
#     tau /= support_size.to(input.dtype)
#     return tau, support_size
#
#
# class SparsemaxFunction(Function):
#
#     @staticmethod
#     def forward(ctx, input, dim=0):
#         """sparsemax: normalizing sparse transform (a la softmax)
#         Parameters:
#             input (Tensor): any shape
#             dim: dimension along which to apply sparsemax
#         Returns:
#             output (Tensor): same shape as input
#         """
#         ctx.dim = dim
#         max_val, _ = input.max(dim=dim, keepdim=True)
#         input -= max_val  # same numerical stability trick as for softmax
#         tau, supp_size = _threshold_and_support(input, dim=dim)
#         output = torch.clamp(input - tau, min=0)
#         ctx.save_for_backward(supp_size, output)
#         return output
#
#     @staticmethod
#     def backward(ctx, grad_output):
#         supp_size, output = ctx.saved_tensors
#         dim = ctx.dim
#         grad_input = grad_output.clone()
#         grad_input[output == 0] = 0
#
#         v_hat = grad_input.sum(dim=dim) / supp_size.to(output.dtype).squeeze()
#         v_hat = v_hat.unsqueeze(dim)
#         grad_input = torch.where(output != 0, grad_input - v_hat, grad_input)
#         return grad_input, None
#
#
# sparsemax = SparsemaxFunction.apply
#
#
# class Sparsemax(nn.Module):
#
#     def __init__(self, dim=0):
#         self.dim = dim
#         super(Sparsemax, self).__init__()
#
#     def forward(self, input):
#         return sparsemax(input, self.dim)
```

---

© Copyright 2019, Dreamquark

Built with Sphinx using a
theme
provided by Read the Docs.
