# Supplementary material for: Development of a deep learning model for predicting recurrence of hepatocellular carcinoma after liver transplantation
Source: Front Med (Lausanne). 2024 Jun 11;11:1373005. doi: 10.3389/fmed.2024.1373005 (PMC11196752; doi:10.3389/fmed.2024.1373005)
Supplement: Supplementary file 1 [file Data_Sheet_1.ZIP › Raw data/source data and codes/codes/tabnet/docs/_modules/pytorch_tabnet/tab_model.html]

pytorch\_tabnet.tab\_model — pytorch\_tabnet documentation


pytorch\_tabnet

Contents:

- README
- TabNet : Attentive Interpretable Tabular Learning
- Installation
- What is new ?
- Contributing
- What problems does pytorch-tabnet handle?
- How to use it?
- Semi-supervised pre-training
- Data augmentation on the fly
- Easy saving and loading
- Useful links
- pytorch\_tabnet package

pytorch\_tabnet

- »
- Module code »
- pytorch\_tabnet.tab\_model

---

# Source code for pytorch\_tabnet.tab\_model

```
import torch
import numpy as np
from scipy.special import softmax
from pytorch_tabnet.utils import SparsePredictDataset, PredictDataset, filter_weights
from pytorch_tabnet.abstract_model import TabModel
from pytorch_tabnet.multiclass_utils import infer_output_dim, check_output_dim
from torch.utils.data import DataLoader
import scipy

[docs]class TabNetClassifier(TabModel):
    def __post_init__(self):
        super(TabNetClassifier, self).__post_init__()
        self._task = 'classification'
        self._default_loss = torch.nn.functional.cross_entropy
        self._default_metric = 'accuracy'

[docs]    def weight_updater(self, weights):
        """
        Updates weights dictionary according to target_mapper.

        Parameters
        ----------
        weights : bool or dict
            Given weights for balancing training.

        Returns
        -------
        bool or dict
            Same bool if weights are bool, updated dict otherwise.

        """
        if isinstance(weights, int):
            return weights
        elif isinstance(weights, dict):
            return {self.target_mapper[key]: value for key, value in weights.items()}
        else:
            return weights


[docs]    def prepare_target(self, y):
        return np.vectorize(self.target_mapper.get)(y)


[docs]    def compute_loss(self, y_pred, y_true):
        return self.loss_fn(y_pred, y_true.long())


[docs]    def update_fit_params(
        self,
        X_train,
        y_train,
        eval_set,
        weights,
    ):
        output_dim, train_labels = infer_output_dim(y_train)
        for X, y in eval_set:
            check_output_dim(train_labels, y)
        self.output_dim = output_dim
        self._default_metric = ('auc' if self.output_dim == 2 else 'accuracy')
        self.classes_ = train_labels
        self.target_mapper = {
            class_label: index for index, class_label in enumerate(self.classes_)
        }
        self.preds_mapper = {
            str(index): class_label for index, class_label in enumerate(self.classes_)
        }
        self.updated_weights = self.weight_updater(weights)


[docs]    def stack_batches(self, list_y_true, list_y_score):
        y_true = np.hstack(list_y_true)
        y_score = np.vstack(list_y_score)
        y_score = softmax(y_score, axis=1)
        return y_true, y_score


[docs]    def predict_func(self, outputs):
        outputs = np.argmax(outputs, axis=1)
        return np.vectorize(self.preds_mapper.get)(outputs.astype(str))


[docs]    def predict_proba(self, X):
        """
        Make predictions for classification on a batch (valid)

        Parameters
        ----------
        X : a :tensor: `torch.Tensor` or matrix: `scipy.sparse.csr_matrix`
            Input data

        Returns
        -------
        res : np.ndarray

        """
        self.network.eval()

        if scipy.sparse.issparse(X):
            dataloader = DataLoader(
                SparsePredictDataset(X),
                batch_size=self.batch_size,
                shuffle=False,
            )
        else:
            dataloader = DataLoader(
                PredictDataset(X),
                batch_size=self.batch_size,
                shuffle=False,
            )

        results = []
        for batch_nb, data in enumerate(dataloader):
            data = data.to(self.device).float()

            output, M_loss = self.network(data)
            predictions = torch.nn.Softmax(dim=1)(output).cpu().detach().numpy()
            results.append(predictions)
        res = np.vstack(results)
        return res


[docs]class TabNetRegressor(TabModel):
    def __post_init__(self):
        super(TabNetRegressor, self).__post_init__()
        self._task = 'regression'
        self._default_loss = torch.nn.functional.mse_loss
        self._default_metric = 'mse'

[docs]    def prepare_target(self, y):
        return y


[docs]    def compute_loss(self, y_pred, y_true):
        return self.loss_fn(y_pred, y_true)


[docs]    def update_fit_params(
        self,
        X_train,
        y_train,
        eval_set,
        weights
    ):
        if len(y_train.shape) != 2:
            msg = "Targets should be 2D : (n_samples, n_regression) " + \
                  f"but y_train.shape={y_train.shape} given.\n" + \
                  "Use reshape(-1, 1) for single regression."
            raise ValueError(msg)
        self.output_dim = y_train.shape[1]
        self.preds_mapper = None

        self.updated_weights = weights
        filter_weights(self.updated_weights)


[docs]    def predict_func(self, outputs):
        return outputs


[docs]    def stack_batches(self, list_y_true, list_y_score):
        y_true = np.vstack(list_y_true)
        y_score = np.vstack(list_y_score)
        return y_true, y_score
```

---

© Copyright 2019, Dreamquark

Built with Sphinx using a
theme
provided by Read the Docs.
