# Supplementary material for: Development of a deep learning model for predicting recurrence of hepatocellular carcinoma after liver transplantation
Source: Front Med (Lausanne). 2024 Jun 11;11:1373005. doi: 10.3389/fmed.2024.1373005 (PMC11196752; doi:10.3389/fmed.2024.1373005)
Supplement: Supplementary file 1 [file Data_Sheet_1.ZIP › Raw data/source data and codes/codes/tabnet/docs/_modules/pytorch_tabnet/tab_network.html]

pytorch\_tabnet.tab\_network — pytorch\_tabnet documentation


pytorch\_tabnet

Contents:

- README
- TabNet : Attentive Interpretable Tabular Learning
- Installation
- What is new ?
- Contributing
- What problems does pytorch-tabnet handle?
- How to use it?
- Semi-supervised pre-training
- Data augmentation on the fly
- Easy saving and loading
- Useful links
- pytorch\_tabnet package

pytorch\_tabnet

- »
- Module code »
- pytorch\_tabnet.tab\_network

---

# Source code for pytorch\_tabnet.tab\_network

```
import torch
from torch.nn import Linear, BatchNorm1d, ReLU
import numpy as np
from pytorch_tabnet import sparsemax

[docs]def initialize_non_glu(module, input_dim, output_dim):
    gain_value = np.sqrt((input_dim + output_dim) / np.sqrt(4 * input_dim))
    torch.nn.init.xavier_normal_(module.weight, gain=gain_value)
    # torch.nn.init.zeros_(module.bias)
    return


[docs]def initialize_glu(module, input_dim, output_dim):
    gain_value = np.sqrt((input_dim + output_dim) / np.sqrt(input_dim))
    torch.nn.init.xavier_normal_(module.weight, gain=gain_value)
    # torch.nn.init.zeros_(module.bias)
    return


[docs]class GBN(torch.nn.Module):
    """
    Ghost Batch Normalization
    https://arxiv.org/abs/1705.08741
    """

    def __init__(self, input_dim, virtual_batch_size=128, momentum=0.01):
        super(GBN, self).__init__()

        self.input_dim = input_dim
        self.virtual_batch_size = virtual_batch_size
        self.bn = BatchNorm1d(self.input_dim, momentum=momentum)

[docs]    def forward(self, x):
        chunks = x.chunk(int(np.ceil(x.shape[0] / self.virtual_batch_size)), 0)
        res = [self.bn(x_) for x_ in chunks]

        return torch.cat(res, dim=0)


[docs]class TabNetEncoder(torch.nn.Module):
    def __init__(
        self,
        input_dim,
        output_dim,
        n_d=8,
        n_a=8,
        n_steps=3,
        gamma=1.3,
        n_independent=2,
        n_shared=2,
        epsilon=1e-15,
        virtual_batch_size=128,
        momentum=0.02,
        mask_type="sparsemax",
        group_attention_matrix=None,
    ):
        """
        Defines main part of the TabNet network without the embedding layers.

        Parameters
        ----------
        input_dim : int
            Number of features
        output_dim : int or list of int for multi task classification
            Dimension of network output
            examples : one for regression, 2 for binary classification etc...
        n_d : int
            Dimension of the prediction  layer (usually between 4 and 64)
        n_a : int
            Dimension of the attention  layer (usually between 4 and 64)
        n_steps : int
            Number of successive steps in the network (usually between 3 and 10)
        gamma : float
            Float above 1, scaling factor for attention updates (usually between 1.0 to 2.0)
        n_independent : int
            Number of independent GLU layer in each GLU block (default 2)
        n_shared : int
            Number of independent GLU layer in each GLU block (default 2)
        epsilon : float
            Avoid log(0), this should be kept very low
        virtual_batch_size : int
            Batch size for Ghost Batch Normalization
        momentum : float
            Float value between 0 and 1 which will be used for momentum in all batch norm
        mask_type : str
            Either "sparsemax" or "entmax" : this is the masking function to use
        group_attention_matrix : torch matrix
            Matrix of size (n_groups, input_dim), m_ij = importance within group i of feature j
        """
        super(TabNetEncoder, self).__init__()
        self.input_dim = input_dim
        self.output_dim = output_dim
        self.is_multi_task = isinstance(output_dim, list)
        self.n_d = n_d
        self.n_a = n_a
        self.n_steps = n_steps
        self.gamma = gamma
        self.epsilon = epsilon
        self.n_independent = n_independent
        self.n_shared = n_shared
        self.virtual_batch_size = virtual_batch_size
        self.mask_type = mask_type
        self.initial_bn = BatchNorm1d(self.input_dim, momentum=0.01)
        self.group_attention_matrix = group_attention_matrix

        if self.group_attention_matrix is None:
            # no groups
            self.group_attention_matrix = torch.eye(self.input_dim)
            self.attention_dim = self.input_dim
        else:
            self.attention_dim = self.group_attention_matrix.shape[0]

        if self.n_shared > 0:
            shared_feat_transform = torch.nn.ModuleList()
            for i in range(self.n_shared):
                if i == 0:
                    shared_feat_transform.append(
                        Linear(self.input_dim, 2 * (n_d + n_a), bias=False)
                    )
                else:
                    shared_feat_transform.append(
                        Linear(n_d + n_a, 2 * (n_d + n_a), bias=False)
                    )

        else:
            shared_feat_transform = None

        self.initial_splitter = FeatTransformer(
            self.input_dim,
            n_d + n_a,
            shared_feat_transform,
            n_glu_independent=self.n_independent,
            virtual_batch_size=self.virtual_batch_size,
            momentum=momentum,
        )

        self.feat_transformers = torch.nn.ModuleList()
        self.att_transformers = torch.nn.ModuleList()

        for step in range(n_steps):
            transformer = FeatTransformer(
                self.input_dim,
                n_d + n_a,
                shared_feat_transform,
                n_glu_independent=self.n_independent,
                virtual_batch_size=self.virtual_batch_size,
                momentum=momentum,
            )
            attention = AttentiveTransformer(
                n_a,
                self.attention_dim,
                group_matrix=group_attention_matrix,
                virtual_batch_size=self.virtual_batch_size,
                momentum=momentum,
                mask_type=self.mask_type,
            )
            self.feat_transformers.append(transformer)
            self.att_transformers.append(attention)

[docs]    def forward(self, x, prior=None):
        x = self.initial_bn(x)

        bs = x.shape[0]  # batch size
        if prior is None:
            prior = torch.ones((bs, self.attention_dim)).to(x.device)

        M_loss = 0
        att = self.initial_splitter(x)[:, self.n_d :]
        steps_output = []
        for step in range(self.n_steps):
            M = self.att_transformers[step](prior, att)
            M_loss += torch.mean(
                torch.sum(torch.mul(M, torch.log(M + self.epsilon)), dim=1)
            )
            # update prior
            prior = torch.mul(self.gamma - M, prior)
            # output
            M_feature_level = torch.matmul(M, self.group_attention_matrix)
            masked_x = torch.mul(M_feature_level, x)
            out = self.feat_transformers[step](masked_x)
            d = ReLU()(out[:, : self.n_d])
            steps_output.append(d)
            # update attention
            att = out[:, self.n_d :]

        M_loss /= self.n_steps
        return steps_output, M_loss


[docs]    def forward_masks(self, x):
        x = self.initial_bn(x)
        bs = x.shape[0]  # batch size
        prior = torch.ones((bs, self.attention_dim)).to(x.device)
        M_explain = torch.zeros(x.shape).to(x.device)
        att = self.initial_splitter(x)[:, self.n_d :]
        masks = {}

        for step in range(self.n_steps):
            M = self.att_transformers[step](prior, att)
            M_feature_level = torch.matmul(M, self.group_attention_matrix)
            masks[step] = M_feature_level
            # update prior
            prior = torch.mul(self.gamma - M, prior)
            # output
            masked_x = torch.mul(M_feature_level, x)
            out = self.feat_transformers[step](masked_x)
            d = ReLU()(out[:, : self.n_d])
            # explain
            step_importance = torch.sum(d, dim=1)
            M_explain += torch.mul(M_feature_level, step_importance.unsqueeze(dim=1))
            # update attention
            att = out[:, self.n_d :]

        return M_explain, masks


[docs]class TabNetDecoder(torch.nn.Module):
    def __init__(
        self,
        input_dim,
        n_d=8,
        n_steps=3,
        n_independent=1,
        n_shared=1,
        virtual_batch_size=128,
        momentum=0.02,
    ):
        """
        Defines main part of the TabNet network without the embedding layers.

        Parameters
        ----------
        input_dim : int
            Number of features
        output_dim : int or list of int for multi task classification
            Dimension of network output
            examples : one for regression, 2 for binary classification etc...
        n_d : int
            Dimension of the prediction  layer (usually between 4 and 64)
        n_steps : int
            Number of successive steps in the network (usually between 3 and 10)
        gamma : float
            Float above 1, scaling factor for attention updates (usually between 1.0 to 2.0)
        n_independent : int
            Number of independent GLU layer in each GLU block (default 1)
        n_shared : int
            Number of independent GLU layer in each GLU block (default 1)
        virtual_batch_size : int
            Batch size for Ghost Batch Normalization
        momentum : float
            Float value between 0 and 1 which will be used for momentum in all batch norm
        """
        super(TabNetDecoder, self).__init__()
        self.input_dim = input_dim
        self.n_d = n_d
        self.n_steps = n_steps
        self.n_independent = n_independent
        self.n_shared = n_shared
        self.virtual_batch_size = virtual_batch_size

        self.feat_transformers = torch.nn.ModuleList()

        if self.n_shared > 0:
            shared_feat_transform = torch.nn.ModuleList()
            for i in range(self.n_shared):
                shared_feat_transform.append(Linear(n_d, 2 * n_d, bias=False))
        else:
            shared_feat_transform = None

        for step in range(n_steps):
            transformer = FeatTransformer(
                n_d,
                n_d,
                shared_feat_transform,
                n_glu_independent=self.n_independent,
                virtual_batch_size=self.virtual_batch_size,
                momentum=momentum,
            )
            self.feat_transformers.append(transformer)

        self.reconstruction_layer = Linear(n_d, self.input_dim, bias=False)
        initialize_non_glu(self.reconstruction_layer, n_d, self.input_dim)

[docs]    def forward(self, steps_output):
        res = 0
        for step_nb, step_output in enumerate(steps_output):
            x = self.feat_transformers[step_nb](step_output)
            res = torch.add(res, x)
        res = self.reconstruction_layer(res)
        return res


[docs]class TabNetPretraining(torch.nn.Module):
    def __init__(
        self,
        input_dim,
        pretraining_ratio=0.2,
        n_d=8,
        n_a=8,
        n_steps=3,
        gamma=1.3,
        cat_idxs=[],
        cat_dims=[],
        cat_emb_dim=1,
        n_independent=2,
        n_shared=2,
        epsilon=1e-15,
        virtual_batch_size=128,
        momentum=0.02,
        mask_type="sparsemax",
        n_shared_decoder=1,
        n_indep_decoder=1,
        group_attention_matrix=None,
    ):
        super(TabNetPretraining, self).__init__()

        self.cat_idxs = cat_idxs or []
        self.cat_dims = cat_dims or []
        self.cat_emb_dim = cat_emb_dim

        self.input_dim = input_dim
        self.n_d = n_d
        self.n_a = n_a
        self.n_steps = n_steps
        self.gamma = gamma
        self.epsilon = epsilon
        self.n_independent = n_independent
        self.n_shared = n_shared
        self.mask_type = mask_type
        self.pretraining_ratio = pretraining_ratio
        self.n_shared_decoder = n_shared_decoder
        self.n_indep_decoder = n_indep_decoder

        if self.n_steps <= 0:
            raise ValueError("n_steps should be a positive integer.")
        if self.n_independent == 0 and self.n_shared == 0:
            raise ValueError("n_shared and n_independent can't be both zero.")

        self.virtual_batch_size = virtual_batch_size
        self.embedder = EmbeddingGenerator(input_dim,
                                           cat_dims,
                                           cat_idxs,
                                           cat_emb_dim,
                                           group_attention_matrix)
        self.post_embed_dim = self.embedder.post_embed_dim

        self.masker = RandomObfuscator(self.pretraining_ratio,
                                       group_matrix=self.embedder.embedding_group_matrix)
        self.encoder = TabNetEncoder(
            input_dim=self.post_embed_dim,
            output_dim=self.post_embed_dim,
            n_d=n_d,
            n_a=n_a,
            n_steps=n_steps,
            gamma=gamma,
            n_independent=n_independent,
            n_shared=n_shared,
            epsilon=epsilon,
            virtual_batch_size=virtual_batch_size,
            momentum=momentum,
            mask_type=mask_type,
            group_attention_matrix=self.embedder.embedding_group_matrix,
        )
        self.decoder = TabNetDecoder(
            self.post_embed_dim,
            n_d=n_d,
            n_steps=n_steps,
            n_independent=self.n_indep_decoder,
            n_shared=self.n_shared_decoder,
            virtual_batch_size=virtual_batch_size,
            momentum=momentum,
        )

[docs]    def forward(self, x):
        """
        Returns: res, embedded_x, obf_vars
            res : output of reconstruction
            embedded_x : embedded input
            obf_vars : which variable where obfuscated
        """
        embedded_x = self.embedder(x)
        if self.training:
            masked_x, obfuscated_groups, obfuscated_vars = self.masker(embedded_x)
            # set prior of encoder with obfuscated groups
            prior = 1 - obfuscated_groups
            steps_out, _ = self.encoder(masked_x, prior=prior)
            res = self.decoder(steps_out)
            return res, embedded_x, obfuscated_vars
        else:
            steps_out, _ = self.encoder(embedded_x)
            res = self.decoder(steps_out)
            return res, embedded_x, torch.ones(embedded_x.shape).to(x.device)


[docs]    def forward_masks(self, x):
        embedded_x = self.embedder(x)
        return self.encoder.forward_masks(embedded_x)


[docs]class TabNetNoEmbeddings(torch.nn.Module):
    def __init__(
        self,
        input_dim,
        output_dim,
        n_d=8,
        n_a=8,
        n_steps=3,
        gamma=1.3,
        n_independent=2,
        n_shared=2,
        epsilon=1e-15,
        virtual_batch_size=128,
        momentum=0.02,
        mask_type="sparsemax",
        group_attention_matrix=None,
    ):
        """
        Defines main part of the TabNet network without the embedding layers.

        Parameters
        ----------
        input_dim : int
            Number of features
        output_dim : int or list of int for multi task classification
            Dimension of network output
            examples : one for regression, 2 for binary classification etc...
        n_d : int
            Dimension of the prediction  layer (usually between 4 and 64)
        n_a : int
            Dimension of the attention  layer (usually between 4 and 64)
        n_steps : int
            Number of successive steps in the network (usually between 3 and 10)
        gamma : float
            Float above 1, scaling factor for attention updates (usually between 1.0 to 2.0)
        n_independent : int
            Number of independent GLU layer in each GLU block (default 2)
        n_shared : int
            Number of independent GLU layer in each GLU block (default 2)
        epsilon : float
            Avoid log(0), this should be kept very low
        virtual_batch_size : int
            Batch size for Ghost Batch Normalization
        momentum : float
            Float value between 0 and 1 which will be used for momentum in all batch norm
        mask_type : str
            Either "sparsemax" or "entmax" : this is the masking function to use
        group_attention_matrix : torch matrix
            Matrix of size (n_groups, input_dim), m_ij = importance within group i of feature j
        """
        super(TabNetNoEmbeddings, self).__init__()
        self.input_dim = input_dim
        self.output_dim = output_dim
        self.is_multi_task = isinstance(output_dim, list)
        self.n_d = n_d
        self.n_a = n_a
        self.n_steps = n_steps
        self.gamma = gamma
        self.epsilon = epsilon
        self.n_independent = n_independent
        self.n_shared = n_shared
        self.virtual_batch_size = virtual_batch_size
        self.mask_type = mask_type
        self.initial_bn = BatchNorm1d(self.input_dim, momentum=0.01)

        self.encoder = TabNetEncoder(
            input_dim=input_dim,
            output_dim=output_dim,
            n_d=n_d,
            n_a=n_a,
            n_steps=n_steps,
            gamma=gamma,
            n_independent=n_independent,
            n_shared=n_shared,
            epsilon=epsilon,
            virtual_batch_size=virtual_batch_size,
            momentum=momentum,
            mask_type=mask_type,
            group_attention_matrix=group_attention_matrix
        )

        if self.is_multi_task:
            self.multi_task_mappings = torch.nn.ModuleList()
            for task_dim in output_dim:
                task_mapping = Linear(n_d, task_dim, bias=False)
                initialize_non_glu(task_mapping, n_d, task_dim)
                self.multi_task_mappings.append(task_mapping)
        else:
            self.final_mapping = Linear(n_d, output_dim, bias=False)
            initialize_non_glu(self.final_mapping, n_d, output_dim)

[docs]    def forward(self, x):
        res = 0
        steps_output, M_loss = self.encoder(x)
        res = torch.sum(torch.stack(steps_output, dim=0), dim=0)

        if self.is_multi_task:
            # Result will be in list format
            out = []
            for task_mapping in self.multi_task_mappings:
                out.append(task_mapping(res))
        else:
            out = self.final_mapping(res)
        return out, M_loss


[docs]    def forward_masks(self, x):
        return self.encoder.forward_masks(x)


[docs]class TabNet(torch.nn.Module):
    def __init__(
        self,
        input_dim,
        output_dim,
        n_d=8,
        n_a=8,
        n_steps=3,
        gamma=1.3,
        cat_idxs=[],
        cat_dims=[],
        cat_emb_dim=1,
        n_independent=2,
        n_shared=2,
        epsilon=1e-15,
        virtual_batch_size=128,
        momentum=0.02,
        mask_type="sparsemax",
        group_attention_matrix=[],
    ):
        """
        Defines TabNet network

        Parameters
        ----------
        input_dim : int
            Initial number of features
        output_dim : int
            Dimension of network output
            examples : one for regression, 2 for binary classification etc...
        n_d : int
            Dimension of the prediction  layer (usually between 4 and 64)
        n_a : int
            Dimension of the attention  layer (usually between 4 and 64)
        n_steps : int
            Number of successive steps in the network (usually between 3 and 10)
        gamma : float
            Float above 1, scaling factor for attention updates (usually between 1.0 to 2.0)
        cat_idxs : list of int
            Index of each categorical column in the dataset
        cat_dims : list of int
            Number of categories in each categorical column
        cat_emb_dim : int or list of int
            Size of the embedding of categorical features
            if int, all categorical features will have same embedding size
            if list of int, every corresponding feature will have specific size
        n_independent : int
            Number of independent GLU layer in each GLU block (default 2)
        n_shared : int
            Number of independent GLU layer in each GLU block (default 2)
        epsilon : float
            Avoid log(0), this should be kept very low
        virtual_batch_size : int
            Batch size for Ghost Batch Normalization
        momentum : float
            Float value between 0 and 1 which will be used for momentum in all batch norm
        mask_type : str
            Either "sparsemax" or "entmax" : this is the masking function to use
        group_attention_matrix : torch matrix
            Matrix of size (n_groups, input_dim), m_ij = importance within group i of feature j
        """
        super(TabNet, self).__init__()
        self.cat_idxs = cat_idxs or []
        self.cat_dims = cat_dims or []
        self.cat_emb_dim = cat_emb_dim

        self.input_dim = input_dim
        self.output_dim = output_dim
        self.n_d = n_d
        self.n_a = n_a
        self.n_steps = n_steps
        self.gamma = gamma
        self.epsilon = epsilon
        self.n_independent = n_independent
        self.n_shared = n_shared
        self.mask_type = mask_type

        if self.n_steps <= 0:
            raise ValueError("n_steps should be a positive integer.")
        if self.n_independent == 0 and self.n_shared == 0:
            raise ValueError("n_shared and n_independent can't be both zero.")

        self.virtual_batch_size = virtual_batch_size
        self.embedder = EmbeddingGenerator(input_dim,
                                           cat_dims,
                                           cat_idxs,
                                           cat_emb_dim,
                                           group_attention_matrix)
        self.post_embed_dim = self.embedder.post_embed_dim

        self.tabnet = TabNetNoEmbeddings(
            self.post_embed_dim,
            output_dim,
            n_d,
            n_a,
            n_steps,
            gamma,
            n_independent,
            n_shared,
            epsilon,
            virtual_batch_size,
            momentum,
            mask_type,
            self.embedder.embedding_group_matrix
        )

[docs]    def forward(self, x):
        x = self.embedder(x)
        return self.tabnet(x)


[docs]    def forward_masks(self, x):
        x = self.embedder(x)
        return self.tabnet.forward_masks(x)


[docs]class AttentiveTransformer(torch.nn.Module):
    def __init__(
        self,
        input_dim,
        group_dim,
        group_matrix,
        virtual_batch_size=128,
        momentum=0.02,
        mask_type="sparsemax",
    ):
        """
        Initialize an attention transformer.

        Parameters
        ----------
        input_dim : int
            Input size
        group_dim : int
            Number of groups for features
        virtual_batch_size : int
            Batch size for Ghost Batch Normalization
        momentum : float
            Float value between 0 and 1 which will be used for momentum in batch norm
        mask_type : str
            Either "sparsemax" or "entmax" : this is the masking function to use
        """
        super(AttentiveTransformer, self).__init__()
        self.fc = Linear(input_dim, group_dim, bias=False)
        initialize_non_glu(self.fc, input_dim, group_dim)
        self.bn = GBN(
            group_dim, virtual_batch_size=virtual_batch_size, momentum=momentum
        )

        if mask_type == "sparsemax":
            # Sparsemax
            self.selector = sparsemax.Sparsemax(dim=-1)
        elif mask_type == "entmax":
            # Entmax
            self.selector = sparsemax.Entmax15(dim=-1)
        else:
            raise NotImplementedError(
                "Please choose either sparsemax" + "or entmax as masktype"
            )

[docs]    def forward(self, priors, processed_feat):
        x = self.fc(processed_feat)
        x = self.bn(x)
        x = torch.mul(x, priors)
        x = self.selector(x)
        return x


[docs]class FeatTransformer(torch.nn.Module):
    def __init__(
        self,
        input_dim,
        output_dim,
        shared_layers,
        n_glu_independent,
        virtual_batch_size=128,
        momentum=0.02,
    ):
        super(FeatTransformer, self).__init__()
        """
        Initialize a feature transformer.

        Parameters
        ----------
        input_dim : int
            Input size
        output_dim : int
            Output_size
        shared_layers : torch.nn.ModuleList
            The shared block that should be common to every step
        n_glu_independent : int
            Number of independent GLU layers
        virtual_batch_size : int
            Batch size for Ghost Batch Normalization within GLU block(s)
        momentum : float
            Float value between 0 and 1 which will be used for momentum in batch norm
        """

        params = {
            "n_glu": n_glu_independent,
            "virtual_batch_size": virtual_batch_size,
            "momentum": momentum,
        }

        if shared_layers is None:
            # no shared layers
            self.shared = torch.nn.Identity()
            is_first = True
        else:
            self.shared = GLU_Block(
                input_dim,
                output_dim,
                first=True,
                shared_layers=shared_layers,
                n_glu=len(shared_layers),
                virtual_batch_size=virtual_batch_size,
                momentum=momentum,
            )
            is_first = False

        if n_glu_independent == 0:
            # no independent layers
            self.specifics = torch.nn.Identity()
        else:
            spec_input_dim = input_dim if is_first else output_dim
            self.specifics = GLU_Block(
                spec_input_dim, output_dim, first=is_first, **params
            )

[docs]    def forward(self, x):
        x = self.shared(x)
        x = self.specifics(x)
        return x


[docs]class GLU_Block(torch.nn.Module):
    """
    Independent GLU block, specific to each step
    """

    def __init__(
        self,
        input_dim,
        output_dim,
        n_glu=2,
        first=False,
        shared_layers=None,
        virtual_batch_size=128,
        momentum=0.02,
    ):
        super(GLU_Block, self).__init__()
        self.first = first
        self.shared_layers = shared_layers
        self.n_glu = n_glu
        self.glu_layers = torch.nn.ModuleList()

        params = {"virtual_batch_size": virtual_batch_size, "momentum": momentum}

        fc = shared_layers[0] if shared_layers else None
        self.glu_layers.append(GLU_Layer(input_dim, output_dim, fc=fc, **params))
        for glu_id in range(1, self.n_glu):
            fc = shared_layers[glu_id] if shared_layers else None
            self.glu_layers.append(GLU_Layer(output_dim, output_dim, fc=fc, **params))

[docs]    def forward(self, x):
        scale = torch.sqrt(torch.FloatTensor([0.5]).to(x.device))
        if self.first:  # the first layer of the block has no scale multiplication
            x = self.glu_layers[0](x)
            layers_left = range(1, self.n_glu)
        else:
            layers_left = range(self.n_glu)

        for glu_id in layers_left:
            x = torch.add(x, self.glu_layers[glu_id](x))
            x = x * scale
        return x


[docs]class GLU_Layer(torch.nn.Module):
    def __init__(
        self, input_dim, output_dim, fc=None, virtual_batch_size=128, momentum=0.02
    ):
        super(GLU_Layer, self).__init__()

        self.output_dim = output_dim
        if fc:
            self.fc = fc
        else:
            self.fc = Linear(input_dim, 2 * output_dim, bias=False)
        initialize_glu(self.fc, input_dim, 2 * output_dim)

        self.bn = GBN(
            2 * output_dim, virtual_batch_size=virtual_batch_size, momentum=momentum
        )

[docs]    def forward(self, x):
        x = self.fc(x)
        x = self.bn(x)
        out = torch.mul(x[:, : self.output_dim], torch.sigmoid(x[:, self.output_dim :]))
        return out


[docs]class EmbeddingGenerator(torch.nn.Module):
    """
    Classical embeddings generator
    """

    def __init__(self, input_dim, cat_dims, cat_idxs, cat_emb_dims, group_matrix):
        """This is an embedding module for an entire set of features

        Parameters
        ----------
        input_dim : int
            Number of features coming as input (number of columns)
        cat_dims : list of int
            Number of modalities for each categorial features
            If the list is empty, no embeddings will be done
        cat_idxs : list of int
            Positional index for each categorical features in inputs
        cat_emb_dim : list of int
            Embedding dimension for each categorical features
            If int, the same embedding dimension will be used for all categorical features
        group_matrix : torch matrix
            Original group matrix before embeddings
        """
        super(EmbeddingGenerator, self).__init__()

        if cat_dims == [] and cat_idxs == []:
            self.skip_embedding = True
            self.post_embed_dim = input_dim
            self.embedding_group_matrix = group_matrix.to(group_matrix.device)
            return
        else:
            self.skip_embedding = False

        self.post_embed_dim = int(input_dim + np.sum(cat_emb_dims) - len(cat_emb_dims))

        self.embeddings = torch.nn.ModuleList()

        for cat_dim, emb_dim in zip(cat_dims, cat_emb_dims):
            self.embeddings.append(torch.nn.Embedding(cat_dim, emb_dim))

        # record continuous indices
        self.continuous_idx = torch.ones(input_dim, dtype=torch.bool)
        self.continuous_idx[cat_idxs] = 0

        # update group matrix
        n_groups = group_matrix.shape[0]
        self.embedding_group_matrix = torch.empty((n_groups, self.post_embed_dim),
                                                  device=group_matrix.device)
        for group_idx in range(n_groups):
            post_emb_idx = 0
            cat_feat_counter = 0
            for init_feat_idx in range(input_dim):
                if self.continuous_idx[init_feat_idx] == 1:
                    # this means that no embedding is applied to this column
                    self.embedding_group_matrix[group_idx, post_emb_idx] = group_matrix[group_idx, init_feat_idx]  # noqa
                    post_emb_idx += 1
                else:
                    # this is a categorical feature which creates multiple embeddings
                    n_embeddings = cat_emb_dims[cat_feat_counter]
                    self.embedding_group_matrix[group_idx, post_emb_idx:post_emb_idx+n_embeddings] = group_matrix[group_idx, init_feat_idx] / n_embeddings  # noqa
                    post_emb_idx += n_embeddings
                    cat_feat_counter += 1

[docs]    def forward(self, x):
        """
        Apply embeddings to inputs
        Inputs should be (batch_size, input_dim)
        Outputs will be of size (batch_size, self.post_embed_dim)
        """
        if self.skip_embedding:
            # no embeddings required
            return x

        cols = []
        cat_feat_counter = 0
        for feat_init_idx, is_continuous in enumerate(self.continuous_idx):
            # Enumerate through continuous idx boolean mask to apply embeddings
            if is_continuous:
                cols.append(x[:, feat_init_idx].float().view(-1, 1))
            else:
                cols.append(
                    self.embeddings[cat_feat_counter](x[:, feat_init_idx].long())
                )
                cat_feat_counter += 1
        # concat
        post_embeddings = torch.cat(cols, dim=1)
        return post_embeddings


[docs]class RandomObfuscator(torch.nn.Module):
    """
    Create and applies obfuscation masks.
    The obfuscation is done at group level to match attention.
    """

    def __init__(self, pretraining_ratio, group_matrix):
        """
        This create random obfuscation for self suppervised pretraining
        Parameters
        ----------
        pretraining_ratio : float
            Ratio of feature to randomly discard for reconstruction

        """
        super(RandomObfuscator, self).__init__()
        self.pretraining_ratio = pretraining_ratio
        # group matrix is set to boolean here to pass all posssible information
        self.group_matrix = (group_matrix > 0) + 0.
        self.num_groups = group_matrix.shape[0]

[docs]    def forward(self, x):
        """
        Generate random obfuscation mask.

        Returns
        -------
        masked input and obfuscated variables.
        """
        bs = x.shape[0]

        obfuscated_groups = torch.bernoulli(
            self.pretraining_ratio * torch.ones((bs, self.num_groups), device=x.device)
        )
        obfuscated_vars = torch.matmul(obfuscated_groups, self.group_matrix)
        masked_input = torch.mul(1 - obfuscated_vars, x)
        return masked_input, obfuscated_groups, obfuscated_vars
```

---

© Copyright 2019, Dreamquark

Built with Sphinx using a
theme
provided by Read the Docs.
