# Supplementary material for: Development of a deep learning model for predicting recurrence of hepatocellular carcinoma after liver transplantation
Source: Front Med (Lausanne). 2024 Jun 11;11:1373005. doi: 10.3389/fmed.2024.1373005 (PMC11196752; doi:10.3389/fmed.2024.1373005)
Supplement: Supplementary file 1 [file Data_Sheet_1.ZIP › Raw data/source data and codes/codes/tabnet/docs/_modules/pytorch_tabnet/utils.html]

pytorch\_tabnet.utils — pytorch\_tabnet documentation


pytorch\_tabnet

Contents:

- README
- TabNet : Attentive Interpretable Tabular Learning
- Installation
- What is new ?
- Contributing
- What problems does pytorch-tabnet handle?
- How to use it?
- Semi-supervised pre-training
- Data augmentation on the fly
- Easy saving and loading
- Useful links
- pytorch\_tabnet package

pytorch\_tabnet

- »
- Module code »
- pytorch\_tabnet.utils

---

# Source code for pytorch\_tabnet.utils

```
from torch.utils.data import Dataset
from torch.utils.data import DataLoader, WeightedRandomSampler
import torch
import numpy as np
import scipy
import json
from sklearn.utils import check_array
import pandas as pd
import warnings

[docs]class TorchDataset(Dataset):
    """
    Format for numpy array

    Parameters
    ----------
    X : 2D array
        The input matrix
    y : 2D array
        The one-hot encoded target
    """

    def __init__(self, x, y):
        self.x = x
        self.y = y

    def __len__(self):
        return len(self.x)

    def __getitem__(self, index):
        x, y = self.x[index], self.y[index]
        return x, y


[docs]class SparseTorchDataset(Dataset):
    """
    Format for csr_matrix

    Parameters
    ----------
    X : CSR matrix
        The input matrix
    y : 2D array
        The one-hot encoded target
    """

    def __init__(self, x, y):
        self.x = x
        self.y = y

    def __len__(self):
        return self.x.shape[0]

    def __getitem__(self, index):
        x = torch.from_numpy(self.x[index].toarray()[0]).float()
        y = self.y[index]
        return x, y


[docs]class PredictDataset(Dataset):
    """
    Format for numpy array

    Parameters
    ----------
    X : 2D array
        The input matrix
    """

    def __init__(self, x):
        self.x = x

    def __len__(self):
        return len(self.x)

    def __getitem__(self, index):
        x = self.x[index]
        return x


[docs]class SparsePredictDataset(Dataset):
    """
    Format for csr_matrix

    Parameters
    ----------
    X : CSR matrix
        The input matrix
    """

    def __init__(self, x):
        self.x = x

    def __len__(self):
        return self.x.shape[0]

    def __getitem__(self, index):
        x = torch.from_numpy(self.x[index].toarray()[0]).float()
        return x


[docs]def create_sampler(weights, y_train):
    """
    This creates a sampler from the given weights

    Parameters
    ----------
    weights : either 0, 1, dict or iterable
        if 0 (default) : no weights will be applied
        if 1 : classification only, will balanced class with inverse frequency
        if dict : keys are corresponding class values are sample weights
        if iterable : list or np array must be of length equal to nb elements
                      in the training set
    y_train : np.array
        Training targets
    """
    if isinstance(weights, int):
        if weights == 0:
            need_shuffle = True
            sampler = None
        elif weights == 1:
            need_shuffle = False
            class_sample_count = np.array(
                [len(np.where(y_train == t)[0]) for t in np.unique(y_train)]
            )

            weights = 1.0 / class_sample_count

            samples_weight = np.array([weights[t] for t in y_train])

            samples_weight = torch.from_numpy(samples_weight)
            samples_weight = samples_weight.double()
            sampler = WeightedRandomSampler(samples_weight, len(samples_weight))
        else:
            raise ValueError("Weights should be either 0, 1, dictionnary or list.")
    elif isinstance(weights, dict):
        # custom weights per class
        need_shuffle = False
        samples_weight = np.array([weights[t] for t in y_train])
        sampler = WeightedRandomSampler(samples_weight, len(samples_weight))
    else:
        # custom weights
        if len(weights) != len(y_train):
            raise ValueError("Custom weights should match number of train samples.")
        need_shuffle = False
        samples_weight = np.array(weights)
        sampler = WeightedRandomSampler(samples_weight, len(samples_weight))
    return need_shuffle, sampler


[docs]def create_dataloaders(
    X_train, y_train, eval_set, weights, batch_size, num_workers, drop_last, pin_memory
):
    """
    Create dataloaders with or without subsampling depending on weights and balanced.

    Parameters
    ----------
    X_train : np.ndarray
        Training data
    y_train : np.array
        Mapped Training targets
    eval_set : list of tuple
        List of eval tuple set (X, y)
    weights : either 0, 1, dict or iterable
        if 0 (default) : no weights will be applied
        if 1 : classification only, will balanced class with inverse frequency
        if dict : keys are corresponding class values are sample weights
        if iterable : list or np array must be of length equal to nb elements
                      in the training set
    batch_size : int
        how many samples per batch to load
    num_workers : int
        how many subprocesses to use for data loading. 0 means that the data
        will be loaded in the main process
    drop_last : bool
        set to True to drop the last incomplete batch, if the dataset size is not
        divisible by the batch size. If False and the size of dataset is not
        divisible by the batch size, then the last batch will be smaller
    pin_memory : bool
        Whether to pin GPU memory during training

    Returns
    -------
    train_dataloader, valid_dataloader : torch.DataLoader, torch.DataLoader
        Training and validation dataloaders
    """
    need_shuffle, sampler = create_sampler(weights, y_train)

    if scipy.sparse.issparse(X_train):
        train_dataloader = DataLoader(
            SparseTorchDataset(X_train.astype(np.float32), y_train),
            batch_size=batch_size,
            sampler=sampler,
            shuffle=need_shuffle,
            num_workers=num_workers,
            drop_last=drop_last,
            pin_memory=pin_memory,
        )
    else:
        train_dataloader = DataLoader(
            TorchDataset(X_train.astype(np.float32), y_train),
            batch_size=batch_size,
            sampler=sampler,
            shuffle=need_shuffle,
            num_workers=num_workers,
            drop_last=drop_last,
            pin_memory=pin_memory,
        )

    valid_dataloaders = []
    for X, y in eval_set:
        if scipy.sparse.issparse(X):
            valid_dataloaders.append(
                DataLoader(
                    SparseTorchDataset(X.astype(np.float32), y),
                    batch_size=batch_size,
                    shuffle=False,
                    num_workers=num_workers,
                    pin_memory=pin_memory,
                )
            )
        else:
            valid_dataloaders.append(
                DataLoader(
                    TorchDataset(X.astype(np.float32), y),
                    batch_size=batch_size,
                    shuffle=False,
                    num_workers=num_workers,
                    pin_memory=pin_memory,
                )
            )

    return train_dataloader, valid_dataloaders


[docs]def create_explain_matrix(input_dim, cat_emb_dim, cat_idxs, post_embed_dim):
    """
    This is a computational trick.
    In order to rapidly sum importances from same embeddings
    to the initial index.

    Parameters
    ----------
    input_dim : int
        Initial input dim
    cat_emb_dim : int or list of int
        if int : size of embedding for all categorical feature
        if list of int : size of embedding for each categorical feature
    cat_idxs : list of int
        Initial position of categorical features
    post_embed_dim : int
        Post embedding inputs dimension

    Returns
    -------
    reducing_matrix : np.array
        Matrix of dim (post_embed_dim, input_dim)  to performe reduce
    """

    if isinstance(cat_emb_dim, int):
        all_emb_impact = [cat_emb_dim - 1] * len(cat_idxs)
    else:
        all_emb_impact = [emb_dim - 1 for emb_dim in cat_emb_dim]

    acc_emb = 0
    nb_emb = 0
    indices_trick = []
    for i in range(input_dim):
        if i not in cat_idxs:
            indices_trick.append([i + acc_emb])
        else:
            indices_trick.append(
                range(i + acc_emb, i + acc_emb + all_emb_impact[nb_emb] + 1)
            )
            acc_emb += all_emb_impact[nb_emb]
            nb_emb += 1

    reducing_matrix = np.zeros((post_embed_dim, input_dim))
    for i, cols in enumerate(indices_trick):
        reducing_matrix[cols, i] = 1

    return scipy.sparse.csc_matrix(reducing_matrix)


[docs]def create_group_matrix(list_groups, input_dim):
    """
    Create the group matrix corresponding to the given list_groups

    Parameters
    ----------
    - list_groups : list of list of int
        Each element is a list representing features in the same group.
        One feature should appear in maximum one group.
        Feature that don't get assigned a group will be in their own group of one feature.
    - input_dim : number of feature in the initial dataset

    Returns
    -------
    - group_matrix : torch matrix
        A matrix of size (n_groups, input_dim)
        where m_ij represents the importance of feature j in group i
        The rows must some to 1 as each group is equally important a priori.

    """
    check_list_groups(list_groups, input_dim)

    if len(list_groups) == 0:
        group_matrix = torch.eye(input_dim)
        return group_matrix
    else:
        n_groups = input_dim - int(np.sum([len(gp) - 1 for gp in list_groups]))
        group_matrix = torch.zeros((n_groups, input_dim))

        remaining_features = [feat_idx for feat_idx in range(input_dim)]

        current_group_idx = 0
        for group in list_groups:
            group_size = len(group)
            for elem_idx in group:
                # add importrance of element in group matrix and corresponding group
                group_matrix[current_group_idx, elem_idx] = 1 / group_size
                # remove features from list of features
                remaining_features.remove(elem_idx)
            # move to next group
            current_group_idx += 1
        # features not mentionned in list_groups get assigned their own group of singleton
        for remaining_feat_idx in remaining_features:
            group_matrix[current_group_idx, remaining_feat_idx] = 1
            current_group_idx += 1
        return group_matrix


[docs]def check_list_groups(list_groups, input_dim):
    """
    Check that list groups:
        - is a list of list
        - does not contain twice the same feature in different groups
        - does not contain unknown features (>= input_dim)
        - does not contain empty groups
    Parameters
    ----------
    - list_groups : list of list of int
        Each element is a list representing features in the same group.
        One feature should appear in maximum one group.
        Feature that don't get assign a group will be in their own group of one feature.
    - input_dim : number of feature in the initial dataset
    """
    assert isinstance(list_groups, list), "list_groups must be a list of list."

    if len(list_groups) == 0:
        return
    else:
        for group_pos, group in enumerate(list_groups):
            msg = f"Groups must be given as a list of list, but found {group} in position {group_pos}."  # noqa
            assert isinstance(group, list), msg
            assert len(group) > 0, "Empty groups are forbidding please remove empty groups []"

    n_elements_in_groups = np.sum([len(group) for group in list_groups])
    flat_list = []
    for group in list_groups:
        flat_list.extend(group)
    unique_elements = np.unique(flat_list)
    n_unique_elements_in_groups = len(unique_elements)
    msg = f"One feature can only appear in one group, please check your grouped_features."
    assert n_unique_elements_in_groups == n_elements_in_groups, msg

    highest_feat = np.max(unique_elements)
    assert highest_feat < input_dim, f"Number of features is {input_dim} but one group contains {highest_feat}."  # noqa
    return


[docs]def filter_weights(weights):
    """
    This function makes sure that weights are in correct format for
    regression and multitask TabNet

    Parameters
    ----------
    weights : int, dict or list
        Initial weights parameters given by user

    Returns
    -------
    None : This function will only throw an error if format is wrong
    """
    err_msg = """Please provide a list or np.array of weights for """
    err_msg += """regression, multitask or pretraining: """
    if isinstance(weights, int):
        if weights == 1:
            raise ValueError(err_msg + "1 given.")
    if isinstance(weights, dict):
        raise ValueError(err_msg + "Dict given.")
    return


[docs]def validate_eval_set(eval_set, eval_name, X_train, y_train):
    """Check if the shapes of eval_set are compatible with (X_train, y_train).

    Parameters
    ----------
    eval_set : list of tuple
        List of eval tuple set (X, y).
        The last one is used for early stopping
    eval_name : list of str
        List of eval set names.
    X_train : np.ndarray
        Train owned products
    y_train : np.array
        Train targeted products

    Returns
    -------
    eval_names : list of str
        Validated list of eval_names.
    eval_set : list of tuple
        Validated list of eval_set.

    """
    eval_name = eval_name or [f"val_{i}" for i in range(len(eval_set))]

    assert len(eval_set) == len(
        eval_name
    ), "eval_set and eval_name have not the same length"
    if len(eval_set) > 0:
        assert all(
            len(elem) == 2 for elem in eval_set
        ), "Each tuple of eval_set need to have two elements"
    for name, (X, y) in zip(eval_name, eval_set):
        check_input(X)
        msg = (
            f"Dimension mismatch between X_{name} "
            + f"{X.shape} and X_train {X_train.shape}"
        )
        assert len(X.shape) == len(X_train.shape), msg

        msg = (
            f"Dimension mismatch between y_{name} "
            + f"{y.shape} and y_train {y_train.shape}"
        )
        assert len(y.shape) == len(y_train.shape), msg

        msg = (
            f"Number of columns is different between X_{name} "
            + f"({X.shape[1]}) and X_train ({X_train.shape[1]})"
        )
        assert X.shape[1] == X_train.shape[1], msg

        if len(y_train.shape) == 2:
            msg = (
                f"Number of columns is different between y_{name} "
                + f"({y.shape[1]}) and y_train ({y_train.shape[1]})"
            )
            assert y.shape[1] == y_train.shape[1], msg
        msg = (
            f"You need the same number of rows between X_{name} "
            + f"({X.shape[0]}) and y_{name} ({y.shape[0]})"
        )
        assert X.shape[0] == y.shape[0], msg

    return eval_name, eval_set


[docs]def define_device(device_name):
    """
    Define the device to use during training and inference.
    If auto it will detect automatically whether to use cuda or cpu

    Parameters
    ----------
    device_name : str
        Either "auto", "cpu" or "cuda"

    Returns
    -------
    str
        Either "cpu" or "cuda"
    """
    if device_name == "auto":
        if torch.cuda.is_available():
            return "cuda"
        else:
            return "cpu"
    elif device_name == "cuda" and not torch.cuda.is_available():
        return "cpu"
    else:
        return device_name


[docs]class ComplexEncoder(json.JSONEncoder):

[docs]    def default(self, obj):
        if isinstance(obj, (np.generic, np.ndarray)):
            return obj.tolist()
        # Let the base class default method raise the TypeError
        return json.JSONEncoder.default(self, obj)


[docs]def check_input(X):
    """
    Raise a clear error if X is a pandas dataframe
    and check array according to scikit rules
    """
    if isinstance(X, (pd.DataFrame, pd.Series)):
        err_message = "Pandas DataFrame are not supported: apply X.values when calling fit"
        raise TypeError(err_message)
    check_array(X, accept_sparse=True)


[docs]def check_warm_start(warm_start, from_unsupervised):
    """
    Gives a warning about ambiguous usage of the two parameters.
    """
    if warm_start and from_unsupervised is not None:
        warn_msg = "warm_start=True and from_unsupervised != None: "
        warn_msg = "warm_start will be ignore, training will start from unsupervised weights"
        warnings.warn(warn_msg)
    return


[docs]def check_embedding_parameters(cat_dims, cat_idxs, cat_emb_dim):
    """
    Check parameters related to embeddings and rearrange them in a unique manner.
    """
    if (cat_dims == []) ^ (cat_idxs == []):
        if cat_dims == []:
            msg = "If cat_idxs is non-empty, cat_dims must be defined as a list of same length."
        else:
            msg = "If cat_dims is non-empty, cat_idxs must be defined as a list of same length."
        raise ValueError(msg)
    elif len(cat_dims) != len(cat_idxs):
        msg = "The lists cat_dims and cat_idxs must have the same length."
        raise ValueError(msg)

    if isinstance(cat_emb_dim, int):
        cat_emb_dims = [cat_emb_dim] * len(cat_idxs)
    else:
        cat_emb_dims = cat_emb_dim

    # check that all embeddings are provided
    if len(cat_emb_dims) != len(cat_dims):
        msg = f"""cat_emb_dim and cat_dims must be lists of same length, got {len(cat_emb_dims)}
                    and {len(cat_dims)}"""
        raise ValueError(msg)

    # Rearrange to get reproducible seeds with different ordering
    if len(cat_idxs) > 0:
        sorted_idxs = np.argsort(cat_idxs)
        cat_dims = [cat_dims[i] for i in sorted_idxs]
        cat_emb_dims = [cat_emb_dims[i] for i in sorted_idxs]

    return cat_dims, cat_idxs, cat_emb_dims
```

---

© Copyright 2019, Dreamquark

Built with Sphinx using a
theme
provided by Read the Docs.
