# Supplementary material for: Development of a deep learning model for predicting recurrence of hepatocellular carcinoma after liver transplantation
Source: Front Med (Lausanne). 2024 Jun 11;11:1373005. doi: 10.3389/fmed.2024.1373005 (PMC11196752; doi:10.3389/fmed.2024.1373005)
Supplement: Supplementary file 1 [file Data_Sheet_1.ZIP › Raw data/source data and codes/codes/tabnet/docs/_modules/torch/optim/adam.html]

torch.optim.adam — pytorch\_tabnet documentation


pytorch\_tabnet

Contents:

- README
- TabNet : Attentive Interpretable Tabular Learning
- Installation
- What is new ?
- Contributing
- What problems does pytorch-tabnet handle?
- How to use it?
- Semi-supervised pre-training
- Data augmentation on the fly
- Easy saving and loading
- Useful links
- pytorch\_tabnet package

pytorch\_tabnet

- »
- Module code »
- torch.optim.adam

---

# Source code for torch.optim.adam

```
from collections import defaultdict
import math
from typing import cast, List, Optional, Dict, Tuple

import torch
from torch import Tensor
from .optimizer import Optimizer, _use_grad_for_differentiable

__all__ = ['Adam', 'adam']


# TODO(crcrpar): Move this to soemwhere (e.g. torch/optim/_utils?) else when adding another fused optimizer.
# NOTE(crcrpar): Almost the same as `_MultiDeviceReplicator` defined in
# torch/cuda/amp/grad_scaler.py except for the key being str only for torch script.
class _MultiDeviceReplicator:
    main_tensor: Tensor
    _per_device_tensors: Dict[str, Tensor]

    def __init__(self, main_tensor: Tensor) -> None:
        self.main_tensor = main_tensor
        self._per_device_tensors = {str(main_tensor.device): main_tensor}

    def get(self, device: str):
        if device in self._per_device_tensors:
            return self._per_device_tensors[device]
        tensor = self.main_tensor.to(device=device, non_blocking=True, copy=True)
        self._per_device_tensors[device] = tensor
        return tensor


# todo(crcrpar): Move this to another place when adding another fused optimizer.
def _get_fp16AMP_params(
    *,
    optimizer: Optimizer,
    grad_scaler: Optional[torch.cuda.amp.GradScaler] = None,
    device: torch.device,
) -> Optional[_MultiDeviceReplicator]:
    if grad_scaler is None:
        return None
    found_inf_dict = grad_scaler._check_inf_per_device(optimizer)
    # Combines found_inf tensors from all devices. As in GradScaler.update(),
    # tensors are combined on the scale's device, which is an arbitrary but
    # reasonable choice that avoids new context creation.
    found_infs = [f.to(device, non_blocking=True) for f in found_inf_dict.values()]
    assert len(found_infs) > 0, "No inf checks were recorded in _check_inf_per_device."
    with torch.no_grad():
        found_inf_combined = cast(torch.Tensor, sum(found_infs))
    return _MultiDeviceReplicator(found_inf_combined)

class Adam(Optimizer):
    r"""Implements Adam algorithm.

    .. math::
       \begin{aligned}
            &\rule{110mm}{0.4pt}                                                                 \\
            &\textbf{input}      : \gamma \text{ (lr)}, \beta_1, \beta_2
                \text{ (betas)},\theta_0 \text{ (params)},f(\theta) \text{ (objective)}          \\
            &\hspace{13mm}      \lambda \text{ (weight decay)},  \: \textit{amsgrad},
                \:\textit{maximize}                                                              \\
            &\textbf{initialize} :  m_0 \leftarrow 0 \text{ ( first moment)},
                v_0\leftarrow 0 \text{ (second moment)},\: \widehat{v_0}^{max}\leftarrow 0\\[-1.ex]
            &\rule{110mm}{0.4pt}                                                                 \\
            &\textbf{for} \: t=1 \: \textbf{to} \: \ldots \: \textbf{do}                         \\

            &\hspace{5mm}\textbf{if} \: \textit{maximize}:                                       \\
            &\hspace{10mm}g_t           \leftarrow   -\nabla_{\theta} f_t (\theta_{t-1})         \\
            &\hspace{5mm}\textbf{else}                                                           \\
            &\hspace{10mm}g_t           \leftarrow   \nabla_{\theta} f_t (\theta_{t-1})          \\
            &\hspace{5mm}\textbf{if} \: \lambda \neq 0                                           \\
            &\hspace{10mm} g_t \leftarrow g_t + \lambda  \theta_{t-1}                            \\
            &\hspace{5mm}m_t           \leftarrow   \beta_1 m_{t-1} + (1 - \beta_1) g_t          \\
            &\hspace{5mm}v_t           \leftarrow   \beta_2 v_{t-1} + (1-\beta_2) g^2_t          \\
            &\hspace{5mm}\widehat{m_t} \leftarrow   m_t/\big(1-\beta_1^t \big)                   \\
            &\hspace{5mm}\widehat{v_t} \leftarrow   v_t/\big(1-\beta_2^t \big)                   \\
            &\hspace{5mm}\textbf{if} \: amsgrad                                                  \\
            &\hspace{10mm}\widehat{v_t}^{max} \leftarrow \mathrm{max}(\widehat{v_t}^{max},
                \widehat{v_t})                                                                   \\
            &\hspace{10mm}\theta_t \leftarrow \theta_{t-1} - \gamma \widehat{m_t}/
                \big(\sqrt{\widehat{v_t}^{max}} + \epsilon \big)                                 \\
            &\hspace{5mm}\textbf{else}                                                           \\
            &\hspace{10mm}\theta_t \leftarrow \theta_{t-1} - \gamma \widehat{m_t}/
                \big(\sqrt{\widehat{v_t}} + \epsilon \big)                                       \\
            &\rule{110mm}{0.4pt}                                                          \\[-1.ex]
            &\bf{return} \:  \theta_t                                                     \\[-1.ex]
            &\rule{110mm}{0.4pt}                                                          \\[-1.ex]
       \end{aligned}

    For further details regarding the algorithm we refer to `Adam: A Method for Stochastic Optimization`_.

    Args:
        params (iterable): iterable of parameters to optimize or dicts defining
            parameter groups
        lr (float, optional): learning rate (default: 1e-3)
        betas (Tuple[float, float], optional): coefficients used for computing
            running averages of gradient and its square (default: (0.9, 0.999))
        eps (float, optional): term added to the denominator to improve
            numerical stability (default: 1e-8)
        weight_decay (float, optional): weight decay (L2 penalty) (default: 0)
        amsgrad (bool, optional): whether to use the AMSGrad variant of this
            algorithm from the paper `On the Convergence of Adam and Beyond`_
            (default: False)
        foreach (bool, optional): whether foreach implementation of optimizer
            is used (default: None)
        maximize (bool, optional): maximize the params based on the objective, instead of
            minimizing (default: False)
        capturable (bool, optional): whether this instance is safe to capture in a CUDA graph.
            Passing True can impair ungraphed performance, so if you don't intend to
            graph capture this instance, leave it False (default: False)
        fused (bool, optional): whether fused implementation of optimizer is used.
            Currently, `torch.float64`, `torch.float32`, `torch.float16`, and `torch.bfloat16`
            are supported. (default: False)

    .. _Adam\: A Method for Stochastic Optimization:
        https://arxiv.org/abs/1412.6980
    .. _On the Convergence of Adam and Beyond:
        https://openreview.net/forum?id=ryQu7f-RZ
    """

    def __init__(self, params, lr=1e-3, betas=(0.9, 0.999), eps=1e-8,
                 weight_decay=0, amsgrad=False, *, foreach: Optional[bool] = None,
                 maximize: bool = False, capturable: bool = False,
                 differentiable: bool = False, fused: bool = False):
        if not 0.0 <= lr:
            raise ValueError("Invalid learning rate: {}".format(lr))
        if not 0.0 <= eps:
            raise ValueError("Invalid epsilon value: {}".format(eps))
        if not 0.0 <= betas[0] < 1.0:
            raise ValueError("Invalid beta parameter at index 0: {}".format(betas[0]))
        if not 0.0 <= betas[1] < 1.0:
            raise ValueError("Invalid beta parameter at index 1: {}".format(betas[1]))
        if not 0.0 <= weight_decay:
            raise ValueError("Invalid weight_decay value: {}".format(weight_decay))
        defaults = dict(lr=lr, betas=betas, eps=eps,
                        weight_decay=weight_decay, amsgrad=amsgrad,
                        maximize=maximize, foreach=foreach, capturable=capturable,
                        differentiable=differentiable, fused=fused)
        super(Adam, self).__init__(params, defaults)

        if fused:
            if differentiable:
                raise RuntimeError("`fused` cannot be `differentiable`")
            self._step_supports_amp_scaling = True
            # TODO(crcrpar): [low prec params & their higher prec copy]
            # Suppor AMP with FP16/BF16 model params which would need
            # higher prec copy of params to do update math in higher prec to
            # alleviate the loss of information.
            if not all(
                p.is_cuda and torch.is_floating_point(p)
                for pg in self.param_groups for p in pg['params']
            ):
                raise RuntimeError("FusedAdam requires all the params to be CUDA, floating point")

    def __setstate__(self, state):
        super().__setstate__(state)
        for group in self.param_groups:
            group.setdefault('amsgrad', False)
            group.setdefault('maximize', False)
            group.setdefault('foreach', None)
            group.setdefault('capturable', False)
            group.setdefault('differentiable', False)
            group.setdefault('fused', False)
        state_values = list(self.state.values())
        step_is_tensor = (len(state_values) != 0) and torch.is_tensor(state_values[0]['step'])
        if not step_is_tensor:
            for s in state_values:
                s['step'] = torch.tensor(float(s['step']))

    @_use_grad_for_differentiable
    def step(self, closure=None, *, grad_scaler=None):
        """Performs a single optimization step.

        Args:
            closure (Callable, optional): A closure that reevaluates the model
                and returns the loss.
            grad_scaler (:class:`torch.cuda.amp.GradScaler`, optional): A GradScaler which is
                supplied from ``grad_scaler.step(optimizer)``.
        """
        self._cuda_graph_capture_health_check()

        loss = None
        if closure is not None:
            with torch.enable_grad():
                loss = closure()

        for group in self.param_groups:
            params_with_grad = []
            grads = []
            exp_avgs = []
            exp_avg_sqs = []
            max_exp_avg_sqs = []
            state_steps = []
            beta1, beta2 = group['betas']

            grad_scale = None
            found_inf = None
            if group['fused'] and grad_scaler is not None:
                grad_scale = grad_scaler._get_scale_async()
                device = grad_scale.device
                grad_scale = _MultiDeviceReplicator(grad_scale)
                found_inf = _get_fp16AMP_params(optimizer=self, grad_scaler=grad_scaler, device=device)

            for p in group['params']:
                if p.grad is not None:
                    params_with_grad.append(p)
                    if p.grad.is_sparse:
                        raise RuntimeError('Adam does not support sparse gradients, please consider SparseAdam instead')
                    grads.append(p.grad)

                    state = self.state[p]
                    # Lazy state initialization
                    if len(state) == 0:
                        state['step'] = (
                            torch.zeros((1,), dtype=torch.float, device=p.device)
                            if self.defaults['capturable'] or self.defaults['fused']
                            else torch.tensor(0.)
                        )
                        # Exponential moving average of gradient values
                        state['exp_avg'] = torch.zeros_like(p, memory_format=torch.preserve_format)
                        # Exponential moving average of squared gradient values
                        state['exp_avg_sq'] = torch.zeros_like(p, memory_format=torch.preserve_format)
                        if group['amsgrad']:
                            # Maintains max of all exp. moving avg. of sq. grad. values
                            state['max_exp_avg_sq'] = torch.zeros_like(p, memory_format=torch.preserve_format)

                    exp_avgs.append(state['exp_avg'])
                    exp_avg_sqs.append(state['exp_avg_sq'])

                    if group['amsgrad']:
                        max_exp_avg_sqs.append(state['max_exp_avg_sq'])
                    if group['differentiable'] and state['step'].requires_grad:
                        raise RuntimeError('`requires_grad` is not supported for `step` in differentiable mode')
                    state_steps.append(state['step'])

            adam(params_with_grad,
                 grads,
                 exp_avgs,
                 exp_avg_sqs,
                 max_exp_avg_sqs,
                 state_steps,
                 amsgrad=group['amsgrad'],
                 beta1=beta1,
                 beta2=beta2,
                 lr=group['lr'],
                 weight_decay=group['weight_decay'],
                 eps=group['eps'],
                 maximize=group['maximize'],
                 foreach=group['foreach'],
                 capturable=group['capturable'],
                 differentiable=group['differentiable'],
                 fused=group['fused'],
                 grad_scale=grad_scale,
                 found_inf=found_inf)

        return loss


def adam(params: List[Tensor],
         grads: List[Tensor],
         exp_avgs: List[Tensor],
         exp_avg_sqs: List[Tensor],
         max_exp_avg_sqs: List[Tensor],
         state_steps: List[Tensor],
         # kwonly args with defaults are not supported by functions compiled with torchscript issue #70627
         # setting this as kwarg for now as functional API is compiled by torch/distributed/optim
         foreach: Optional[bool] = None,
         capturable: bool = False,
         differentiable: bool = False,
         fused: bool = False,
         grad_scale: Optional[_MultiDeviceReplicator] = None,
         found_inf: Optional[_MultiDeviceReplicator] = None,
         *,
         amsgrad: bool,
         beta1: float,
         beta2: float,
         lr: float,
         weight_decay: float,
         eps: float,
         maximize: bool):
    r"""Functional API that performs Adam algorithm computation.
    See :class:`~torch.optim.Adam` for details.
    """

    if not all(isinstance(t, torch.Tensor) for t in state_steps):
        raise RuntimeError("API has changed, `state_steps` argument must contain a list of singleton tensors")

    if foreach is None:
        # Placeholder for more complex foreach logic to be added when value is not set
        foreach = False

    if foreach and torch.jit.is_scripting():
        raise RuntimeError('torch.jit.script not supported with foreach optimizers')

    if foreach and not torch.jit.is_scripting():
        func = _multi_tensor_adam
    elif fused and not torch.jit.is_scripting():
        func = _fused_adam
    else:
        func = _single_tensor_adam

    func(params,
         grads,
         exp_avgs,
         exp_avg_sqs,
         max_exp_avg_sqs,
         state_steps,
         amsgrad=amsgrad,
         beta1=beta1,
         beta2=beta2,
         lr=lr,
         weight_decay=weight_decay,
         eps=eps,
         maximize=maximize,
         capturable=capturable,
         differentiable=differentiable,
         grad_scale=grad_scale,
         found_inf=found_inf)


def _single_tensor_adam(params: List[Tensor],
                        grads: List[Tensor],
                        exp_avgs: List[Tensor],
                        exp_avg_sqs: List[Tensor],
                        max_exp_avg_sqs: List[Tensor],
                        state_steps: List[Tensor],
                        grad_scale: Optional[_MultiDeviceReplicator],
                        found_inf: Optional[_MultiDeviceReplicator],
                        *,
                        amsgrad: bool,
                        beta1: float,
                        beta2: float,
                        lr: float,
                        weight_decay: float,
                        eps: float,
                        maximize: bool,
                        capturable: bool,
                        differentiable: bool):

    assert grad_scale is None and found_inf is None

    for i, param in enumerate(params):

        grad = grads[i] if not maximize else -grads[i]
        exp_avg = exp_avgs[i]
        exp_avg_sq = exp_avg_sqs[i]
        step_t = state_steps[i]

        if capturable:
            assert param.is_cuda and step_t.is_cuda, "If capturable=True, params and state_steps must be CUDA tensors."

        # update step
        step_t += 1

        if weight_decay != 0:
            grad = grad.add(param, alpha=weight_decay)

        if torch.is_complex(param):
            grad = torch.view_as_real(grad)
            exp_avg = torch.view_as_real(exp_avg)
            exp_avg_sq = torch.view_as_real(exp_avg_sq)
            param = torch.view_as_real(param)

        # Decay the first and second moment running average coefficient
        exp_avg.mul_(beta1).add_(grad, alpha=1 - beta1)
        exp_avg_sq.mul_(beta2).addcmul_(grad, grad.conj(), value=1 - beta2)

        if capturable or differentiable:
            step = step_t

            # 1 - beta1 ** step can't be captured in a CUDA graph, even if step is a CUDA tensor
            # (incurs "RuntimeError: CUDA error: operation not permitted when stream is capturing")
            bias_correction1 = 1 - torch.pow(beta1, step)
            bias_correction2 = 1 - torch.pow(beta2, step)

            step_size = lr / bias_correction1
            step_size_neg = step_size.neg()

            bias_correction2_sqrt = bias_correction2.sqrt()

            if amsgrad:
                # Maintains the maximum of all 2nd moment running avg. till now
                if differentiable:
                    max_exp_avg_sqs_i = max_exp_avg_sqs[i].clone()
                else:
                    max_exp_avg_sqs_i = max_exp_avg_sqs[i]
                max_exp_avg_sqs[i].copy_(torch.maximum(max_exp_avg_sqs_i, exp_avg_sq))
                # Uses the max. for normalizing running avg. of gradient
                # Folds in (admittedly ugly) 1-elem step_size math here to avoid extra param-set-sized read+write
                # (can't fold it into addcdiv_ below because addcdiv_ requires value is a Number, not a Tensor)
                denom = (max_exp_avg_sqs[i].sqrt() / (bias_correction2_sqrt * step_size_neg)).add_(eps / step_size_neg)
            else:
                denom = (exp_avg_sq.sqrt() / (bias_correction2_sqrt * step_size_neg)).add_(eps / step_size_neg)

            param.addcdiv_(exp_avg, denom)
        else:
            step = step_t.item()

            bias_correction1 = 1 - beta1 ** step
            bias_correction2 = 1 - beta2 ** step

            step_size = lr / bias_correction1

            bias_correction2_sqrt = math.sqrt(bias_correction2)

            if amsgrad:
                # Maintains the maximum of all 2nd moment running avg. till now
                torch.maximum(max_exp_avg_sqs[i], exp_avg_sq, out=max_exp_avg_sqs[i])
                # Use the max. for normalizing running avg. of gradient
                denom = (max_exp_avg_sqs[i].sqrt() / bias_correction2_sqrt).add_(eps)
            else:
                denom = (exp_avg_sq.sqrt() / bias_correction2_sqrt).add_(eps)

            param.addcdiv_(exp_avg, denom, value=-step_size)


def _multi_tensor_adam(params: List[Tensor],
                       grads: List[Tensor],
                       exp_avgs: List[Tensor],
                       exp_avg_sqs: List[Tensor],
                       max_exp_avg_sqs: List[Tensor],
                       state_steps: List[Tensor],
                       grad_scale: Optional[_MultiDeviceReplicator],
                       found_inf: Optional[_MultiDeviceReplicator],
                       *,
                       amsgrad: bool,
                       beta1: float,
                       beta2: float,
                       lr: float,
                       weight_decay: float,
                       eps: float,
                       maximize: bool,
                       capturable: bool,
                       differentiable: bool):
    if len(params) == 0:
        return

    if capturable:
        assert all(p.is_cuda and step.is_cuda for p, step in zip(params, state_steps)), \
            "If capturable=True, params and state_steps must be CUDA tensors."

    assert grad_scale is None and found_inf is None

    if maximize:
        grads = torch._foreach_neg(tuple(grads))  # type: ignore[assignment]

    assert not differentiable, "_foreach ops don't support autograd"
    # Handle complex parameters
    grads = [torch.view_as_real(x) if torch.is_complex(x) else x for x in grads]
    exp_avgs = [torch.view_as_real(x) if torch.is_complex(x) else x for x in exp_avgs]
    exp_avg_sqs = [torch.view_as_real(x) if torch.is_complex(x) else x for x in exp_avg_sqs]
    params_ = [torch.view_as_real(x) if torch.is_complex(x) else x for x in params]

    # update steps
    torch._foreach_add_(state_steps, 1)

    if weight_decay != 0:
        torch._foreach_add_(grads, params, alpha=weight_decay)

    # Decay the first and second moment running average coefficient
    torch._foreach_mul_(exp_avgs, beta1)
    torch._foreach_add_(exp_avgs, grads, alpha=1 - beta1)

    torch._foreach_mul_(exp_avg_sqs, beta2)
    torch._foreach_addcmul_(exp_avg_sqs, grads, grads, 1 - beta2)

    if capturable:
        # TODO: use foreach_pow if/when foreach_pow is added
        bias_correction1 = [torch.pow(beta1, step) for step in state_steps]
        bias_correction2 = [torch.pow(beta2, step) for step in state_steps]
        # foreach_sub doesn't allow a scalar as the first arg
        torch._foreach_sub_(bias_correction1, 1)
        torch._foreach_sub_(bias_correction2, 1)
        torch._foreach_neg_(bias_correction1)
        torch._foreach_neg_(bias_correction2)

        # foreach_div doesn't allow a scalar as the first arg
        step_size = torch._foreach_div(bias_correction1, lr)
        torch._foreach_reciprocal_(step_size)
        torch._foreach_neg_(step_size)

        bias_correction2_sqrt = torch._foreach_sqrt(bias_correction2)

        if amsgrad:
            # Maintains the maximum of all 2nd moment running avg. till now
            torch._foreach_maximum_(max_exp_avg_sqs, exp_avg_sqs)  # type: ignore[assignment]

            # Use the max. for normalizing running avg. of gradient
            max_exp_avg_sq_sqrt = torch._foreach_sqrt(max_exp_avg_sqs)
            # Folds in (admittedly ugly) 1-elem step_size math here to avoid extra param-set-sized read+write
            # (can't fold it into addcdiv_ below because addcdiv_ requires value is a Number, not a Tensor)
            torch._foreach_div_(max_exp_avg_sq_sqrt, torch._foreach_mul(bias_correction2_sqrt, step_size))
            eps_over_step_size = torch._foreach_div(step_size, eps)
            torch._foreach_reciprocal_(eps_over_step_size)
            denom = torch._foreach_add(max_exp_avg_sq_sqrt, eps_over_step_size)
        else:
            exp_avg_sq_sqrt = torch._foreach_sqrt(exp_avg_sqs)
            torch._foreach_div_(exp_avg_sq_sqrt, torch._foreach_mul(bias_correction2_sqrt, step_size))
            eps_over_step_size = torch._foreach_div(step_size, eps)
            torch._foreach_reciprocal_(eps_over_step_size)
            denom = torch._foreach_add(exp_avg_sq_sqrt, eps_over_step_size)

        torch._foreach_addcdiv_(params_, exp_avgs, denom)
    else:
        bias_correction1 = [1 - beta1 ** step.item() for step in state_steps]
        bias_correction2 = [1 - beta2 ** step.item() for step in state_steps]

        step_size = [(lr / bc) * -1 for bc in bias_correction1]

        bias_correction2_sqrt = [math.sqrt(bc) for bc in bias_correction2]

        if amsgrad:
            # Maintains the maximum of all 2nd moment running avg. till now
            torch._foreach_maximum_(max_exp_avg_sqs, exp_avg_sqs)

            # Use the max. for normalizing running avg. of gradient
            max_exp_avg_sq_sqrt = torch._foreach_sqrt(max_exp_avg_sqs)
            torch._foreach_div_(max_exp_avg_sq_sqrt, bias_correction2_sqrt)
            denom = torch._foreach_add(max_exp_avg_sq_sqrt, eps)
        else:
            exp_avg_sq_sqrt = torch._foreach_sqrt(exp_avg_sqs)
            torch._foreach_div_(exp_avg_sq_sqrt, bias_correction2_sqrt)
            denom = torch._foreach_add(exp_avg_sq_sqrt, eps)

        torch._foreach_addcdiv_(params_, exp_avgs, denom, step_size)


# TODO(crcrpar): Move this to another place when adding another fused optimizer.
# TODO(crcrpar): Make this generic when there's more fused optimizers.
# TODO(crcrpar): Think of rewriting this in C++.
@torch.no_grad()
def _group_params_by_device_and_dtype(
    params: List[Tensor],
    grads: List[Tensor],
    exp_avgs: List[Tensor],
    exp_avg_sqs: List[Tensor],
    max_exp_avg_sqs: List[Tensor],
    state_steps: List[Tensor],
) -> Dict[Tuple[str, torch.dtype], List[List[Tensor]]]:
    per_device_and_dtype_tensors = defaultdict(lambda: [[] for _ in range(6)])
    for i, (p, step) in enumerate(zip(params, state_steps)):
        key = (str(p.device), p.dtype)
        per_device_and_dtype_tensors[key][0].append(p)
        per_device_and_dtype_tensors[key][1].append(grads[i])
        per_device_and_dtype_tensors[key][2].append(exp_avgs[i])
        per_device_and_dtype_tensors[key][3].append(exp_avg_sqs[i])
        if max_exp_avg_sqs:
            per_device_and_dtype_tensors[key][4].append(max_exp_avg_sqs[i])
        per_device_and_dtype_tensors[key][5].append(step)
    return per_device_and_dtype_tensors


def _fused_adam(
    params: List[Tensor],
    grads: List[Tensor],
    exp_avgs: List[Tensor],
    exp_avg_sqs: List[Tensor],
    max_exp_avg_sqs: List[Tensor],
    state_steps: List[Tensor],
    grad_scale: Optional[_MultiDeviceReplicator],
    found_inf: Optional[_MultiDeviceReplicator],
    *,
    amsgrad: bool,
    beta1: float,
    beta2: float,
    lr: float,
    weight_decay: float,
    eps: float,
    maximize: bool,
    capturable: bool,  # Needed for consistency.
    differentiable: bool,
) -> None:
    grouped_tensors = _group_params_by_device_and_dtype(params, grads, exp_avgs, exp_avg_sqs, max_exp_avg_sqs, state_steps)
    for (device, dtype) in grouped_tensors:
        (
            device_params,
            device_grads,
            device_exp_avgs,
            device_exp_avg_sqs,
            device_max_exp_avg_sqs,
            device_state_steps,
        ) = grouped_tensors[(device, dtype)]
        if grad_scale is not None and found_inf is not None:
            device_grad_scale = grad_scale.get(device)
            device_found_inf = found_inf.get(device)
        else:
            device_grad_scale = None
            device_found_inf = None
        torch._foreach_add_(device_state_steps, 1)
        torch._fused_adam_(
            device_params,
            device_grads,
            device_exp_avgs,
            device_exp_avg_sqs,
            device_max_exp_avg_sqs,
            device_state_steps,
            amsgrad=amsgrad,
            lr=lr,
            beta1=beta1,
            beta2=beta2,
            weight_decay=weight_decay,
            eps=eps,
            maximize=maximize,
            grad_scale=device_grad_scale,
            found_inf=device_found_inf,
        )
        if device_found_inf is not None:
            torch._foreach_sub_(device_state_steps, [device_found_inf] * len(device_state_steps))
```

---

© Copyright 2019, Dreamquark

Built with Sphinx using a
theme
provided by Read the Docs.
