# Supplementary material for: Development of a deep learning model for predicting recurrence of hepatocellular carcinoma after liver transplantation
Source: Front Med (Lausanne). 2024 Jun 11;11:1373005. doi: 10.3389/fmed.2024.1373005 (PMC11196752; doi:10.3389/fmed.2024.1373005)
Supplement: Supplementary file 1 [file Data_Sheet_1.ZIP › Raw data/source data and codes/codes/tabnet/docs/generated_docs/docs-scripts....pytorch_tabnet.html]

docs-scripts….pytorch\_tabnet package — pytorch\_tabnet documentation


pytorch\_tabnet

Contents:

- README
- TabNet : Attentive Interpretable Tabular Learning
- Installation
- What problems does pytorch-tabnet handles?
- How to use it?
- Useful links

pytorch\_tabnet

- »
- docs-scripts….pytorch\_tabnet package
- View page source

---

# docs-scripts….pytorch\_tabnet package¶

## docs-scripts….pytorch\_tabnet.metrics module¶

## docs-scripts….pytorch\_tabnet.sparsemax module¶

## docs-scripts….pytorch\_tabnet.callbacks module¶

## docs-scripts….pytorch\_tabnet.tab\_network module¶

## docs-scripts….pytorch\_tabnet.utils module¶

## docs-scripts….pytorch\_tabnet.multiclass\_utils module¶

## docs-scripts….pytorch\_tabnet.abstract\_model module¶

## docs-scripts….pytorch\_tabnet.multitask module¶

## docs-scripts….pytorch\_tabnet.tab\_model module¶

---

© Copyright 2019, Dreamquark

Built with Sphinx using a
theme
provided by Read the Docs.
