# Supplementary material for: Development of a deep learning model for predicting recurrence of hepatocellular carcinoma after liver transplantation
Source: Front Med (Lausanne). 2024 Jun 11;11:1373005. doi: 10.3389/fmed.2024.1373005 (PMC11196752; doi:10.3389/fmed.2024.1373005)
Supplement: Supplementary file 1 [file Data_Sheet_1.ZIP › Raw data/source data and codes/codes/tabnet/docs/generated_docs/pytorch_tabnet.html]

pytorch\_tabnet package — pytorch\_tabnet documentation


pytorch\_tabnet

Contents:

- README
- TabNet : Attentive Interpretable Tabular Learning
- Installation
- What is new ?
- Contributing
- What problems does pytorch-tabnet handle?
- How to use it?
- Semi-supervised pre-training
- Data augmentation on the fly
- Easy saving and loading
- Useful links
- pytorch\_tabnet package
  - pytorch\_tabnet.pretraining\_utils module
  - pytorch\_tabnet.augmentations module
  - pytorch\_tabnet.tab\_network module
  - pytorch\_tabnet.metrics module
  - pytorch\_tabnet.tab\_model module
  - pytorch\_tabnet.sparsemax module
  - pytorch\_tabnet.callbacks module
  - pytorch\_tabnet.abstract\_model module
  - pytorch\_tabnet.pretraining module
  - pytorch\_tabnet.utils module
  - pytorch\_tabnet.multitask module
  - pytorch\_tabnet.multiclass\_utils module
    - Multi-class / multi-label utility function

pytorch\_tabnet

- »
- pytorch\_tabnet package
- View page source

---

# pytorch\_tabnet package¶

## pytorch\_tabnet.pretraining\_utils module¶

`pytorch_tabnet.pretraining_utils.``create_dataloaders`(*X\_train*, *eval\_set*, *weights*, *batch\_size*, *num\_workers*, *drop\_last*, *pin\_memory*)[source]¶
:   Create dataloaders with or without subsampling depending on weights and balanced.

    Parameters
    :   - **X\_train** (*np.ndarray* *or* *scipy.sparse.csr\_matrix*) – Training data
        - **eval\_set** (*list of np.array* *(**for Xs and ys**) or* *scipy.sparse.csr\_matrix* *(**for Xs**)*) – List of eval sets
        - **weights** (*either 0**,* *1**,* *dict* *or* *iterable*) –

          if 0 (default) : no weights will be applied
          if 1 : classification only, will balanced class with inverse frequency
          if dict : keys are corresponding class values are sample weights
          if iterable : list or np array must be of length equal to nb elements

          > in the training set
        - **batch\_size** (*int*) – how many samples per batch to load
        - **num\_workers** (*int*) – how many subprocesses to use for data loading. 0 means that the data
          will be loaded in the main process
        - **drop\_last** (*bool*) – set to True to drop the last incomplete batch, if the dataset size is not
          divisible by the batch size. If False and the size of dataset is not
          divisible by the batch size, then the last batch will be smaller
        - **pin\_memory** (*bool*) – Whether to pin GPU memory during training

    Returns
    :   **train\_dataloader, valid\_dataloader** – Training and validation dataloaders

    Return type
    :   torch.DataLoader, torch.DataLoader

`pytorch_tabnet.pretraining_utils.``validate_eval_set`(*eval\_set*, *eval\_name*, *X\_train*)[source]¶
:   Check if the shapes of eval\_set are compatible with X\_train.

    Parameters
    :   - **eval\_set** (*List of numpy array*) – The list evaluation set.
          The last one is used for early stopping
        - **X\_train** (*np.ndarray*) – Train owned products

    Returns
    :   **eval\_names** – Validated list of eval\_names.

    Return type
    :   list of str

## pytorch\_tabnet.augmentations module¶

*class* `pytorch_tabnet.augmentations.``ClassificationSMOTE`(*device\_name='auto'*, *p=0.8*, *alpha=0.5*, *beta=0.5*, *seed=0*)[source]¶
:   Bases: `object`

    Apply SMOTE for classification tasks.

    This will average a percentage p of the elements in the batch with other elements.
    The target will stay unchanged and keep the value of the most important row in the mix.

*class* `pytorch_tabnet.augmentations.``RegressionSMOTE`(*device\_name='auto'*, *p=0.8*, *alpha=0.5*, *beta=0.5*, *seed=0*)[source]¶
:   Bases: `object`

    Apply SMOTE

    This will average a percentage p of the elements in the batch with other elements.
    The target will be averaged as well (this might work with binary classification
    and certain loss), following a beta distribution.

## pytorch\_tabnet.tab\_network module¶

*class* `pytorch_tabnet.tab_network.``AttentiveTransformer`(*input\_dim*, *group\_dim*, *group\_matrix*, *virtual\_batch\_size=128*, *momentum=0.02*, *mask\_type='sparsemax'*)[source]¶
:   Bases: `torch.nn.modules.module.Module`

    `forward`(*priors*, *processed\_feat*)[source]¶
    :   Defines the computation performed at every call.

        Should be overridden by all subclasses.

        Note

        Although the recipe for forward pass needs to be defined within
        this function, one should call the `Module` instance afterwards
        instead of this since the former takes care of running the
        registered hooks while the latter silently ignores them.

    `training` *= None*¶

*class* `pytorch_tabnet.tab_network.``EmbeddingGenerator`(*input\_dim*, *cat\_dims*, *cat\_idxs*, *cat\_emb\_dims*, *group\_matrix*)[source]¶
:   Bases: `torch.nn.modules.module.Module`

    Classical embeddings generator

    `forward`(*x*)[source]¶
    :   Apply embeddings to inputs
        Inputs should be (batch\_size, input\_dim)
        Outputs will be of size (batch\_size, self.post\_embed\_dim)

    `training` *= None*¶

*class* `pytorch_tabnet.tab_network.``FeatTransformer`(*input\_dim*, *output\_dim*, *shared\_layers*, *n\_glu\_independent*, *virtual\_batch\_size=128*, *momentum=0.02*)[source]¶
:   Bases: `torch.nn.modules.module.Module`

    `forward`(*x*)[source]¶
    :   Defines the computation performed at every call.

        Should be overridden by all subclasses.

        Note

        Although the recipe for forward pass needs to be defined within
        this function, one should call the `Module` instance afterwards
        instead of this since the former takes care of running the
        registered hooks while the latter silently ignores them.

    `training` *= None*¶

*class* `pytorch_tabnet.tab_network.``GBN`(*input\_dim*, *virtual\_batch\_size=128*, *momentum=0.01*)[source]¶
:   Bases: `torch.nn.modules.module.Module`

    Ghost Batch Normalization
    https://arxiv.org/abs/1705.08741

    `forward`(*x*)[source]¶
    :   Defines the computation performed at every call.

        Should be overridden by all subclasses.

        Note

        Although the recipe for forward pass needs to be defined within
        this function, one should call the `Module` instance afterwards
        instead of this since the former takes care of running the
        registered hooks while the latter silently ignores them.

    `training` *= None*¶

*class* `pytorch_tabnet.tab_network.``GLU_Block`(*input\_dim*, *output\_dim*, *n\_glu=2*, *first=False*, *shared\_layers=None*, *virtual\_batch\_size=128*, *momentum=0.02*)[source]¶
:   Bases: `torch.nn.modules.module.Module`

    Independent GLU block, specific to each step

    `forward`(*x*)[source]¶
    :   Defines the computation performed at every call.

        Should be overridden by all subclasses.

        Note

        Although the recipe for forward pass needs to be defined within
        this function, one should call the `Module` instance afterwards
        instead of this since the former takes care of running the
        registered hooks while the latter silently ignores them.

    `training` *= None*¶

*class* `pytorch_tabnet.tab_network.``GLU_Layer`(*input\_dim*, *output\_dim*, *fc=None*, *virtual\_batch\_size=128*, *momentum=0.02*)[source]¶
:   Bases: `torch.nn.modules.module.Module`

    `forward`(*x*)[source]¶
    :   Defines the computation performed at every call.

        Should be overridden by all subclasses.

        Note

        Although the recipe for forward pass needs to be defined within
        this function, one should call the `Module` instance afterwards
        instead of this since the former takes care of running the
        registered hooks while the latter silently ignores them.

    `training` *= None*¶

*class* `pytorch_tabnet.tab_network.``RandomObfuscator`(*pretraining\_ratio*, *group\_matrix*)[source]¶
:   Bases: `torch.nn.modules.module.Module`

    Create and applies obfuscation masks.
    The obfuscation is done at group level to match attention.

    `forward`(*x*)[source]¶
    :   Generate random obfuscation mask.

        Returns


        Return type
        :   masked input and obfuscated variables.

    `training` *= None*¶

*class* `pytorch_tabnet.tab_network.``TabNet`(*input\_dim*, *output\_dim*, *n\_d=8*, *n\_a=8*, *n\_steps=3*, *gamma=1.3*, *cat\_idxs=[]*, *cat\_dims=[]*, *cat\_emb\_dim=1*, *n\_independent=2*, *n\_shared=2*, *epsilon=1e-15*, *virtual\_batch\_size=128*, *momentum=0.02*, *mask\_type='sparsemax'*, *group\_attention\_matrix=[]*)[source]¶
:   Bases: `torch.nn.modules.module.Module`

    `forward`(*x*)[source]¶
    :   Defines the computation performed at every call.

        Should be overridden by all subclasses.

        Note

        Although the recipe for forward pass needs to be defined within
        this function, one should call the `Module` instance afterwards
        instead of this since the former takes care of running the
        registered hooks while the latter silently ignores them.

    `forward_masks`(*x*)[source]¶

    `training` *= None*¶

*class* `pytorch_tabnet.tab_network.``TabNetDecoder`(*input\_dim*, *n\_d=8*, *n\_steps=3*, *n\_independent=1*, *n\_shared=1*, *virtual\_batch\_size=128*, *momentum=0.02*)[source]¶
:   Bases: `torch.nn.modules.module.Module`

    `forward`(*steps\_output*)[source]¶
    :   Defines the computation performed at every call.

        Should be overridden by all subclasses.

        Note

        Although the recipe for forward pass needs to be defined within
        this function, one should call the `Module` instance afterwards
        instead of this since the former takes care of running the
        registered hooks while the latter silently ignores them.

    `training` *= None*¶

*class* `pytorch_tabnet.tab_network.``TabNetEncoder`(*input\_dim*, *output\_dim*, *n\_d=8*, *n\_a=8*, *n\_steps=3*, *gamma=1.3*, *n\_independent=2*, *n\_shared=2*, *epsilon=1e-15*, *virtual\_batch\_size=128*, *momentum=0.02*, *mask\_type='sparsemax'*, *group\_attention\_matrix=None*)[source]¶
:   Bases: `torch.nn.modules.module.Module`

    `forward`(*x*, *prior=None*)[source]¶
    :   Defines the computation performed at every call.

        Should be overridden by all subclasses.

        Note

        Although the recipe for forward pass needs to be defined within
        this function, one should call the `Module` instance afterwards
        instead of this since the former takes care of running the
        registered hooks while the latter silently ignores them.

    `forward_masks`(*x*)[source]¶

    `training` *= None*¶

*class* `pytorch_tabnet.tab_network.``TabNetNoEmbeddings`(*input\_dim*, *output\_dim*, *n\_d=8*, *n\_a=8*, *n\_steps=3*, *gamma=1.3*, *n\_independent=2*, *n\_shared=2*, *epsilon=1e-15*, *virtual\_batch\_size=128*, *momentum=0.02*, *mask\_type='sparsemax'*, *group\_attention\_matrix=None*)[source]¶
:   Bases: `torch.nn.modules.module.Module`

    `forward`(*x*)[source]¶
    :   Defines the computation performed at every call.

        Should be overridden by all subclasses.

        Note

        Although the recipe for forward pass needs to be defined within
        this function, one should call the `Module` instance afterwards
        instead of this since the former takes care of running the
        registered hooks while the latter silently ignores them.

    `forward_masks`(*x*)[source]¶

    `training` *= None*¶

*class* `pytorch_tabnet.tab_network.``TabNetPretraining`(*input\_dim*, *pretraining\_ratio=0.2*, *n\_d=8*, *n\_a=8*, *n\_steps=3*, *gamma=1.3*, *cat\_idxs=[]*, *cat\_dims=[]*, *cat\_emb\_dim=1*, *n\_independent=2*, *n\_shared=2*, *epsilon=1e-15*, *virtual\_batch\_size=128*, *momentum=0.02*, *mask\_type='sparsemax'*, *n\_shared\_decoder=1*, *n\_indep\_decoder=1*, *group\_attention\_matrix=None*)[source]¶
:   Bases: `torch.nn.modules.module.Module`

    `forward`(*x*)[source]¶
    :   Returns: res, embedded\_x, obf\_vars
        :   res : output of reconstruction
            embedded\_x : embedded input
            obf\_vars : which variable where obfuscated

    `forward_masks`(*x*)[source]¶

    `training` *= None*¶

`pytorch_tabnet.tab_network.``initialize_glu`(*module*, *input\_dim*, *output\_dim*)[source]¶

`pytorch_tabnet.tab_network.``initialize_non_glu`(*module*, *input\_dim*, *output\_dim*)[source]¶

## pytorch\_tabnet.metrics module¶

*class* `pytorch_tabnet.metrics.``AUC`[source]¶
:   Bases: `pytorch_tabnet.metrics.Metric`

    AUC.

*class* `pytorch_tabnet.metrics.``Accuracy`[source]¶
:   Bases: `pytorch_tabnet.metrics.Metric`

    Accuracy.

*class* `pytorch_tabnet.metrics.``BalancedAccuracy`[source]¶
:   Bases: `pytorch_tabnet.metrics.Metric`

    Balanced Accuracy.

*class* `pytorch_tabnet.metrics.``LogLoss`[source]¶
:   Bases: `pytorch_tabnet.metrics.Metric`

    LogLoss.

*class* `pytorch_tabnet.metrics.``MAE`[source]¶
:   Bases: `pytorch_tabnet.metrics.Metric`

    Mean Absolute Error.

*class* `pytorch_tabnet.metrics.``MSE`[source]¶
:   Bases: `pytorch_tabnet.metrics.Metric`

    Mean Squared Error.

*class* `pytorch_tabnet.metrics.``Metric`[source]¶
:   Bases: `object`

    *classmethod* `get_metrics_by_names`(*names*)[source]¶
    :   Get list of metric classes.

        Parameters
        :   - **cls** (*Metric*) – Metric class.
            - **names** (*list*) – List of metric names.

        Returns
        :   **metrics** – List of metric classes.

        Return type
        :   list

*class* `pytorch_tabnet.metrics.``MetricContainer`(*metric\_names: List[str], prefix: str = ''*)[source]¶
:   Bases: `object`

    Container holding a list of metrics.

    Parameters
    :   - **metric\_names** (*list of str*) – List of metric names.
        - **prefix** (*str*) – Prefix of metric names.

    `metric_names`*: List[str]* *= None*¶

    `prefix`*: str* *= ''*¶

*class* `pytorch_tabnet.metrics.``RMSE`[source]¶
:   Bases: `pytorch_tabnet.metrics.Metric`

    Root Mean Squared Error.

*class* `pytorch_tabnet.metrics.``RMSLE`[source]¶
:   Bases: `pytorch_tabnet.metrics.Metric`

    Root Mean squared logarithmic error regression loss.
    Scikit-implementation:
    https://scikit-learn.org/stable/modules/generated/sklearn.metrics.mean\_squared\_log\_error.html
    Note: In order to avoid error, negative predictions are clipped to 0.
    This means that you should clip negative predictions manually after calling predict.

*class* `pytorch_tabnet.metrics.``UnsupMetricContainer`(*metric\_names: List[str], prefix: str = ''*)[source]¶
:   Bases: `object`

    Container holding a list of metrics.

    Parameters
    :   - **y\_pred** (*torch.Tensor* *or* *np.array*) – Reconstructed prediction (with embeddings)
        - **embedded\_x** (*torch.Tensor*) – Original input embedded by network
        - **obf\_vars** (*torch.Tensor*) – Binary mask for obfuscated variables.
          1 means the variables was obfuscated so reconstruction is based on this.

    `metric_names`*: List[str]* *= None*¶

    `prefix`*: str* *= ''*¶

`pytorch_tabnet.metrics.``UnsupervisedLoss`(*y\_pred*, *embedded\_x*, *obf\_vars*, *eps=1e-09*)[source]¶
:   Implements unsupervised loss function.
    This differs from orginal paper as it’s scaled to be batch size independent
    and number of features reconstructed independent (by taking the mean)

    Parameters
    :   - **y\_pred** (*torch.Tensor* *or* *np.array*) – Reconstructed prediction (with embeddings)
        - **embedded\_x** (*torch.Tensor*) – Original input embedded by network
        - **obf\_vars** (*torch.Tensor*) – Binary mask for obfuscated variables.
          1 means the variable was obfuscated so reconstruction is based on this.
        - **eps** (*float*) – A small floating point to avoid ZeroDivisionError
          This can happen in degenerated case when a feature has only one value

    Returns
    :   **loss** – Unsupervised loss, average value over batch samples.

    Return type
    :   torch float

`pytorch_tabnet.metrics.``UnsupervisedLossNumpy`(*y\_pred*, *embedded\_x*, *obf\_vars*, *eps=1e-09*)[source]¶

*class* `pytorch_tabnet.metrics.``UnsupervisedMetric`[source]¶
:   Bases: `pytorch_tabnet.metrics.Metric`

    Unsupervised metric

*class* `pytorch_tabnet.metrics.``UnsupervisedNumpyMetric`[source]¶
:   Bases: `pytorch_tabnet.metrics.Metric`

    Unsupervised metric

`pytorch_tabnet.metrics.``check_metrics`(*metrics*)[source]¶
:   Check if custom metrics are provided.

    Parameters
    :   **metrics** (*list of str* *or* *classes*) – List with built-in metrics (str) or custom metrics (classes).

    Returns
    :   **val\_metrics** – List of metric names.

    Return type
    :   list of str

## pytorch\_tabnet.tab\_model module¶

*class* `pytorch_tabnet.tab_model.``TabNetClassifier`(*n\_d: int = 8*, *n\_a: int = 8*, *n\_steps: int = 3*, *gamma: float = 1.3*, *cat\_idxs: List[int] = <factory>*, *cat\_dims: List[int] = <factory>*, *cat\_emb\_dim: int = 1*, *n\_independent: int = 2*, *n\_shared: int = 2*, *epsilon: float = 1e-15*, *momentum: float = 0.02*, *lambda\_sparse: float = 0.001*, *seed: int = 0*, *clip\_value: int = 1*, *verbose: int = 1*, *optimizer\_fn: Any = <class 'torch.optim.adam.Adam'>*, *optimizer\_params: Dict = <factory>*, *scheduler\_fn: Any = None*, *scheduler\_params: Dict = <factory>*, *mask\_type: str = 'sparsemax'*, *input\_dim: int = None*, *output\_dim: int = None*, *device\_name: str = 'auto'*, *n\_shared\_decoder: int = 1*, *n\_indep\_decoder: int = 1*, *grouped\_features: List[List[int]] = <factory>*)[source]¶
:   Bases: `pytorch_tabnet.abstract_model.TabModel`

    `cat_dims` *= None*¶

    `cat_idxs` *= None*¶

    `compute_loss`(*y\_pred*, *y\_true*)[source]¶
    :   Compute the loss.

        Parameters
        :   - **y\_score** (a :tensor: torch.Tensor) – Score matrix
            - **y\_true** (a :tensor: torch.Tensor) – Target matrix

        Returns
        :   Loss value

        Return type
        :   float

    `grouped_features` *= None*¶

    `optimizer_params` *= None*¶

    `predict_func`(*outputs*)[source]¶

    `predict_proba`(*X*)[source]¶
    :   Make predictions for classification on a batch (valid)

        Parameters
        :   **X** (a :tensor: torch.Tensor or matrix: scipy.sparse.csr\_matrix) – Input data

        Returns
        :   **res**

        Return type
        :   np.ndarray

    `prepare_target`(*y*)[source]¶
    :   Prepare target before training.

        Parameters
        :   **y** (a :tensor: torch.Tensor) – Target matrix.

        Returns
        :   Converted target matrix.

        Return type
        :   torch.Tensor

    `scheduler_params` *= None*¶

    `stack_batches`(*list\_y\_true*, *list\_y\_score*)[source]¶

    `update_fit_params`(*X\_train*, *y\_train*, *eval\_set*, *weights*)[source]¶
    :   Set attributes relative to fit function.

        Parameters
        :   - **X\_train** (*np.ndarray*) – Train set
            - **y\_train** (*np.array*) – Train targets
            - **eval\_set** (*list of tuple*) – List of eval tuple set (X, y).
            - **weights** (*bool* *or* *dictionnary*) – 0 for no balancing
              1 for automated balancing

    `weight_updater`(*weights*)[source]¶
    :   Updates weights dictionary according to target\_mapper.

        Parameters
        :   **weights** (*bool* *or* *dict*) – Given weights for balancing training.

        Returns
        :   Same bool if weights are bool, updated dict otherwise.

        Return type
        :   bool or dict

*class* `pytorch_tabnet.tab_model.``TabNetRegressor`(*n\_d: int = 8*, *n\_a: int = 8*, *n\_steps: int = 3*, *gamma: float = 1.3*, *cat\_idxs: List[int] = <factory>*, *cat\_dims: List[int] = <factory>*, *cat\_emb\_dim: int = 1*, *n\_independent: int = 2*, *n\_shared: int = 2*, *epsilon: float = 1e-15*, *momentum: float = 0.02*, *lambda\_sparse: float = 0.001*, *seed: int = 0*, *clip\_value: int = 1*, *verbose: int = 1*, *optimizer\_fn: Any = <class 'torch.optim.adam.Adam'>*, *optimizer\_params: Dict = <factory>*, *scheduler\_fn: Any = None*, *scheduler\_params: Dict = <factory>*, *mask\_type: str = 'sparsemax'*, *input\_dim: int = None*, *output\_dim: int = None*, *device\_name: str = 'auto'*, *n\_shared\_decoder: int = 1*, *n\_indep\_decoder: int = 1*, *grouped\_features: List[List[int]] = <factory>*)[source]¶
:   Bases: `pytorch_tabnet.abstract_model.TabModel`

    `cat_dims` *= None*¶

    `cat_idxs` *= None*¶

    `compute_loss`(*y\_pred*, *y\_true*)[source]¶
    :   Compute the loss.

        Parameters
        :   - **y\_score** (a :tensor: torch.Tensor) – Score matrix
            - **y\_true** (a :tensor: torch.Tensor) – Target matrix

        Returns
        :   Loss value

        Return type
        :   float

    `grouped_features` *= None*¶

    `optimizer_params` *= None*¶

    `predict_func`(*outputs*)[source]¶

    `prepare_target`(*y*)[source]¶
    :   Prepare target before training.

        Parameters
        :   **y** (a :tensor: torch.Tensor) – Target matrix.

        Returns
        :   Converted target matrix.

        Return type
        :   torch.Tensor

    `scheduler_params` *= None*¶

    `stack_batches`(*list\_y\_true*, *list\_y\_score*)[source]¶

    `update_fit_params`(*X\_train*, *y\_train*, *eval\_set*, *weights*)[source]¶
    :   Set attributes relative to fit function.

        Parameters
        :   - **X\_train** (*np.ndarray*) – Train set
            - **y\_train** (*np.array*) – Train targets
            - **eval\_set** (*list of tuple*) – List of eval tuple set (X, y).
            - **weights** (*bool* *or* *dictionnary*) – 0 for no balancing
              1 for automated balancing

## pytorch\_tabnet.sparsemax module¶

*class* `pytorch_tabnet.sparsemax.``Entmax15`(*dim=-1*)[source]¶
:   Bases: `torch.nn.modules.module.Module`

    `forward`(*input*)[source]¶
    :   Defines the computation performed at every call.

        Should be overridden by all subclasses.

        Note

        Although the recipe for forward pass needs to be defined within
        this function, one should call the `Module` instance afterwards
        instead of this since the former takes care of running the
        registered hooks while the latter silently ignores them.

    `training` *= None*¶

*class* `pytorch_tabnet.sparsemax.``Entmax15Function`(*\*args*, *\*\*kwargs*)[source]¶
:   Bases: `torch.autograd.function.Function`

    An implementation of exact Entmax with alpha=1.5 (B. Peters, V. Niculae, A. Martins). See
    :cite:`https://arxiv.org/abs/1905.05702 for detailed description.
    Source: https://github.com/deep-spin/entmax

    *static* `backward`(*ctx*, *grad\_output*)[source]¶
    :   Defines a formula for differentiating the operation with backward mode
        automatic differentiation (alias to the vjp function).

        This function is to be overridden by all subclasses.

        It must accept a context `ctx` as the first argument, followed by
        as many outputs as the `forward()` returned (None will be passed in
        for non tensor outputs of the forward function),
        and it should return as many tensors, as there were inputs to
        `forward()`. Each argument is the gradient w.r.t the given output,
        and each returned value should be the gradient w.r.t. the
        corresponding input. If an input is not a Tensor or is a Tensor not
        requiring grads, you can just pass None as a gradient for that input.

        The context can be used to retrieve tensors saved during the forward
        pass. It also has an attribute `ctx.needs_input_grad` as a tuple
        of booleans representing whether each input needs gradient. E.g.,
        `backward()` will have `ctx.needs_input_grad[0] = True` if the
        first input to `forward()` needs gradient computated w.r.t. the
        output.

    *static* `forward`(*ctx*, *input*, *dim=-1*)[source]¶
    :   Performs the operation.

        This function is to be overridden by all subclasses.

        It must accept a context ctx as the first argument, followed by any
        number of arguments (tensors or other types).

        The context can be used to store arbitrary data that can be then
        retrieved during the backward pass. Tensors should not be stored
        directly on ctx (though this is not currently enforced for
        backward compatibility). Instead, tensors should be saved either with
        `ctx.save_for_backward()` if they are intended to be used in
        `backward` (equivalently, `vjp`) or `ctx.save_for_forward()`
        if they are intended to be used for in `jvp`.

*class* `pytorch_tabnet.sparsemax.``Entmoid15`(*\*args*, *\*\*kwargs*)[source]¶
:   Bases: `torch.autograd.function.Function`

    A highly optimized equivalent of lambda x: Entmax15([x, 0])

    *static* `backward`(*ctx*, *grad\_output*)[source]¶
    :   Defines a formula for differentiating the operation with backward mode
        automatic differentiation (alias to the vjp function).

        This function is to be overridden by all subclasses.

        It must accept a context `ctx` as the first argument, followed by
        as many outputs as the `forward()` returned (None will be passed in
        for non tensor outputs of the forward function),
        and it should return as many tensors, as there were inputs to
        `forward()`. Each argument is the gradient w.r.t the given output,
        and each returned value should be the gradient w.r.t. the
        corresponding input. If an input is not a Tensor or is a Tensor not
        requiring grads, you can just pass None as a gradient for that input.

        The context can be used to retrieve tensors saved during the forward
        pass. It also has an attribute `ctx.needs_input_grad` as a tuple
        of booleans representing whether each input needs gradient. E.g.,
        `backward()` will have `ctx.needs_input_grad[0] = True` if the
        first input to `forward()` needs gradient computated w.r.t. the
        output.

    *static* `forward`(*ctx*, *input*)[source]¶
    :   Performs the operation.

        This function is to be overridden by all subclasses.

        It must accept a context ctx as the first argument, followed by any
        number of arguments (tensors or other types).

        The context can be used to store arbitrary data that can be then
        retrieved during the backward pass. Tensors should not be stored
        directly on ctx (though this is not currently enforced for
        backward compatibility). Instead, tensors should be saved either with
        `ctx.save_for_backward()` if they are intended to be used in
        `backward` (equivalently, `vjp`) or `ctx.save_for_forward()`
        if they are intended to be used for in `jvp`.

*class* `pytorch_tabnet.sparsemax.``Sparsemax`(*dim=-1*)[source]¶
:   Bases: `torch.nn.modules.module.Module`

    `forward`(*input*)[source]¶
    :   Defines the computation performed at every call.

        Should be overridden by all subclasses.

        Note

        Although the recipe for forward pass needs to be defined within
        this function, one should call the `Module` instance afterwards
        instead of this since the former takes care of running the
        registered hooks while the latter silently ignores them.

    `training` *= None*¶

*class* `pytorch_tabnet.sparsemax.``SparsemaxFunction`(*\*args*, *\*\*kwargs*)[source]¶
:   Bases: `torch.autograd.function.Function`

    An implementation of sparsemax (Martins & Astudillo, 2016). See
    :cite:`DBLP:journals/corr/MartinsA16` for detailed description.
    By Ben Peters and Vlad Niculae

    *static* `backward`(*ctx*, *grad\_output*)[source]¶
    :   Defines a formula for differentiating the operation with backward mode
        automatic differentiation (alias to the vjp function).

        This function is to be overridden by all subclasses.

        It must accept a context `ctx` as the first argument, followed by
        as many outputs as the `forward()` returned (None will be passed in
        for non tensor outputs of the forward function),
        and it should return as many tensors, as there were inputs to
        `forward()`. Each argument is the gradient w.r.t the given output,
        and each returned value should be the gradient w.r.t. the
        corresponding input. If an input is not a Tensor or is a Tensor not
        requiring grads, you can just pass None as a gradient for that input.

        The context can be used to retrieve tensors saved during the forward
        pass. It also has an attribute `ctx.needs_input_grad` as a tuple
        of booleans representing whether each input needs gradient. E.g.,
        `backward()` will have `ctx.needs_input_grad[0] = True` if the
        first input to `forward()` needs gradient computated w.r.t. the
        output.

    *static* `forward`(*ctx*, *input*, *dim=-1*)[source]¶
    :   sparsemax: normalizing sparse transform (a la softmax)

        Parameters
        :   - **ctx** (*torch.autograd.function.\_ContextMethodMixin*) –
            - **input** (*torch.Tensor*) – any shape
            - **dim** (*int*) – dimension along which to apply sparsemax

        Returns
        :   **output** – same shape as input

        Return type
        :   torch.Tensor

`pytorch_tabnet.sparsemax.``entmax15`()¶

`pytorch_tabnet.sparsemax.``entmoid15`()¶

`pytorch_tabnet.sparsemax.``sparsemax`()¶

## pytorch\_tabnet.callbacks module¶

*class* `pytorch_tabnet.callbacks.``Callback`[source]¶
:   Bases: `object`

    Abstract base class used to build new callbacks.

    `on_batch_begin`(*batch*, *logs=None*)[source]¶

    `on_batch_end`(*batch*, *logs=None*)[source]¶

    `on_epoch_begin`(*epoch*, *logs=None*)[source]¶

    `on_epoch_end`(*epoch*, *logs=None*)[source]¶

    `on_train_begin`(*logs=None*)[source]¶

    `on_train_end`(*logs=None*)[source]¶

    `set_params`(*params*)[source]¶

    `set_trainer`(*model*)[source]¶

*class* `pytorch_tabnet.callbacks.``CallbackContainer`(*callbacks: List[pytorch\_tabnet.callbacks.Callback] = <factory>*)[source]¶
:   Bases: `object`

    Container holding a list of callbacks.

    `append`(*callback*)[source]¶

    `callbacks`*: List[Callback]* *= None*¶

    `on_batch_begin`(*batch*, *logs=None*)[source]¶

    `on_batch_end`(*batch*, *logs=None*)[source]¶

    `on_epoch_begin`(*epoch*, *logs=None*)[source]¶

    `on_epoch_end`(*epoch*, *logs=None*)[source]¶

    `on_train_begin`(*logs=None*)[source]¶

    `on_train_end`(*logs=None*)[source]¶

    `set_params`(*params*)[source]¶

    `set_trainer`(*trainer*)[source]¶

*class* `pytorch_tabnet.callbacks.``EarlyStopping`(*early\_stopping\_metric: str*, *is\_maximize: bool*, *tol: float = 0.0*, *patience: int = 5*)[source]¶
:   Bases: `pytorch_tabnet.callbacks.Callback`

    EarlyStopping callback to exit the training loop if early\_stopping\_metric
    does not improve by a certain amount for a certain
    number of epochs.

    Parameters
    :   - **early\_stopping\_metric** (*str*) – Early stopping metric name
        - **is\_maximize** (*bool*) – Whether to maximize or not early\_stopping\_metric
        - **tol** (*float*) – minimum change in monitored value to qualify as improvement.
          This number should be positive.
        - **patience** (*integer*) – number of epochs to wait for improvement before terminating.
          the counter be reset after each improvement

    `early_stopping_metric`*: str* *= None*¶

    `is_maximize`*: bool* *= None*¶

    `on_epoch_end`(*epoch*, *logs=None*)[source]¶

    `on_train_end`(*logs=None*)[source]¶

    `patience`*: int* *= 5*¶

    `tol`*: float* *= 0.0*¶

*class* `pytorch_tabnet.callbacks.``History`(*trainer: Any*, *verbose: int = 1*)[source]¶
:   Bases: `pytorch_tabnet.callbacks.Callback`

    Callback that records events into a History object.
    This callback is automatically applied to
    every SuperModule.

    Parameters
    :   - **trainer** (*DeepRecoModel*) – Model class to train
        - **verbose** (*int*) – Print results every verbose iteration

    `on_batch_end`(*batch*, *logs=None*)[source]¶

    `on_epoch_begin`(*epoch*, *logs=None*)[source]¶

    `on_epoch_end`(*epoch*, *logs=None*)[source]¶

    `on_train_begin`(*logs=None*)[source]¶

    `trainer`*: Any* *= None*¶

    `verbose`*: int* *= 1*¶

*class* `pytorch_tabnet.callbacks.``LRSchedulerCallback`(*scheduler\_fn: Any*, *optimizer: Any*, *scheduler\_params: dict*, *early\_stopping\_metric: str*, *is\_batch\_level: bool = False*)[source]¶
:   Bases: `pytorch_tabnet.callbacks.Callback`

    Wrapper for most torch scheduler functions.

    Parameters
    :   - **scheduler\_fn** (*torch.optim.lr\_scheduler*) – Torch scheduling class
        - **scheduler\_params** (*dict*) – Dictionnary containing all parameters for the scheduler\_fn
        - **is\_batch\_level** (*bool* *(**default = False**)*) – If set to False : lr updates will happen at every epoch
          If set to True : lr updates happen at every batch
          Set this to True for OneCycleLR for example

    `early_stopping_metric`*: str* *= None*¶

    `is_batch_level`*: bool* *= False*¶

    `on_batch_end`(*batch*, *logs=None*)[source]¶

    `on_epoch_end`(*epoch*, *logs=None*)[source]¶

    `optimizer`*: Any* *= None*¶

    `scheduler_fn`*: Any* *= None*¶

    `scheduler_params`*: dict* *= None*¶

## pytorch\_tabnet.abstract\_model module¶

*class* `pytorch_tabnet.abstract_model.``TabModel`(*n\_d: int = 8*, *n\_a: int = 8*, *n\_steps: int = 3*, *gamma: float = 1.3*, *cat\_idxs: List[int] = <factory>*, *cat\_dims: List[int] = <factory>*, *cat\_emb\_dim: int = 1*, *n\_independent: int = 2*, *n\_shared: int = 2*, *epsilon: float = 1e-15*, *momentum: float = 0.02*, *lambda\_sparse: float = 0.001*, *seed: int = 0*, *clip\_value: int = 1*, *verbose: int = 1*, *optimizer\_fn: Any = <class 'torch.optim.adam.Adam'>*, *optimizer\_params: Dict = <factory>*, *scheduler\_fn: Any = None*, *scheduler\_params: Dict = <factory>*, *mask\_type: str = 'sparsemax'*, *input\_dim: int = None*, *output\_dim: int = None*, *device\_name: str = 'auto'*, *n\_shared\_decoder: int = 1*, *n\_indep\_decoder: int = 1*, *grouped\_features: List[List[int]] = <factory>*)[source]¶
:   Bases: `sklearn.base.BaseEstimator`

    Class for TabNet model.

    `cat_dims`*: List[int]* *= None*¶

    `cat_emb_dim`*: int* *= 1*¶

    `cat_idxs`*: List[int]* *= None*¶

    `clip_value`*: int* *= 1*¶

    *abstract* `compute_loss`(*y\_score*, *y\_true*)[source]¶
    :   Compute the loss.

        Parameters
        :   - **y\_score** (a :tensor: torch.Tensor) – Score matrix
            - **y\_true** (a :tensor: torch.Tensor) – Target matrix

        Returns
        :   Loss value

        Return type
        :   float

    `device_name`*: str* *= 'auto'*¶

    `epsilon`*: float* *= 1e-15*¶

    `explain`(*X*, *normalize=False*)[source]¶
    :   Return local explanation

        Parameters
        :   - **X** (tensor: torch.Tensor or matrix: scipy.sparse.csr\_matrix) – Input data
            - **normalize** (*bool* *(**default False**)*) – Wheter to normalize so that sum of features are equal to 1

        Returns
        :   - **M\_explain** (*matrix*) – Importance per sample, per columns.
            - **masks** (*matrix*) – Sparse matrix showing attention masks used by network.

    `fit`(*X\_train*, *y\_train*, *eval\_set=None*, *eval\_name=None*, *eval\_metric=None*, *loss\_fn=None*, *weights=0*, *max\_epochs=100*, *patience=10*, *batch\_size=1024*, *virtual\_batch\_size=128*, *num\_workers=0*, *drop\_last=True*, *callbacks=None*, *pin\_memory=True*, *from\_unsupervised=None*, *warm\_start=False*, *augmentations=None*, *compute\_importance=True*)[source]¶
    :   Train a neural network stored in self.network
        Using train\_dataloader for training data and
        valid\_dataloader for validation.

        Parameters
        :   - **X\_train** (*np.ndarray*) – Train set
            - **y\_train** (*np.array*) – Train targets
            - **eval\_set** (*list of tuple*) – List of eval tuple set (X, y).
              The last one is used for early stopping
            - **eval\_name** (*list of str*) – List of eval set names.
            - **eval\_metric** (*list of str*) – List of evaluation metrics.
              The last metric is used for early stopping.
            - **loss\_fn** (*callable* *or* *None*) – a PyTorch loss function
            - **weights** (*bool* *or* *dictionnary*) – 0 for no balancing
              1 for automated balancing
              dict for custom weights per class
            - **max\_epochs** (*int*) – Maximum number of epochs during training
            - **patience** (*int*) – Number of consecutive non improving epoch before early stopping
            - **batch\_size** (*int*) – Training batch size
            - **virtual\_batch\_size** (*int*) – Batch size for Ghost Batch Normalization (virtual\_batch\_size < batch\_size)
            - **num\_workers** (*int*) – Number of workers used in torch.utils.data.DataLoader
            - **drop\_last** (*bool*) – Whether to drop last batch during training
            - **callbacks** (*list of callback function*) – List of custom callbacks
            - **pin\_memory** (*bool*) – Whether to set pin\_memory to True or False during training
            - **from\_unsupervised** (*unsupervised trained model*) – Use a previously self supervised model as starting weights
            - **warm\_start** (*bool*) – If True, current model parameters are used to start training
            - **compute\_importance** (*bool*) – Whether to compute feature importance

    `gamma`*: float* *= 1.3*¶

    `grouped_features`*: List[List[int]]* *= None*¶

    `input_dim`*: int* *= None*¶

    `lambda_sparse`*: float* *= 0.001*¶

    `load_class_attrs`(*class\_attrs*)[source]¶

    `load_model`(*filepath*)[source]¶
    :   Load TabNet model.

        Parameters
        :   **filepath** (*str*) – Path of the model.

    `load_weights_from_unsupervised`(*unsupervised\_model*)[source]¶

    `mask_type`*: str* *= 'sparsemax'*¶

    `momentum`*: float* *= 0.02*¶

    `n_a`*: int* *= 8*¶

    `n_d`*: int* *= 8*¶

    `n_indep_decoder`*: int* *= 1*¶

    `n_independent`*: int* *= 2*¶

    `n_shared`*: int* *= 2*¶

    `n_shared_decoder`*: int* *= 1*¶

    `n_steps`*: int* *= 3*¶

    `optimizer_fn`¶
    :   alias of `torch.optim.adam.Adam`

    `optimizer_params`*: Dict* *= None*¶

    `output_dim`*: int* *= None*¶

    `predict`(*X*)[source]¶
    :   Make predictions on a batch (valid)

        Parameters
        :   **X** (a :tensor: torch.Tensor or matrix: scipy.sparse.csr\_matrix) – Input data

        Returns
        :   **predictions** – Predictions of the regression problem

        Return type
        :   np.array

    *abstract* `prepare_target`(*y*)[source]¶
    :   Prepare target before training.

        Parameters
        :   **y** (a :tensor: torch.Tensor) – Target matrix.

        Returns
        :   Converted target matrix.

        Return type
        :   torch.Tensor

    `save_model`(*path*)[source]¶
    :   Saving TabNet model in two distinct files.

        Parameters
        :   **path** (*str*) – Path of the model.

        Returns
        :   input filepath with “.zip” appended

        Return type
        :   str

    `scheduler_fn`*: Any* *= None*¶

    `scheduler_params`*: Dict* *= None*¶

    `seed`*: int* *= 0*¶

    *abstract* `update_fit_params`(*X\_train*, *y\_train*, *eval\_set*, *weights*)[source]¶
    :   Set attributes relative to fit function.

        Parameters
        :   - **X\_train** (*np.ndarray*) – Train set
            - **y\_train** (*np.array*) – Train targets
            - **eval\_set** (*list of tuple*) – List of eval tuple set (X, y).
            - **weights** (*bool* *or* *dictionnary*) – 0 for no balancing
              1 for automated balancing

    `verbose`*: int* *= 1*¶

## pytorch\_tabnet.pretraining module¶

*class* `pytorch_tabnet.pretraining.``TabNetPretrainer`(*n\_d: int = 8*, *n\_a: int = 8*, *n\_steps: int = 3*, *gamma: float = 1.3*, *cat\_idxs: List[int] = <factory>*, *cat\_dims: List[int] = <factory>*, *cat\_emb\_dim: int = 1*, *n\_independent: int = 2*, *n\_shared: int = 2*, *epsilon: float = 1e-15*, *momentum: float = 0.02*, *lambda\_sparse: float = 0.001*, *seed: int = 0*, *clip\_value: int = 1*, *verbose: int = 1*, *optimizer\_fn: Any = <class 'torch.optim.adam.Adam'>*, *optimizer\_params: Dict = <factory>*, *scheduler\_fn: Any = None*, *scheduler\_params: Dict = <factory>*, *mask\_type: str = 'sparsemax'*, *input\_dim: int = None*, *output\_dim: int = None*, *device\_name: str = 'auto'*, *n\_shared\_decoder: int = 1*, *n\_indep\_decoder: int = 1*, *grouped\_features: List[List[int]] = <factory>*)[source]¶
:   Bases: `pytorch_tabnet.abstract_model.TabModel`

    `cat_dims` *= None*¶

    `cat_idxs` *= None*¶

    `compute_loss`(*output*, *embedded\_x*, *obf\_vars*)[source]¶
    :   Compute the loss.

        Parameters
        :   - **y\_score** (a :tensor: torch.Tensor) – Score matrix
            - **y\_true** (a :tensor: torch.Tensor) – Target matrix

        Returns
        :   Loss value

        Return type
        :   float

    `fit`(*X\_train*, *eval\_set=None*, *eval\_name=None*, *loss\_fn=None*, *pretraining\_ratio=0.5*, *weights=0*, *max\_epochs=100*, *patience=10*, *batch\_size=1024*, *virtual\_batch\_size=128*, *num\_workers=0*, *drop\_last=True*, *callbacks=None*, *pin\_memory=True*, *warm\_start=False*)[source]¶
    :   Train a neural network stored in self.network
        Using train\_dataloader for training data and
        valid\_dataloader for validation.

        Parameters
        :   - **X\_train** (*np.ndarray*) – Train set to reconstruct in self supervision
            - **eval\_set** (*list of np.array*) – List of evaluation set
              The last one is used for early stopping
            - **eval\_name** (*list of str*) – List of eval set names.
            - **eval\_metric** (*list of str*) – List of evaluation metrics.
              The last metric is used for early stopping.
            - **loss\_fn** (*callable* *or* *None*) – a PyTorch loss function
              should be left to None for self supervised and non experts
            - **pretraining\_ratio** (*float*) – Between 0 and 1, percentage of feature to mask for reconstruction
            - **weights** (*np.array*) – Sampling weights for each example.
            - **max\_epochs** (*int*) – Maximum number of epochs during training
            - **patience** (*int*) – Number of consecutive non improving epoch before early stopping
            - **batch\_size** (*int*) – Training batch size
            - **virtual\_batch\_size** (*int*) – Batch size for Ghost Batch Normalization (virtual\_batch\_size < batch\_size)
            - **num\_workers** (*int*) – Number of workers used in torch.utils.data.DataLoader
            - **drop\_last** (*bool*) – Whether to drop last batch during training
            - **callbacks** (*list of callback function*) – List of custom callbacks
            - **pin\_memory** (*bool*) – Whether to set pin\_memory to True or False during training

    `grouped_features` *= None*¶

    `optimizer_params` *= None*¶

    `predict`(*X*)[source]¶
    :   Make predictions on a batch (valid)

        Parameters
        :   **X** (a :tensor: torch.Tensor or matrix: scipy.sparse.csr\_matrix) – Input data

        Returns
        :   **predictions** – Predictions of the regression problem

        Return type
        :   np.array

    `prepare_target`(*y*)[source]¶
    :   Prepare target before training.

        Parameters
        :   **y** (a :tensor: torch.Tensor) – Target matrix.

        Returns
        :   Converted target matrix.

        Return type
        :   torch.Tensor

    `scheduler_params` *= None*¶

    `stack_batches`(*list\_output*, *list\_embedded\_x*, *list\_obfuscation*)[source]¶

    `update_fit_params`(*weights*)[source]¶
    :   Set attributes relative to fit function.

        Parameters
        :   - **X\_train** (*np.ndarray*) – Train set
            - **y\_train** (*np.array*) – Train targets
            - **eval\_set** (*list of tuple*) – List of eval tuple set (X, y).
            - **weights** (*bool* *or* *dictionnary*) – 0 for no balancing
              1 for automated balancing

## pytorch\_tabnet.utils module¶

*class* `pytorch_tabnet.utils.``ComplexEncoder`(*\**, *skipkeys=False*, *ensure\_ascii=True*, *check\_circular=True*, *allow\_nan=True*, *sort\_keys=False*, *indent=None*, *separators=None*, *default=None*)[source]¶
:   Bases: `json.encoder.JSONEncoder`

    `default`(*obj*)[source]¶
    :   Implement this method in a subclass such that it returns
        a serializable object for `o`, or calls the base implementation
        (to raise a `TypeError`).

        For example, to support arbitrary iterators, you could
        implement default like this:

        ```
        def default(self, o):
            try:
                iterable = iter(o)
            except TypeError:
                pass
            else:
                return list(iterable)
            # Let the base class default method raise the TypeError
            return JSONEncoder.default(self, o)
        ```

*class* `pytorch_tabnet.utils.``PredictDataset`(*x*)[source]¶
:   Bases: `torch.utils.data.dataset.Dataset`

    Format for numpy array

    Parameters
    :   **X** (*2D array*) – The input matrix

*class* `pytorch_tabnet.utils.``SparsePredictDataset`(*x*)[source]¶
:   Bases: `torch.utils.data.dataset.Dataset`

    Format for csr\_matrix

    Parameters
    :   **X** (*CSR matrix*) – The input matrix

*class* `pytorch_tabnet.utils.``SparseTorchDataset`(*x*, *y*)[source]¶
:   Bases: `torch.utils.data.dataset.Dataset`

    Format for csr\_matrix

    Parameters
    :   - **X** (*CSR matrix*) – The input matrix
        - **y** (*2D array*) – The one-hot encoded target

*class* `pytorch_tabnet.utils.``TorchDataset`(*x*, *y*)[source]¶
:   Bases: `torch.utils.data.dataset.Dataset`

    Format for numpy array

    Parameters
    :   - **X** (*2D array*) – The input matrix
        - **y** (*2D array*) – The one-hot encoded target

`pytorch_tabnet.utils.``check_embedding_parameters`(*cat\_dims*, *cat\_idxs*, *cat\_emb\_dim*)[source]¶
:   Check parameters related to embeddings and rearrange them in a unique manner.

`pytorch_tabnet.utils.``check_input`(*X*)[source]¶
:   Raise a clear error if X is a pandas dataframe
    and check array according to scikit rules

`pytorch_tabnet.utils.``check_list_groups`(*list\_groups*, *input\_dim*)[source]¶
:   Check that list groups:
    :   - is a list of list
        - does not contain twice the same feature in different groups
        - does not contain unknown features (>= input\_dim)
        - does not contain empty groups

    Parameters
    :   - **list\_groups** (*-*) – Each element is a list representing features in the same group.
          One feature should appear in maximum one group.
          Feature that don’t get assign a group will be in their own group of one feature.
        - **input\_dim** (*-*) –

`pytorch_tabnet.utils.``check_warm_start`(*warm\_start*, *from\_unsupervised*)[source]¶
:   Gives a warning about ambiguous usage of the two parameters.

`pytorch_tabnet.utils.``create_dataloaders`(*X\_train*, *y\_train*, *eval\_set*, *weights*, *batch\_size*, *num\_workers*, *drop\_last*, *pin\_memory*)[source]¶
:   Create dataloaders with or without subsampling depending on weights and balanced.

    Parameters
    :   - **X\_train** (*np.ndarray*) – Training data
        - **y\_train** (*np.array*) – Mapped Training targets
        - **eval\_set** (*list of tuple*) – List of eval tuple set (X, y)
        - **weights** (*either 0**,* *1**,* *dict* *or* *iterable*) –

          if 0 (default) : no weights will be applied
          if 1 : classification only, will balanced class with inverse frequency
          if dict : keys are corresponding class values are sample weights
          if iterable : list or np array must be of length equal to nb elements

          > in the training set
        - **batch\_size** (*int*) – how many samples per batch to load
        - **num\_workers** (*int*) – how many subprocesses to use for data loading. 0 means that the data
          will be loaded in the main process
        - **drop\_last** (*bool*) – set to True to drop the last incomplete batch, if the dataset size is not
          divisible by the batch size. If False and the size of dataset is not
          divisible by the batch size, then the last batch will be smaller
        - **pin\_memory** (*bool*) – Whether to pin GPU memory during training

    Returns
    :   **train\_dataloader, valid\_dataloader** – Training and validation dataloaders

    Return type
    :   torch.DataLoader, torch.DataLoader

`pytorch_tabnet.utils.``create_explain_matrix`(*input\_dim*, *cat\_emb\_dim*, *cat\_idxs*, *post\_embed\_dim*)[source]¶
:   This is a computational trick.
    In order to rapidly sum importances from same embeddings
    to the initial index.

    Parameters
    :   - **input\_dim** (*int*) – Initial input dim
        - **cat\_emb\_dim** (*int* *or* *list of int*) – if int : size of embedding for all categorical feature
          if list of int : size of embedding for each categorical feature
        - **cat\_idxs** (*list of int*) – Initial position of categorical features
        - **post\_embed\_dim** (*int*) – Post embedding inputs dimension

    Returns
    :   **reducing\_matrix** – Matrix of dim (post\_embed\_dim, input\_dim) to performe reduce

    Return type
    :   np.array

`pytorch_tabnet.utils.``create_group_matrix`(*list\_groups*, *input\_dim*)[source]¶
:   Create the group matrix corresponding to the given list\_groups

    Parameters
    :   - **list\_groups** (*-*) – Each element is a list representing features in the same group.
          One feature should appear in maximum one group.
          Feature that don’t get assigned a group will be in their own group of one feature.
        - **input\_dim** (*-*) –

    Returns
    :   **- group\_matrix** – A matrix of size (n\_groups, input\_dim)
        where m\_ij represents the importance of feature j in group i
        The rows must some to 1 as each group is equally important a priori.

    Return type
    :   torch matrix

`pytorch_tabnet.utils.``create_sampler`(*weights*, *y\_train*)[source]¶
:   This creates a sampler from the given weights

    Parameters
    :   - **weights** (*either 0**,* *1**,* *dict* *or* *iterable*) –

          if 0 (default) : no weights will be applied
          if 1 : classification only, will balanced class with inverse frequency
          if dict : keys are corresponding class values are sample weights
          if iterable : list or np array must be of length equal to nb elements

          > in the training set
        - **y\_train** (*np.array*) – Training targets

`pytorch_tabnet.utils.``define_device`(*device\_name*)[source]¶
:   Define the device to use during training and inference.
    If auto it will detect automatically whether to use cuda or cpu

    Parameters
    :   **device\_name** (*str*) – Either “auto”, “cpu” or “cuda”

    Returns
    :   Either “cpu” or “cuda”

    Return type
    :   str

`pytorch_tabnet.utils.``filter_weights`(*weights*)[source]¶
:   This function makes sure that weights are in correct format for
    regression and multitask TabNet

    Parameters
    :   **weights** (*int**,* *dict* *or* *list*) – Initial weights parameters given by user

    Returns
    :   **None**

    Return type
    :   This function will only throw an error if format is wrong

`pytorch_tabnet.utils.``validate_eval_set`(*eval\_set*, *eval\_name*, *X\_train*, *y\_train*)[source]¶
:   Check if the shapes of eval\_set are compatible with (X\_train, y\_train).

    Parameters
    :   - **eval\_set** (*list of tuple*) – List of eval tuple set (X, y).
          The last one is used for early stopping
        - **eval\_name** (*list of str*) – List of eval set names.
        - **X\_train** (*np.ndarray*) – Train owned products
        - **y\_train** (*np.array*) – Train targeted products

    Returns
    :   - **eval\_names** (*list of str*) – Validated list of eval\_names.
        - **eval\_set** (*list of tuple*) – Validated list of eval\_set.

## pytorch\_tabnet.multitask module¶

*class* `pytorch_tabnet.multitask.``TabNetMultiTaskClassifier`(*n\_d: int = 8*, *n\_a: int = 8*, *n\_steps: int = 3*, *gamma: float = 1.3*, *cat\_idxs: List[int] = <factory>*, *cat\_dims: List[int] = <factory>*, *cat\_emb\_dim: int = 1*, *n\_independent: int = 2*, *n\_shared: int = 2*, *epsilon: float = 1e-15*, *momentum: float = 0.02*, *lambda\_sparse: float = 0.001*, *seed: int = 0*, *clip\_value: int = 1*, *verbose: int = 1*, *optimizer\_fn: Any = <class 'torch.optim.adam.Adam'>*, *optimizer\_params: Dict = <factory>*, *scheduler\_fn: Any = None*, *scheduler\_params: Dict = <factory>*, *mask\_type: str = 'sparsemax'*, *input\_dim: int = None*, *output\_dim: int = None*, *device\_name: str = 'auto'*, *n\_shared\_decoder: int = 1*, *n\_indep\_decoder: int = 1*, *grouped\_features: List[List[int]] = <factory>*)[source]¶
:   Bases: `pytorch_tabnet.abstract_model.TabModel`

    `cat_dims` *= None*¶

    `cat_idxs` *= None*¶

    `compute_loss`(*y\_pred*, *y\_true*)[source]¶
    :   Computes the loss according to network output and targets

        Parameters
        :   - **y\_pred** (*list of tensors*) – Output of network
            - **y\_true** (*LongTensor*) – Targets label encoded

        Returns
        :   **loss** – output of loss function(s)

        Return type
        :   torch.Tensor

    `grouped_features` *= None*¶

    `optimizer_params` *= None*¶

    `predict`(*X*)[source]¶
    :   Make predictions on a batch (valid)

        Parameters
        :   **X** (a :tensor: torch.Tensor or matrix: scipy.sparse.csr\_matrix) – Input data

        Returns
        :   **results** – Predictions of the most probable class

        Return type
        :   np.array

    `predict_proba`(*X*)[source]¶
    :   Make predictions for classification on a batch (valid)

        Parameters
        :   **X** (a :tensor: torch.Tensor or matrix: scipy.sparse.csr\_matrix) – Input data

        Returns
        :   **res**

        Return type
        :   list of np.ndarray

    `prepare_target`(*y*)[source]¶
    :   Prepare target before training.

        Parameters
        :   **y** (a :tensor: torch.Tensor) – Target matrix.

        Returns
        :   Converted target matrix.

        Return type
        :   torch.Tensor

    `scheduler_params` *= None*¶

    `stack_batches`(*list\_y\_true*, *list\_y\_score*)[source]¶

    `update_fit_params`(*X\_train*, *y\_train*, *eval\_set*, *weights*)[source]¶
    :   Set attributes relative to fit function.

        Parameters
        :   - **X\_train** (*np.ndarray*) – Train set
            - **y\_train** (*np.array*) – Train targets
            - **eval\_set** (*list of tuple*) – List of eval tuple set (X, y).
            - **weights** (*bool* *or* *dictionnary*) – 0 for no balancing
              1 for automated balancing

## pytorch\_tabnet.multiclass\_utils module¶

### Multi-class / multi-label utility function¶

`pytorch_tabnet.multiclass_utils.``assert_all_finite`(*X*, *allow\_nan=False*)[source]¶
:   Throw a ValueError if X contains NaN or infinity.

    Parameters
    :   - **X** (*array* *or* *sparse matrix*) –
        - **allow\_nan** (*bool*) –

`pytorch_tabnet.multiclass_utils.``check_classification_targets`(*y*)[source]¶
:   Ensure that target y is of a non-regression type.

    Only the following target types (as defined in type\_of\_target) are allowed:
    :   ‘binary’, ‘multiclass’, ‘multiclass-multioutput’,
        ‘multilabel-indicator’, ‘multilabel-sequences’

    Parameters
    :   **y** (*array-like*) –

`pytorch_tabnet.multiclass_utils.``check_output_dim`(*labels*, *y*)[source]¶

`pytorch_tabnet.multiclass_utils.``check_unique_type`(*y*)[source]¶

`pytorch_tabnet.multiclass_utils.``infer_multitask_output`(*y\_train*)[source]¶
:   Infer output\_dim from targets
    This is for multiple tasks.

    Parameters
    :   **y\_train** (*np.ndarray*) – Training targets

    Returns
    :   - **tasks\_dims** (*list*) – Number of classes for output
        - **tasks\_labels** (*list*) – List of sorted list of initial classes

`pytorch_tabnet.multiclass_utils.``infer_output_dim`(*y\_train*)[source]¶
:   Infer output\_dim from targets

    Parameters
    :   **y\_train** (*np.array*) – Training targets

    Returns
    :   - **output\_dim** (*int*) – Number of classes for output
        - **train\_labels** (*list*) – Sorted list of initial classes

`pytorch_tabnet.multiclass_utils.``is_multilabel`(*y*)[source]¶
:   Check if `y` is in a multilabel format.

    Parameters
    :   **y** (*numpy array of shape* *[**n\_samples**]*) – Target values.

    Returns
    :   **out** – Return `True`, if `y` is in a multilabel format, else `` `False ``.

    Return type
    :   bool

    Examples

    ```
    >>> import numpy as np
    >>> from sklearn.utils.multiclass import is_multilabel
    >>> is_multilabel([0, 1, 0, 1])
    False
    >>> is_multilabel([[1], [0, 2], []])
    False
    >>> is_multilabel(np.array([[1, 0], [0, 0]]))
    True
    >>> is_multilabel(np.array([[1], [0], [0]]))
    False
    >>> is_multilabel(np.array([[1, 0, 0]]))
    True
    ```

`pytorch_tabnet.multiclass_utils.``type_of_target`(*y*)[source]¶
:   Determine the type of data indicated by the target.

    Note that this type is the most specific type that can be inferred.
    For example:

    > - `binary` is more specific but compatible with `multiclass`.
    > - `multiclass` of integers is more specific but compatible with
    >   `continuous`.
    > - `multilabel-indicator` is more specific but compatible with
    >   `multiclass-multioutput`.

    Parameters
    :   **y** (*array-like*) –

    Returns
    :   **target\_type** – One of:

        - ’continuous’: y is an array-like of floats that are not all
          integers, and is 1d or a column vector.
        - ’continuous-multioutput’: y is a 2d array of floats that are
          not all integers, and both dimensions are of size > 1.
        - ’binary’: y contains <= 2 discrete values and is 1d or a column
          vector.
        - ’multiclass’: y contains more than two discrete values, is not a
          sequence of sequences, and is 1d or a column vector.
        - ’multiclass-multioutput’: y is a 2d array that contains more
          than two discrete values, is not a sequence of sequences, and both
          dimensions are of size > 1.
        - ’multilabel-indicator’: y is a label indicator matrix, an array
          of two dimensions with at least two columns, and at most 2 unique
          values.
        - ’unknown’: y is array-like but none of the above, such as a 3d
          array, sequence of sequences, or an array of non-sequence objects.

    Return type
    :   string

    Examples

    ```
    >>> import numpy as np
    >>> type_of_target([0.1, 0.6])
    'continuous'
    >>> type_of_target([1, -1, -1, 1])
    'binary'
    >>> type_of_target(['a', 'b', 'a'])
    'binary'
    >>> type_of_target([1.0, 2.0])
    'binary'
    >>> type_of_target([1, 0, 2])
    'multiclass'
    >>> type_of_target([1.0, 0.0, 3.0])
    'multiclass'
    >>> type_of_target(['a', 'b', 'c'])
    'multiclass'
    >>> type_of_target(np.array([[1, 2], [3, 1]]))
    'multiclass-multioutput'
    >>> type_of_target([[1, 2]])
    'multiclass-multioutput'
    >>> type_of_target(np.array([[1.5, 2.0], [3.0, 1.6]]))
    'continuous-multioutput'
    >>> type_of_target(np.array([[0, 1], [1, 1]]))
    'multilabel-indicator'
    ```

`pytorch_tabnet.multiclass_utils.``unique_labels`(*\*ys*)[source]¶
:   Extract an ordered array of unique labels

    We don’t allow:
    :   - mix of multilabel and multiclass (single label) targets
        - mix of label indicator matrix and anything else,
          because there are no explicit labels)
        - mix of label indicator matrices of different sizes
        - mix of string and integer labels

    At the moment, we also don’t allow “multiclass-multioutput” input type.

    Parameters
    :   **\*ys** (*array-likes*) –

    Returns
    :   **out** – An ordered array of unique labels.

    Return type
    :   numpy array of shape [n\_unique\_labels]

    Examples

    ```
    >>> from sklearn.utils.multiclass import unique_labels
    >>> unique_labels([3, 5, 5, 5, 7, 7])
    array([3, 5, 7])
    >>> unique_labels([1, 2, 3, 4], [2, 2, 3, 4])
    array([1, 2, 3, 4])
    >>> unique_labels([1, 2, 10], [5, 11])
    array([ 1,  2,  5, 10, 11])
    ```

Previous

---

© Copyright 2019, Dreamquark

Built with Sphinx using a
theme
provided by Read the Docs.
