# Supplementary material for: Development of a deep learning model for predicting recurrence of hepatocellular carcinoma after liver transplantation
Source: Front Med (Lausanne). 2024 Jun 11;11:1373005. doi: 10.3389/fmed.2024.1373005 (PMC11196752; doi:10.3389/fmed.2024.1373005)
Supplement: Supplementary file 1 [file Data_Sheet_1.ZIP › Raw data/source data and codes/codes/tabnet/docs/generated_docs/README.html]

README — pytorch\_tabnet documentation


pytorch\_tabnet

Contents:

- README
- TabNet : Attentive Interpretable Tabular Learning
- Installation
  - Easy installation
  - Source code
    - CPU only
    - GPU
- What is new ?
- Contributing
- What problems does pytorch-tabnet handle?
- How to use it?
  - Default eval\_metric
  - Custom evaluation metrics
- Semi-supervised pre-training
- Data augmentation on the fly
- Easy saving and loading
- Useful links
  - Model parameters
  - Fit parameters
- pytorch\_tabnet package

pytorch\_tabnet

- »
- README
- View page source

---

# README¶

# TabNet : Attentive Interpretable Tabular Learning¶

This is a pyTorch implementation of Tabnet (Arik, S. O., & Pfister, T. (2019). TabNet: Attentive Interpretable Tabular Learning. arXiv preprint arXiv:1908.07442.) https://arxiv.org/pdf/1908.07442.pdf. Please note that some different choices have been made overtime to improve the library which can differ from the orginal paper.

Any questions ? Want to contribute ? To talk with us ? You can join us on Slack

# Installation¶

## Easy installation¶

You can install using `pip` or `conda` as follows.

**with pip**

```
pip install pytorch-tabnet
```

**with conda**

```
conda install -c conda-forge pytorch-tabnet
```

## Source code¶

If you wan to use it locally within a docker container:

- `git clone git@github.com:dreamquark-ai/tabnet.git`
- `cd tabnet` to get inside the repository

---

### CPU only¶

- `make start` to build and get inside the container

### GPU¶

- `make start-gpu` to build and get inside the GPU container

---

- `poetry install` to install all the dependencies, including jupyter
- `make notebook` inside the same terminal. You can then follow the link to a jupyter notebook with tabnet installed.

# What is new ?¶

- from version **> 4.0** attention is now embedding aware. This aims to maintain a good attention mechanism even with large number of embedding. It is also now possible to specify attention groups (using `grouped_features`). Attention is now done at the group level and not feature level. This is especially useful if a dataset has a lot of columns coming from on single source of data (exemple: a text column transformed using TD-IDF).

# Contributing¶

When contributing to the TabNet repository, please make sure to first discuss the change you wish to make via a new or already existing issue.

Our commits follow the rules presented here.

# What problems does pytorch-tabnet handle?¶

- TabNetClassifier : binary classification and multi-class classification problems
- TabNetRegressor : simple and multi-task regression problems
- TabNetMultiTaskClassifier: multi-task multi-classification problems

# How to use it?¶

TabNet is now scikit-compatible, training a TabNetClassifier or TabNetRegressor is really easy.

```
from pytorch_tabnet.tab_model import TabNetClassifier, TabNetRegressor

clf = TabNetClassifier()  #TabNetRegressor()
clf.fit(
  X_train, Y_train,
  eval_set=[(X_valid, y_valid)]
)
preds = clf.predict(X_test)
```

or for TabNetMultiTaskClassifier :

```
from pytorch_tabnet.multitask import TabNetMultiTaskClassifier
clf = TabNetMultiTaskClassifier()
clf.fit(
  X_train, Y_train,
  eval_set=[(X_valid, y_valid)]
)
preds = clf.predict(X_test)
```

The targets on `y_train/y_valid` should contain a unique type (e.g. they must all be strings or integers).

## Default eval\_metric¶

A few classic evaluation metrics are implemented (see further below for custom ones):

- binary classification metrics : ‘auc’, ‘accuracy’, ‘balanced\_accuracy’, ‘logloss’
- multiclass classification : ‘accuracy’, ‘balanced\_accuracy’, ‘logloss’
- regression: ‘mse’, ‘mae’, ‘rmse’, ‘rmsle’

Important Note : ‘rmsle’ will automatically clip negative predictions to 0, because the model can predict negative values.
In order to match the given scores, you need to use `np.clip(clf.predict(X_predict), a_min=0, a_max=None)` when doing predictions.

## Custom evaluation metrics¶

You can create a metric for your specific need. Here is an example for gini score (note that you need to specifiy whether this metric should be maximized or not):

```
from pytorch_tabnet.metrics import Metric
from sklearn.metrics import roc_auc_score

class Gini(Metric):
    def __init__(self):
        self._name = "gini"
        self._maximize = True

    def __call__(self, y_true, y_score):
        auc = roc_auc_score(y_true, y_score[:, 1])
        return max(2*auc - 1, 0.)

clf = TabNetClassifier()
clf.fit(
  X_train, Y_train,
  eval_set=[(X_valid, y_valid)],
  eval_metric=[Gini]
)
```

A specific customization example notebook is available here : https://github.com/dreamquark-ai/tabnet/blob/develop/customizing\_example.ipynb

# Semi-supervised pre-training¶

Added later to TabNet’s original paper, semi-supervised pre-training is now available via the class `TabNetPretrainer`:

```
# TabNetPretrainer
unsupervised_model = TabNetPretrainer(
    optimizer_fn=torch.optim.Adam,
    optimizer_params=dict(lr=2e-2),
    mask_type='entmax' # "sparsemax"
)

unsupervised_model.fit(
    X_train=X_train,
    eval_set=[X_valid],
    pretraining_ratio=0.8,
)

clf = TabNetClassifier(
    optimizer_fn=torch.optim.Adam,
    optimizer_params=dict(lr=2e-2),
    scheduler_params={"step_size":10, # how to use learning rate scheduler
                      "gamma":0.9},
    scheduler_fn=torch.optim.lr_scheduler.StepLR,
    mask_type='sparsemax' # This will be overwritten if using pretrain model
)

clf.fit(
    X_train=X_train, y_train=y_train,
    eval_set=[(X_train, y_train), (X_valid, y_valid)],
    eval_name=['train', 'valid'],
    eval_metric=['auc'],
    from_unsupervised=unsupervised_model
)
```

The loss function has been normalized to be independent of `pretraining_ratio`, `batch_size` and the number of features in the problem.
A self supervised loss greater than 1 means that your model is reconstructing worse than predicting the mean for each feature, a loss bellow 1 means that the model is doing better than predicting the mean.

A complete example can be found within the notebook `pretraining_example.ipynb`.

/!\ : current implementation is trying to reconstruct the original inputs, but Batch Normalization applies a random transformation that can’t be deduced by a single line, making the reconstruction harder. Lowering the `batch_size` might make the pretraining easier.

# Data augmentation on the fly¶

It is now possible to apply custom data augmentation pipeline during training.
Templates for ClassificationSMOTE and RegressionSMOTE have been added in `pytorch-tabnet/augmentations.py` and can be used as is.

# Easy saving and loading¶

It’s really easy to save and re-load a trained model, this makes TabNet production ready.

```
# save tabnet model
saving_path_name = "./tabnet_model_test_1"
saved_filepath = clf.save_model(saving_path_name)

# define new model with basic parameters and load state dict weights
loaded_clf = TabNetClassifier()
loaded_clf.load_model(saved_filepath)
```

# Useful links¶

- explanatory video
- binary classification examples
- multi-class classification examples
- regression examples
- multi-task regression examples
- multi-task multi-class classification examples
- kaggle moa 1st place solution using tabnet

## Model parameters¶

- `n_d` : int (default=8)

  Width of the decision prediction layer. Bigger values gives more capacity to the model with the risk of overfitting.
  Values typically range from 8 to 64.
- `n_a`: int (default=8)

  Width of the attention embedding for each mask.
  According to the paper n\_d=n\_a is usually a good choice. (default=8)
- `n_steps` : int (default=3)

  Number of steps in the architecture (usually between 3 and 10)
- `gamma` : float (default=1.3)

  This is the coefficient for feature reusage in the masks.
  A value close to 1 will make mask selection least correlated between layers.
  Values range from 1.0 to 2.0.
- `cat_idxs` : list of int (default=[] - Mandatory for embeddings)

  List of categorical features indices.
- `cat_dims` : list of int (default=[] - Mandatory for embeddings)

  List of categorical features number of modalities (number of unique values for a categorical feature)
  /!\ no new modalities can be predicted
- `cat_emb_dim` : list of int (optional)

  List of embeddings size for each categorical features. (default =1)
- `n_independent` : int (default=2)

  Number of independent Gated Linear Units layers at each step.
  Usual values range from 1 to 5.
- `n_shared` : int (default=2)

  Number of shared Gated Linear Units at each step
  Usual values range from 1 to 5
- `epsilon` : float (default 1e-15)

  Should be left untouched.
- `seed` : int (default=0)

  Random seed for reproducibility
- `momentum` : float

  Momentum for batch normalization, typically ranges from 0.01 to 0.4 (default=0.02)
- `clip_value` : float (default None)

  If a float is given this will clip the gradient at clip\_value.
- `lambda_sparse` : float (default = 1e-3)

  This is the extra sparsity loss coefficient as proposed in the original paper. The bigger this coefficient is, the sparser your model will be in terms of feature selection. Depending on the difficulty of your problem, reducing this value could help.
- `optimizer_fn` : torch.optim (default=torch.optim.Adam)

  Pytorch optimizer function
- `optimizer_params`: dict (default=dict(lr=2e-2))

  Parameters compatible with optimizer\_fn used initialize the optimizer. Since we have Adam as our default optimizer, we use this to define the initial learning rate used for training. As mentionned in the original paper, a large initial learning rate of `0.02`  with decay is a good option.
- `scheduler_fn` : torch.optim.lr\_scheduler (default=None)

  Pytorch Scheduler to change learning rates during training.
- `scheduler_params` : dict

  Dictionnary of parameters to apply to the scheduler\_fn. Ex : {”gamma”: 0.95, “step\_size”: 10}
- `model_name` : str (default = ‘DreamQuarkTabNet’)

  Name of the model used for saving in disk, you can customize this to easily retrieve and reuse your trained models.
- `verbose` : int (default=1)

  Verbosity for notebooks plots, set to 1 to see every epoch, 0 to get None.
- `device_name` : str (default=’auto’)
  ‘cpu’ for cpu training, ‘gpu’ for gpu training, ‘auto’ to automatically detect gpu.
- `mask_type: str` (default=’sparsemax’)
  Either “sparsemax” or “entmax” : this is the masking function to use for selecting features.
- `grouped_features: list of list of ints` (default=None)
  This allows the model to share it’s attention accross feature inside a same group.
  This can be especially useful when your preprocessing generates correlated or dependant features: like if you use a TF-IDF or a PCA on a text column.
  Note that feature importance will be exactly the same between features on a same group.
  Please also note that embeddings generated for a categorical variable are always inside a same group.
- `n_shared_decoder` : int (default=1)

  Number of shared GLU block in decoder, this is only useful for `TabNetPretrainer`.
- `n_indep_decoder` : int (default=1)

  Number of independent GLU block in decoder, this is only useful for `TabNetPretrainer`.

## Fit parameters¶

- `X_train` : np.array or scipy.sparse.csr\_matrix

  Training features
- `y_train` : np.array

  Training targets
- `eval_set`: list of tuple

  List of eval tuple set (X, y).  
  The last one is used for early stopping
- `eval_name`: list of str  
  List of eval set names.
- `eval_metric` : list of str  
  List of evaluation metrics.  
  The last metric is used for early stopping.
- `max_epochs` : int (default = 200)

  Maximum number of epochs for trainng.
- `patience` : int (default = 10)

  Number of consecutive epochs without improvement before performing early stopping.

  If patience is set to 0, then no early stopping will be performed.

  Note that if patience is enabled, then best weights from best epoch will automatically be loaded at the end of `fit`.
- `weights` : int or dict (default=0)

  /!\ Only for TabNetClassifier
  Sampling parameter
  0 : no sampling
  1 : automated sampling with inverse class occurrences
  dict : keys are classes, values are weights for each class
- `loss_fn` : torch.loss or list of torch.loss

  Loss function for training (default to mse for regression and cross entropy for classification)
  When using TabNetMultiTaskClassifier you can set a list of same length as number of tasks,
  each task will be assigned its own loss function
- `batch_size` : int (default=1024)

  Number of examples per batch. Large batch sizes are recommended.
- `virtual_batch_size` : int (default=128)

  Size of the mini batches used for “Ghost Batch Normalization”.
  /!\ `virtual_batch_size` should divide `batch_size`
- `num_workers` : int (default=0)

  Number or workers used in torch.utils.data.Dataloader
- `drop_last` : bool (default=False)

  Whether to drop last batch if not complete during training
- `callbacks` : list of callback function  
  List of custom callbacks
- `pretraining_ratio` : float

  ```
    /!\ TabNetPretrainer Only : Percentage of input features to mask during pretraining.

    Should be between 0 and 1. The bigger the harder the reconstruction task is.
  ```
- `warm_start` : bool (default=False)
  In order to match scikit-learn API, this is set to False.
  It allows to fit twice the same model and start from a warm start.
- `compute_importance` : bool (default=True)

  Whether to compute feature importance

Next 
 Previous

---

© Copyright 2019, Dreamquark

Built with Sphinx using a
theme
provided by Read the Docs.
