# Supplementary material for: Development of a deep learning model for predicting recurrence of hepatocellular carcinoma after liver transplantation
Source: Front Med (Lausanne). 2024 Jun 11;11:1373005. doi: 10.3389/fmed.2024.1373005 (PMC11196752; doi:10.3389/fmed.2024.1373005)
Supplement: Supplementary file 1 [file Data_Sheet_1.ZIP › Raw data/source data and codes/codes/tabnet/docs/genindex.html]

Index — pytorch\_tabnet documentation


pytorch\_tabnet

Contents:

- README
- TabNet : Attentive Interpretable Tabular Learning
- Installation
- What is new ?
- Contributing
- What problems does pytorch-tabnet handle?
- How to use it?
- Semi-supervised pre-training
- Data augmentation on the fly
- Easy saving and loading
- Useful links
- pytorch\_tabnet package

pytorch\_tabnet

- »
- Index

---

# Index

**A**
| **B**
| **C**
| **D**
| **E**
| **F**
| **G**
| **H**
| **I**
| **L**
| **M**
| **N**
| **O**
| **P**
| **R**
| **S**
| **T**
| **U**
| **V**
| **W**

## A

|  |  |
| --- | --- |
| - Accuracy (class in pytorch\_tabnet.metrics) - append() (pytorch\_tabnet.callbacks.CallbackContainer method) | - assert\_all\_finite() (in module pytorch\_tabnet.multiclass\_utils) - AttentiveTransformer (class in pytorch\_tabnet.tab\_network) - AUC (class in pytorch\_tabnet.metrics) |

## B

|  |  |
| --- | --- |
| - backward() (pytorch\_tabnet.sparsemax.Entmax15Function static method)   - (pytorch\_tabnet.sparsemax.Entmoid15 static method)   - (pytorch\_tabnet.sparsemax.SparsemaxFunction static method) | - BalancedAccuracy (class in pytorch\_tabnet.metrics) |

## C

|  |  |
| --- | --- |
| - Callback (class in pytorch\_tabnet.callbacks) - CallbackContainer (class in pytorch\_tabnet.callbacks) - callbacks (pytorch\_tabnet.callbacks.CallbackContainer attribute) - cat\_dims (pytorch\_tabnet.abstract\_model.TabModel attribute)   - (pytorch\_tabnet.multitask.TabNetMultiTaskClassifier attribute)   - (pytorch\_tabnet.pretraining.TabNetPretrainer attribute)   - (pytorch\_tabnet.tab\_model.TabNetClassifier attribute)   - (pytorch\_tabnet.tab\_model.TabNetRegressor attribute) - cat\_emb\_dim (pytorch\_tabnet.abstract\_model.TabModel attribute) - cat\_idxs (pytorch\_tabnet.abstract\_model.TabModel attribute)   - (pytorch\_tabnet.multitask.TabNetMultiTaskClassifier attribute)   - (pytorch\_tabnet.pretraining.TabNetPretrainer attribute)   - (pytorch\_tabnet.tab\_model.TabNetClassifier attribute)   - (pytorch\_tabnet.tab\_model.TabNetRegressor attribute) - check\_classification\_targets() (in module pytorch\_tabnet.multiclass\_utils) - check\_embedding\_parameters() (in module pytorch\_tabnet.utils) - check\_input() (in module pytorch\_tabnet.utils) | - check\_list\_groups() (in module pytorch\_tabnet.utils) - check\_metrics() (in module pytorch\_tabnet.metrics) - check\_output\_dim() (in module pytorch\_tabnet.multiclass\_utils) - check\_unique\_type() (in module pytorch\_tabnet.multiclass\_utils) - check\_warm\_start() (in module pytorch\_tabnet.utils) - ClassificationSMOTE (class in pytorch\_tabnet.augmentations) - clip\_value (pytorch\_tabnet.abstract\_model.TabModel attribute) - ComplexEncoder (class in pytorch\_tabnet.utils) - compute\_loss() (pytorch\_tabnet.abstract\_model.TabModel method)   - (pytorch\_tabnet.multitask.TabNetMultiTaskClassifier method)   - (pytorch\_tabnet.pretraining.TabNetPretrainer method)   - (pytorch\_tabnet.tab\_model.TabNetClassifier method)   - (pytorch\_tabnet.tab\_model.TabNetRegressor method) - create\_dataloaders() (in module pytorch\_tabnet.pretraining\_utils)   - (in module pytorch\_tabnet.utils) - create\_explain\_matrix() (in module pytorch\_tabnet.utils) - create\_group\_matrix() (in module pytorch\_tabnet.utils) - create\_sampler() (in module pytorch\_tabnet.utils) |

## D

|  |  |
| --- | --- |
| - default() (pytorch\_tabnet.utils.ComplexEncoder method) | - define\_device() (in module pytorch\_tabnet.utils) - device\_name (pytorch\_tabnet.abstract\_model.TabModel attribute) |

## E

|  |  |
| --- | --- |
| - early\_stopping\_metric (pytorch\_tabnet.callbacks.EarlyStopping attribute)   - (pytorch\_tabnet.callbacks.LRSchedulerCallback attribute) - EarlyStopping (class in pytorch\_tabnet.callbacks) - EmbeddingGenerator (class in pytorch\_tabnet.tab\_network) - Entmax15 (class in pytorch\_tabnet.sparsemax) | - entmax15() (in module pytorch\_tabnet.sparsemax) - Entmax15Function (class in pytorch\_tabnet.sparsemax) - Entmoid15 (class in pytorch\_tabnet.sparsemax) - entmoid15() (in module pytorch\_tabnet.sparsemax) - epsilon (pytorch\_tabnet.abstract\_model.TabModel attribute) - explain() (pytorch\_tabnet.abstract\_model.TabModel method) |

## F

|  |  |
| --- | --- |
| - FeatTransformer (class in pytorch\_tabnet.tab\_network) - filter\_weights() (in module pytorch\_tabnet.utils) - fit() (pytorch\_tabnet.abstract\_model.TabModel method)   - (pytorch\_tabnet.pretraining.TabNetPretrainer method) - forward() (pytorch\_tabnet.sparsemax.Entmax15 method)   - (pytorch\_tabnet.sparsemax.Entmax15Function static method)   - (pytorch\_tabnet.sparsemax.Entmoid15 static method)   - (pytorch\_tabnet.sparsemax.Sparsemax method)   - (pytorch\_tabnet.sparsemax.SparsemaxFunction static method)   - (pytorch\_tabnet.tab\_network.AttentiveTransformer method)   - (pytorch\_tabnet.tab\_network.EmbeddingGenerator method)   - (pytorch\_tabnet.tab\_network.FeatTransformer method)   - (pytorch\_tabnet.tab\_network.GBN method)   - (pytorch\_tabnet.tab\_network.GLU\_Block method)   - (pytorch\_tabnet.tab\_network.GLU\_Layer method)   - (pytorch\_tabnet.tab\_network.RandomObfuscator method)   - (pytorch\_tabnet.tab\_network.TabNet method)   - (pytorch\_tabnet.tab\_network.TabNetDecoder method)   - (pytorch\_tabnet.tab\_network.TabNetEncoder method)   - (pytorch\_tabnet.tab\_network.TabNetNoEmbeddings method)   - (pytorch\_tabnet.tab\_network.TabNetPretraining method) | - forward\_masks() (pytorch\_tabnet.tab\_network.TabNet method)   - (pytorch\_tabnet.tab\_network.TabNetEncoder method)   - (pytorch\_tabnet.tab\_network.TabNetNoEmbeddings method)   - (pytorch\_tabnet.tab\_network.TabNetPretraining method) |

## G

|  |  |
| --- | --- |
| - gamma (pytorch\_tabnet.abstract\_model.TabModel attribute) - GBN (class in pytorch\_tabnet.tab\_network) - get\_metrics\_by\_names() (pytorch\_tabnet.metrics.Metric class method) - GLU\_Block (class in pytorch\_tabnet.tab\_network) - GLU\_Layer (class in pytorch\_tabnet.tab\_network) | - grouped\_features (pytorch\_tabnet.abstract\_model.TabModel attribute)   - (pytorch\_tabnet.multitask.TabNetMultiTaskClassifier attribute)   - (pytorch\_tabnet.pretraining.TabNetPretrainer attribute)   - (pytorch\_tabnet.tab\_model.TabNetClassifier attribute)   - (pytorch\_tabnet.tab\_model.TabNetRegressor attribute) |

## H

|  |
| --- |
| - History (class in pytorch\_tabnet.callbacks) |

## I

|  |  |
| --- | --- |
| - infer\_multitask\_output() (in module pytorch\_tabnet.multiclass\_utils) - infer\_output\_dim() (in module pytorch\_tabnet.multiclass\_utils) - initialize\_glu() (in module pytorch\_tabnet.tab\_network) - initialize\_non\_glu() (in module pytorch\_tabnet.tab\_network) | - input\_dim (pytorch\_tabnet.abstract\_model.TabModel attribute) - is\_batch\_level (pytorch\_tabnet.callbacks.LRSchedulerCallback attribute) - is\_maximize (pytorch\_tabnet.callbacks.EarlyStopping attribute) - is\_multilabel() (in module pytorch\_tabnet.multiclass\_utils) |

## L

|  |  |
| --- | --- |
| - lambda\_sparse (pytorch\_tabnet.abstract\_model.TabModel attribute) - load\_class\_attrs() (pytorch\_tabnet.abstract\_model.TabModel method) - load\_model() (pytorch\_tabnet.abstract\_model.TabModel method) | - load\_weights\_from\_unsupervised() (pytorch\_tabnet.abstract\_model.TabModel method) - LogLoss (class in pytorch\_tabnet.metrics) - LRSchedulerCallback (class in pytorch\_tabnet.callbacks) |

## M

|  |  |
| --- | --- |
| - MAE (class in pytorch\_tabnet.metrics) - mask\_type (pytorch\_tabnet.abstract\_model.TabModel attribute) - Metric (class in pytorch\_tabnet.metrics) - metric\_names (pytorch\_tabnet.metrics.MetricContainer attribute)   - (pytorch\_tabnet.metrics.UnsupMetricContainer attribute) | - MetricContainer (class in pytorch\_tabnet.metrics) - momentum (pytorch\_tabnet.abstract\_model.TabModel attribute) - MSE (class in pytorch\_tabnet.metrics) |

## N

|  |  |
| --- | --- |
| - n\_a (pytorch\_tabnet.abstract\_model.TabModel attribute) - n\_d (pytorch\_tabnet.abstract\_model.TabModel attribute) - n\_indep\_decoder (pytorch\_tabnet.abstract\_model.TabModel attribute) | - n\_independent (pytorch\_tabnet.abstract\_model.TabModel attribute) - n\_shared (pytorch\_tabnet.abstract\_model.TabModel attribute) - n\_shared\_decoder (pytorch\_tabnet.abstract\_model.TabModel attribute) - n\_steps (pytorch\_tabnet.abstract\_model.TabModel attribute) |

## O

|  |  |
| --- | --- |
| - on\_batch\_begin() (pytorch\_tabnet.callbacks.Callback method)   - (pytorch\_tabnet.callbacks.CallbackContainer method) - on\_batch\_end() (pytorch\_tabnet.callbacks.Callback method)   - (pytorch\_tabnet.callbacks.CallbackContainer method)   - (pytorch\_tabnet.callbacks.History method)   - (pytorch\_tabnet.callbacks.LRSchedulerCallback method) - on\_epoch\_begin() (pytorch\_tabnet.callbacks.Callback method)   - (pytorch\_tabnet.callbacks.CallbackContainer method)   - (pytorch\_tabnet.callbacks.History method) - on\_epoch\_end() (pytorch\_tabnet.callbacks.Callback method)   - (pytorch\_tabnet.callbacks.CallbackContainer method)   - (pytorch\_tabnet.callbacks.EarlyStopping method)   - (pytorch\_tabnet.callbacks.History method)   - (pytorch\_tabnet.callbacks.LRSchedulerCallback method) | - on\_train\_begin() (pytorch\_tabnet.callbacks.Callback method)   - (pytorch\_tabnet.callbacks.CallbackContainer method)   - (pytorch\_tabnet.callbacks.History method) - on\_train\_end() (pytorch\_tabnet.callbacks.Callback method)   - (pytorch\_tabnet.callbacks.CallbackContainer method)   - (pytorch\_tabnet.callbacks.EarlyStopping method) - optimizer (pytorch\_tabnet.callbacks.LRSchedulerCallback attribute) - optimizer\_fn (pytorch\_tabnet.abstract\_model.TabModel attribute) - optimizer\_params (pytorch\_tabnet.abstract\_model.TabModel attribute)   - (pytorch\_tabnet.multitask.TabNetMultiTaskClassifier attribute)   - (pytorch\_tabnet.pretraining.TabNetPretrainer attribute)   - (pytorch\_tabnet.tab\_model.TabNetClassifier attribute)   - (pytorch\_tabnet.tab\_model.TabNetRegressor attribute) - output\_dim (pytorch\_tabnet.abstract\_model.TabModel attribute) |

## P

|  |  |
| --- | --- |
| - patience (pytorch\_tabnet.callbacks.EarlyStopping attribute) - predict() (pytorch\_tabnet.abstract\_model.TabModel method)   - (pytorch\_tabnet.multitask.TabNetMultiTaskClassifier method)   - (pytorch\_tabnet.pretraining.TabNetPretrainer method) - predict\_func() (pytorch\_tabnet.tab\_model.TabNetClassifier method)   - (pytorch\_tabnet.tab\_model.TabNetRegressor method) - predict\_proba() (pytorch\_tabnet.multitask.TabNetMultiTaskClassifier method)   - (pytorch\_tabnet.tab\_model.TabNetClassifier method) - PredictDataset (class in pytorch\_tabnet.utils) - prefix (pytorch\_tabnet.metrics.MetricContainer attribute)   - (pytorch\_tabnet.metrics.UnsupMetricContainer attribute) - prepare\_target() (pytorch\_tabnet.abstract\_model.TabModel method)   - (pytorch\_tabnet.multitask.TabNetMultiTaskClassifier method)   - (pytorch\_tabnet.pretraining.TabNetPretrainer method)   - (pytorch\_tabnet.tab\_model.TabNetClassifier method)   - (pytorch\_tabnet.tab\_model.TabNetRegressor method) | - pytorch\_tabnet.abstract\_model (module) - pytorch\_tabnet.augmentations (module) - pytorch\_tabnet.callbacks (module) - pytorch\_tabnet.metrics (module) - pytorch\_tabnet.multiclass\_utils (module) - pytorch\_tabnet.multitask (module) - pytorch\_tabnet.pretraining (module) - pytorch\_tabnet.pretraining\_utils (module) - pytorch\_tabnet.sparsemax (module) - pytorch\_tabnet.tab\_model (module) - pytorch\_tabnet.tab\_network (module) - pytorch\_tabnet.utils (module) |

## R

|  |  |
| --- | --- |
| - RandomObfuscator (class in pytorch\_tabnet.tab\_network) - RegressionSMOTE (class in pytorch\_tabnet.augmentations) | - RMSE (class in pytorch\_tabnet.metrics) - RMSLE (class in pytorch\_tabnet.metrics) |

## S

|  |  |
| --- | --- |
| - save\_model() (pytorch\_tabnet.abstract\_model.TabModel method) - scheduler\_fn (pytorch\_tabnet.abstract\_model.TabModel attribute)   - (pytorch\_tabnet.callbacks.LRSchedulerCallback attribute) - scheduler\_params (pytorch\_tabnet.abstract\_model.TabModel attribute)   - (pytorch\_tabnet.callbacks.LRSchedulerCallback attribute)   - (pytorch\_tabnet.multitask.TabNetMultiTaskClassifier attribute)   - (pytorch\_tabnet.pretraining.TabNetPretrainer attribute)   - (pytorch\_tabnet.tab\_model.TabNetClassifier attribute)   - (pytorch\_tabnet.tab\_model.TabNetRegressor attribute) - seed (pytorch\_tabnet.abstract\_model.TabModel attribute) - set\_params() (pytorch\_tabnet.callbacks.Callback method)   - (pytorch\_tabnet.callbacks.CallbackContainer method) | - set\_trainer() (pytorch\_tabnet.callbacks.Callback method)   - (pytorch\_tabnet.callbacks.CallbackContainer method) - Sparsemax (class in pytorch\_tabnet.sparsemax) - sparsemax() (in module pytorch\_tabnet.sparsemax) - SparsemaxFunction (class in pytorch\_tabnet.sparsemax) - SparsePredictDataset (class in pytorch\_tabnet.utils) - SparseTorchDataset (class in pytorch\_tabnet.utils) - stack\_batches() (pytorch\_tabnet.multitask.TabNetMultiTaskClassifier method)   - (pytorch\_tabnet.pretraining.TabNetPretrainer method)   - (pytorch\_tabnet.tab\_model.TabNetClassifier method)   - (pytorch\_tabnet.tab\_model.TabNetRegressor method) |

## T

|  |  |
| --- | --- |
| - TabModel (class in pytorch\_tabnet.abstract\_model) - TabNet (class in pytorch\_tabnet.tab\_network) - TabNetClassifier (class in pytorch\_tabnet.tab\_model) - TabNetDecoder (class in pytorch\_tabnet.tab\_network) - TabNetEncoder (class in pytorch\_tabnet.tab\_network) - TabNetMultiTaskClassifier (class in pytorch\_tabnet.multitask) - TabNetNoEmbeddings (class in pytorch\_tabnet.tab\_network) - TabNetPretrainer (class in pytorch\_tabnet.pretraining) - TabNetPretraining (class in pytorch\_tabnet.tab\_network) - TabNetRegressor (class in pytorch\_tabnet.tab\_model) - tol (pytorch\_tabnet.callbacks.EarlyStopping attribute) - TorchDataset (class in pytorch\_tabnet.utils) - trainer (pytorch\_tabnet.callbacks.History attribute) - training (pytorch\_tabnet.sparsemax.Entmax15 attribute)   - (pytorch\_tabnet.sparsemax.Sparsemax attribute)   - (pytorch\_tabnet.tab\_network.AttentiveTransformer attribute)   - (pytorch\_tabnet.tab\_network.EmbeddingGenerator attribute)   - (pytorch\_tabnet.tab\_network.FeatTransformer attribute)   - (pytorch\_tabnet.tab\_network.GBN attribute)   - (pytorch\_tabnet.tab\_network.GLU\_Block attribute)   - (pytorch\_tabnet.tab\_network.GLU\_Layer attribute)   - (pytorch\_tabnet.tab\_network.RandomObfuscator attribute)   - (pytorch\_tabnet.tab\_network.TabNet attribute)   - (pytorch\_tabnet.tab\_network.TabNetDecoder attribute)   - (pytorch\_tabnet.tab\_network.TabNetEncoder attribute)   - (pytorch\_tabnet.tab\_network.TabNetNoEmbeddings attribute)   - (pytorch\_tabnet.tab\_network.TabNetPretraining attribute) | - type\_of\_target() (in module pytorch\_tabnet.multiclass\_utils) |

## U

|  |  |
| --- | --- |
| - unique\_labels() (in module pytorch\_tabnet.multiclass\_utils) - UnsupervisedLoss() (in module pytorch\_tabnet.metrics) - UnsupervisedLossNumpy() (in module pytorch\_tabnet.metrics) - UnsupervisedMetric (class in pytorch\_tabnet.metrics) - UnsupervisedNumpyMetric (class in pytorch\_tabnet.metrics) | - UnsupMetricContainer (class in pytorch\_tabnet.metrics) - update\_fit\_params() (pytorch\_tabnet.abstract\_model.TabModel method)   - (pytorch\_tabnet.multitask.TabNetMultiTaskClassifier method)   - (pytorch\_tabnet.pretraining.TabNetPretrainer method)   - (pytorch\_tabnet.tab\_model.TabNetClassifier method)   - (pytorch\_tabnet.tab\_model.TabNetRegressor method) |

## V

|  |  |
| --- | --- |
| - validate\_eval\_set() (in module pytorch\_tabnet.pretraining\_utils)   - (in module pytorch\_tabnet.utils) | - verbose (pytorch\_tabnet.abstract\_model.TabModel attribute)   - (pytorch\_tabnet.callbacks.History attribute) |

## W

|  |
| --- |
| - weight\_updater() (pytorch\_tabnet.tab\_model.TabNetClassifier method) |

---

© Copyright 2019, Dreamquark

Built with Sphinx using a
theme
provided by Read the Docs.
