# Supplementary material for: Development of a deep learning model for predicting recurrence of hepatocellular carcinoma after liver transplantation
Source: Front Med (Lausanne). 2024 Jun 11;11:1373005. doi: 10.3389/fmed.2024.1373005 (PMC11196752; doi:10.3389/fmed.2024.1373005)
Supplement: Supplementary file 1 [file Data_Sheet_1.ZIP › Raw data/source data and codes/codes/tabnet/docs/index.html]

Welcome to pytorch\_tabnet’s documentation! — pytorch\_tabnet documentation


pytorch\_tabnet

Contents:

- README
- TabNet : Attentive Interpretable Tabular Learning
- Installation
- What is new ?
- Contributing
- What problems does pytorch-tabnet handle?
- How to use it?
- Semi-supervised pre-training
- Data augmentation on the fly
- Easy saving and loading
- Useful links
- pytorch\_tabnet package

pytorch\_tabnet

- »
- Welcome to pytorch\_tabnet’s documentation!
- View page source

---

# Welcome to pytorch\_tabnet’s documentation!¶

Contents:

- README
- TabNet : Attentive Interpretable Tabular Learning
- Installation
  - Easy installation
  - Source code
- What is new ?
- Contributing
- What problems does pytorch-tabnet handle?
- How to use it?
  - Default eval\_metric
  - Custom evaluation metrics
- Semi-supervised pre-training
- Data augmentation on the fly
- Easy saving and loading
- Useful links
  - Model parameters
  - Fit parameters
- pytorch\_tabnet package
  - pytorch\_tabnet.pretraining\_utils module
  - pytorch\_tabnet.augmentations module
  - pytorch\_tabnet.tab\_network module
  - pytorch\_tabnet.metrics module
  - pytorch\_tabnet.tab\_model module
  - pytorch\_tabnet.sparsemax module
  - pytorch\_tabnet.callbacks module
  - pytorch\_tabnet.abstract\_model module
  - pytorch\_tabnet.pretraining module
  - pytorch\_tabnet.utils module
  - pytorch\_tabnet.multitask module
  - pytorch\_tabnet.multiclass\_utils module

# Indices and tables¶

- Index
- Module Index
- Search Page

Next

---

© Copyright 2019, Dreamquark

Built with Sphinx using a
theme
provided by Read the Docs.
