# Supplementary material for: Development of a deep learning model for predicting recurrence of hepatocellular carcinoma after liver transplantation
Source: Front Med (Lausanne). 2024 Jun 11;11:1373005. doi: 10.3389/fmed.2024.1373005 (PMC11196752; doi:10.3389/fmed.2024.1373005)
Supplement: Supplementary file 1 [file Data_Sheet_1.ZIP › Raw data/source data and codes/codes/tabnet/docs/py-modindex.html]

Python Module Index — pytorch\_tabnet documentation


pytorch\_tabnet

Contents:

- README
- TabNet : Attentive Interpretable Tabular Learning
- Installation
- What is new ?
- Contributing
- What problems does pytorch-tabnet handle?
- How to use it?
- Semi-supervised pre-training
- Data augmentation on the fly
- Easy saving and loading
- Useful links
- pytorch\_tabnet package

pytorch\_tabnet

- »
- Python Module Index

---

# Python Module Index

**p**

|  |  |  |
| --- | --- | --- |
|  |  |  |
|  | **p** |  |
|  | `pytorch_tabnet` |  |
|  | `pytorch_tabnet.abstract_model` |  |
|  | `pytorch_tabnet.augmentations` |  |
|  | `pytorch_tabnet.callbacks` |  |
|  | `pytorch_tabnet.metrics` |  |
|  | `pytorch_tabnet.multiclass_utils` |  |
|  | `pytorch_tabnet.multitask` |  |
|  | `pytorch_tabnet.pretraining` |  |
|  | `pytorch_tabnet.pretraining_utils` |  |
|  | `pytorch_tabnet.sparsemax` |  |
|  | `pytorch_tabnet.tab_model` |  |
|  | `pytorch_tabnet.tab_network` |  |
|  | `pytorch_tabnet.utils` |  |

---

© Copyright 2019, Dreamquark

Built with Sphinx using a
theme
provided by Read the Docs.
