# Supplementary material for: Development of a deep learning model for predicting recurrence of hepatocellular carcinoma after liver transplantation
Source: Front Med (Lausanne). 2024 Jun 11;11:1373005. doi: 10.3389/fmed.2024.1373005 (PMC11196752; doi:10.3389/fmed.2024.1373005)
Supplement: Supplementary file 1 [file Data_Sheet_1.ZIP › Raw data/source data and codes/codes/tabnet/docs-scripts/source/_templates/layout.html]

{% extends "!layout.html" %}
{% block rootrellink %}- home|
- search|
{% endblock %}
{% block relbar1 %}

# Pytorch Tabnet

{{ super() }}
{% endblock %}
{# put the sidebar before the body #}
{% block sidebar1 %}{{ sidebar() }}{% endblock %}
{% block sidebar2 %}{% endblock %}
