# Supplementary figures and images for: Development of a deep learning model for predicting recurrence of hepatocellular carcinoma after liver transplantation
Source: Front Med (Lausanne). 2024 Jun 11;11:1373005. doi: 10.3389/fmed.2024.1373005 (PMC11196752; doi:10.3389/fmed.2024.1373005)

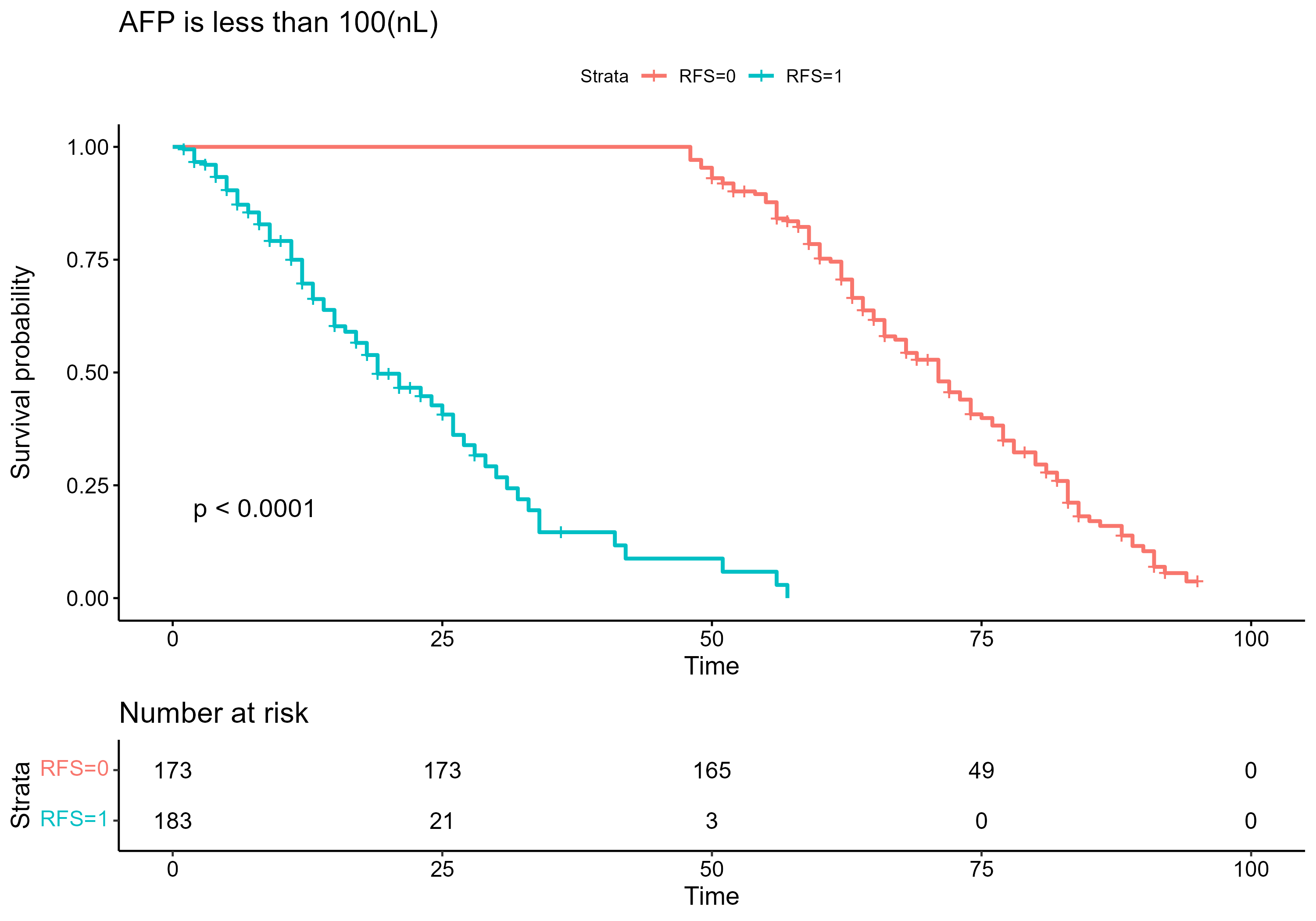

Supplement: Supplementary file 1 [file Data_Sheet_1.ZIP › Raw data/images/AFP is less than 100(nL).png]

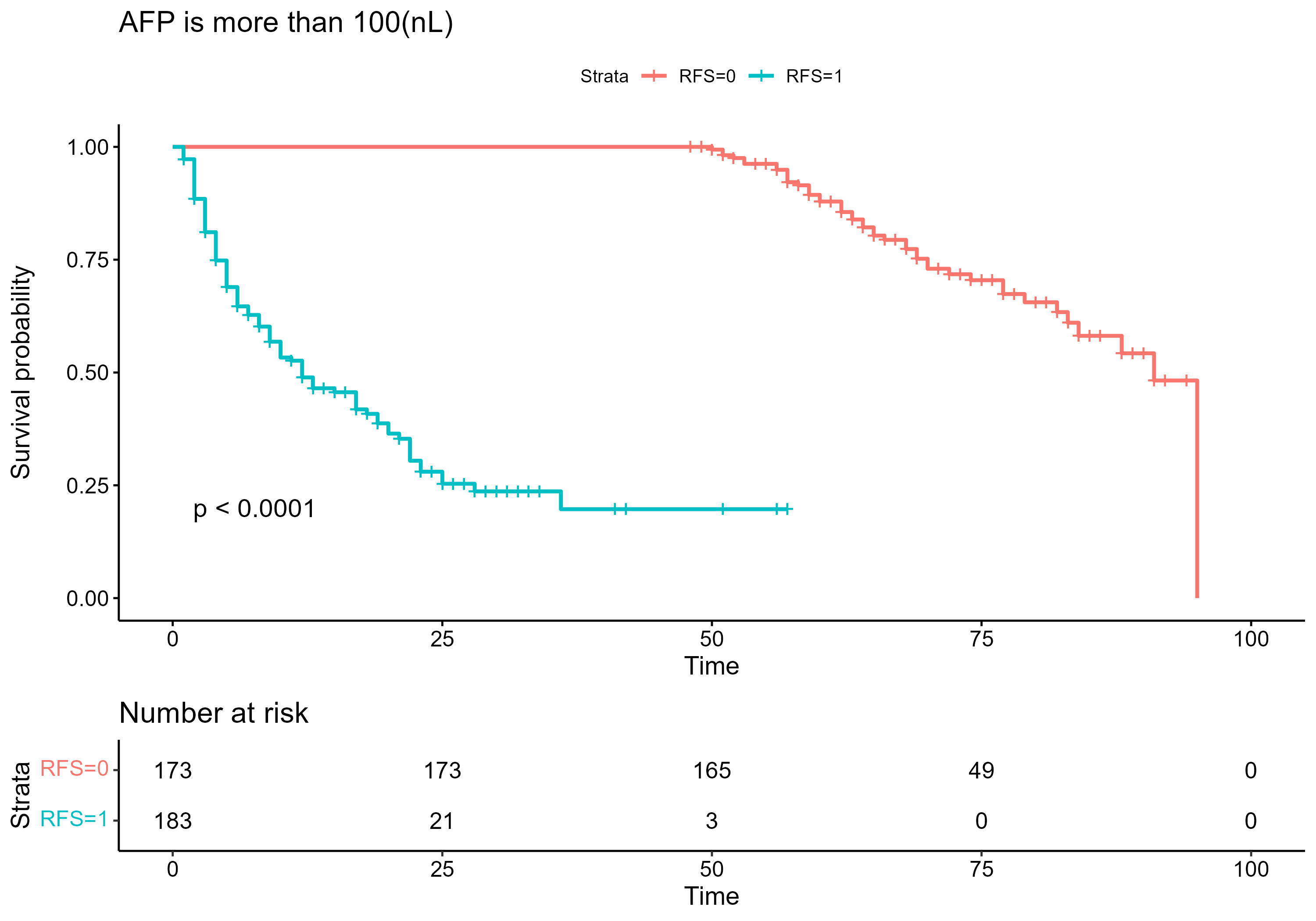

Supplement: Supplementary file 1 [file Data_Sheet_1.ZIP › Raw data/images/AFP is more than 100(nL).png]

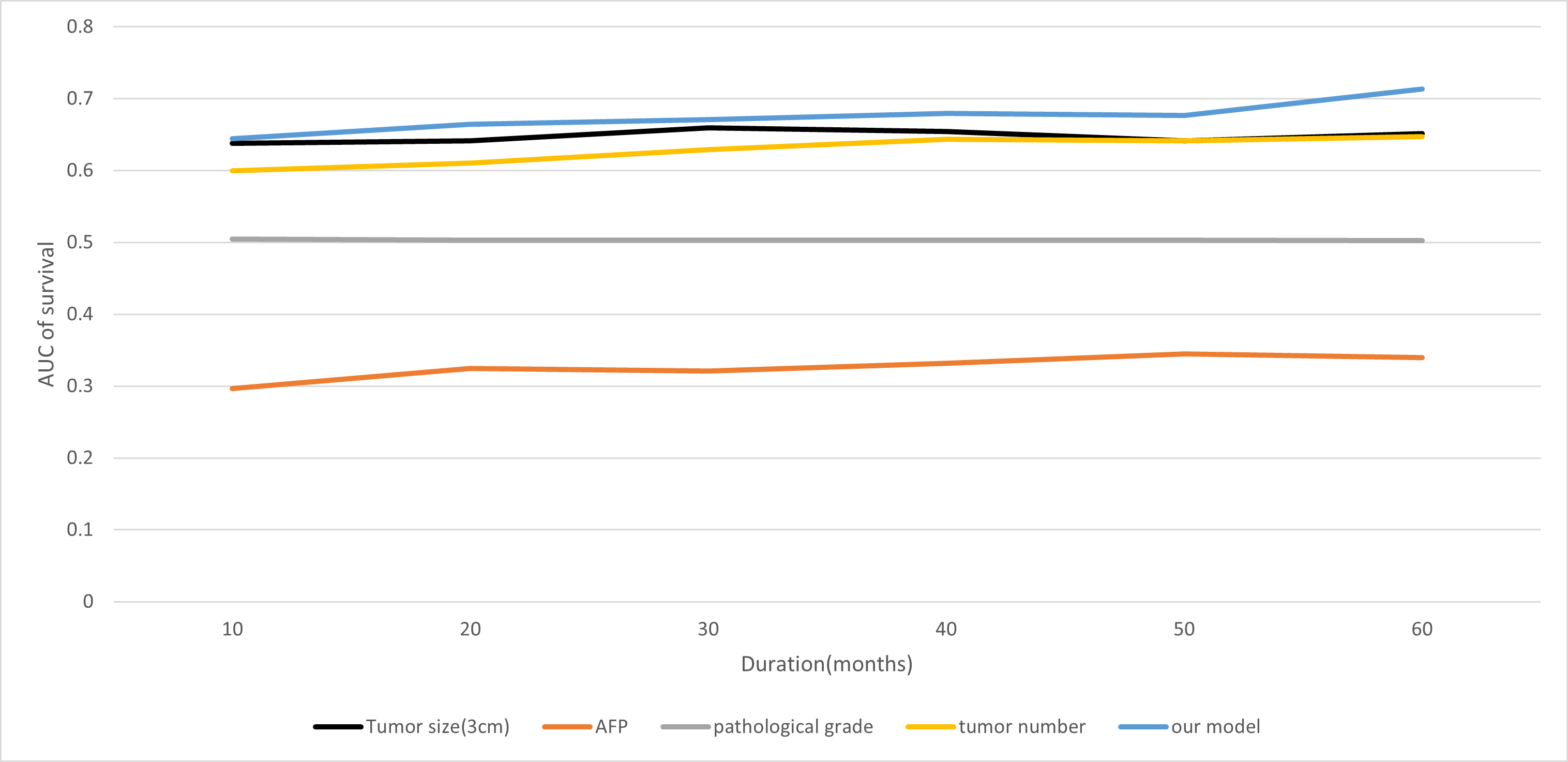

Supplement: Supplementary file 1 [file Data_Sheet_1.ZIP › Raw data/images/AUC.png]

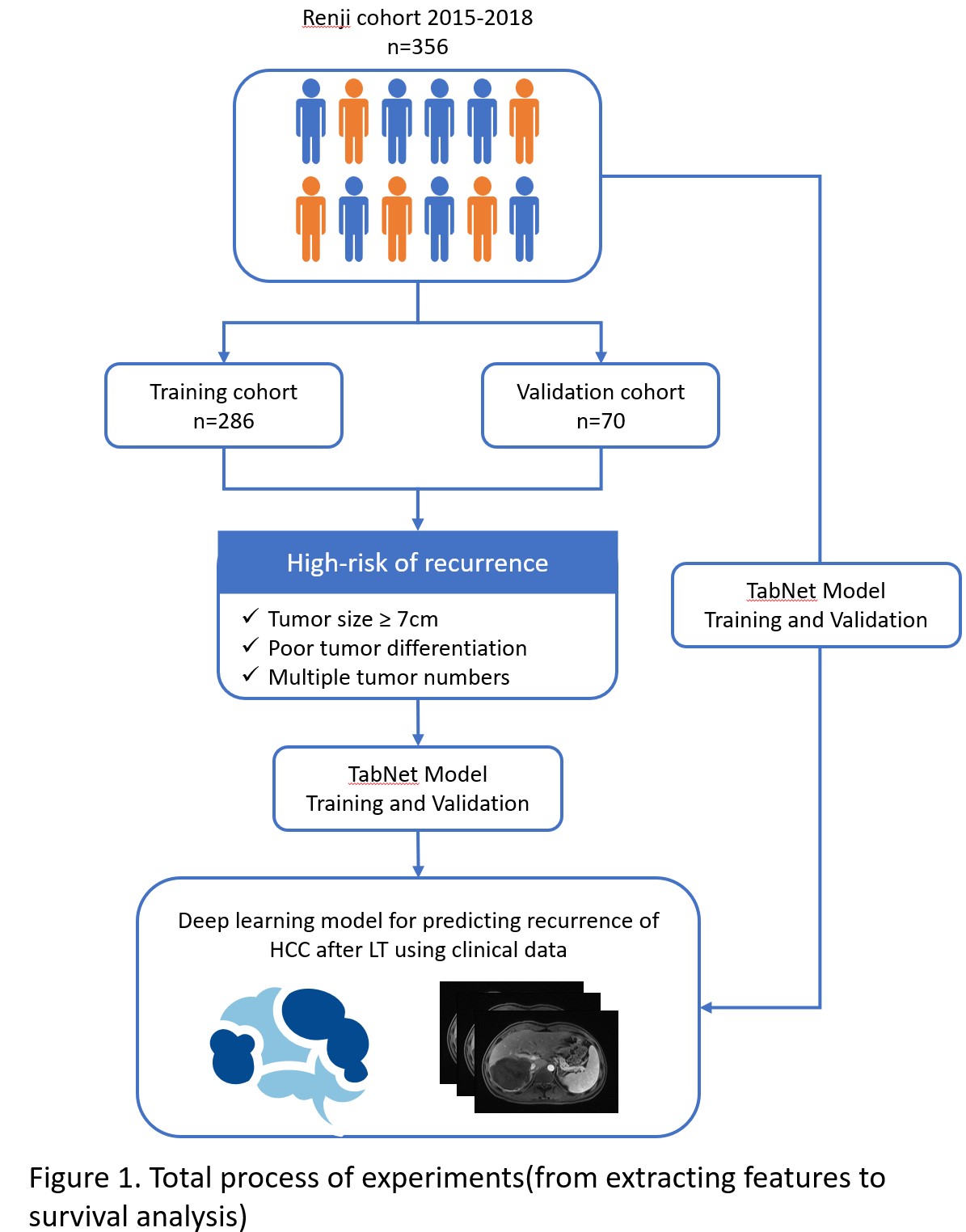

Supplement: Supplementary file 1 [file Data_Sheet_1.ZIP › Raw data/images/figure 1..jpg]

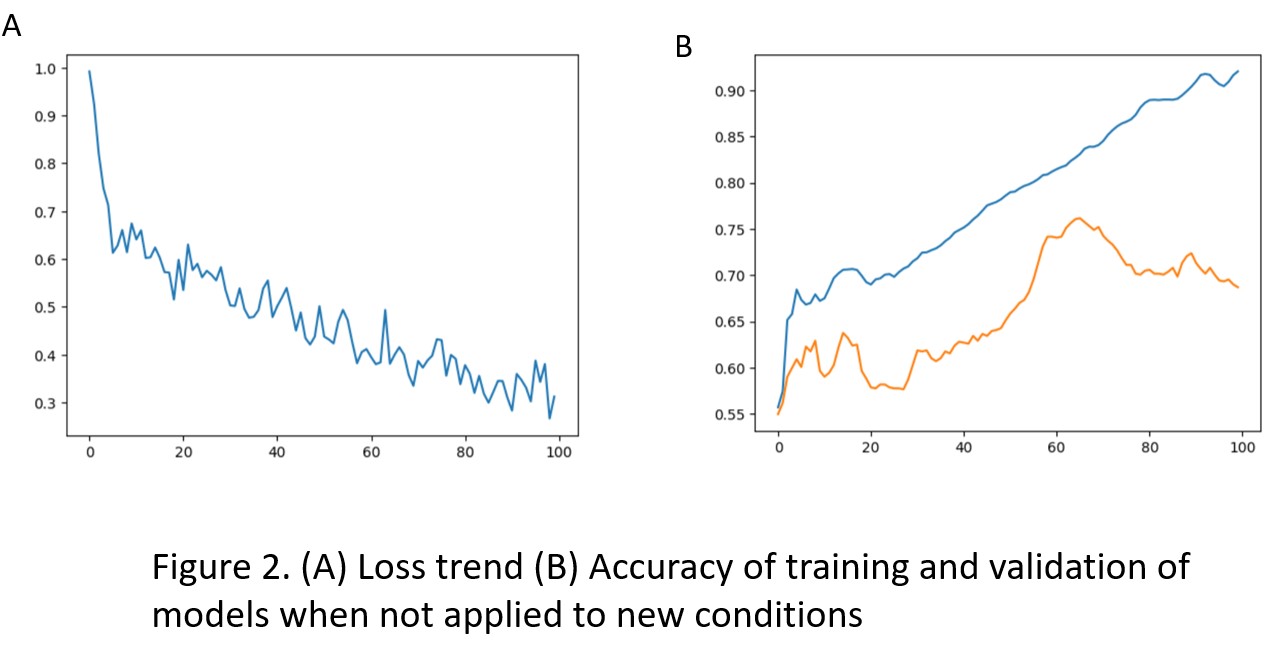

Supplement: Supplementary file 1 [file Data_Sheet_1.ZIP › Raw data/images/figure 2..jpg]

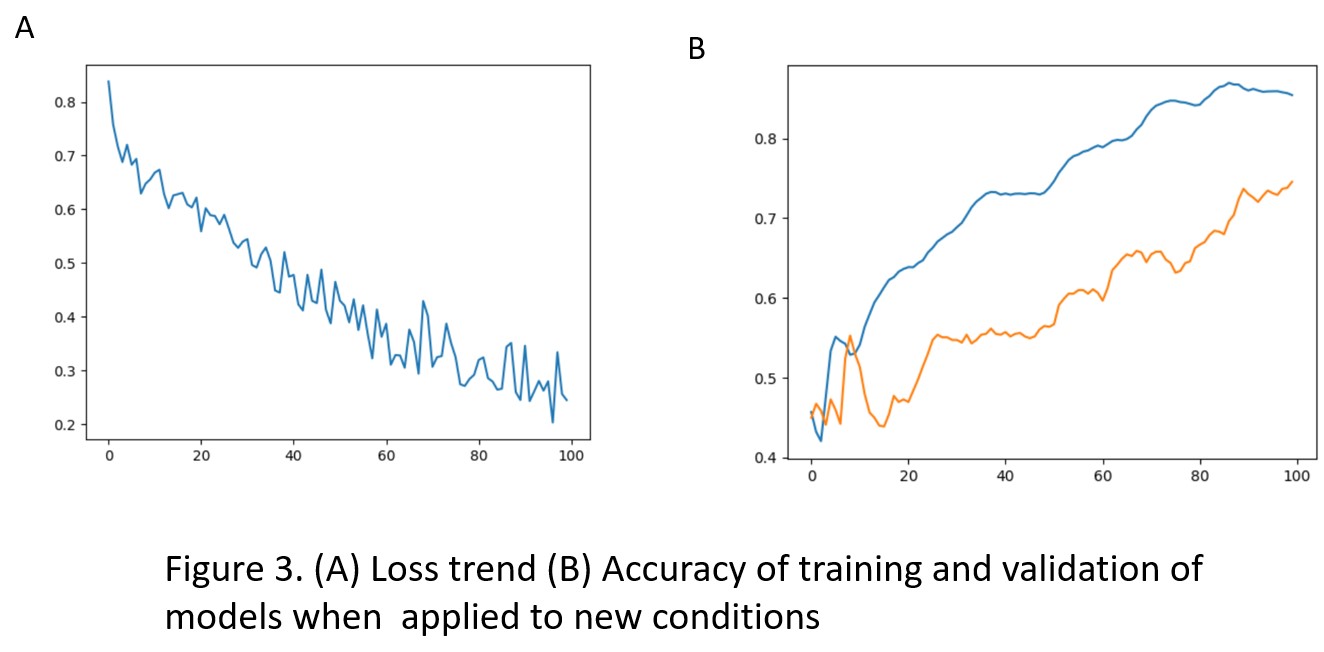

Supplement: Supplementary file 1 [file Data_Sheet_1.ZIP › Raw data/images/figure 3..jpg]

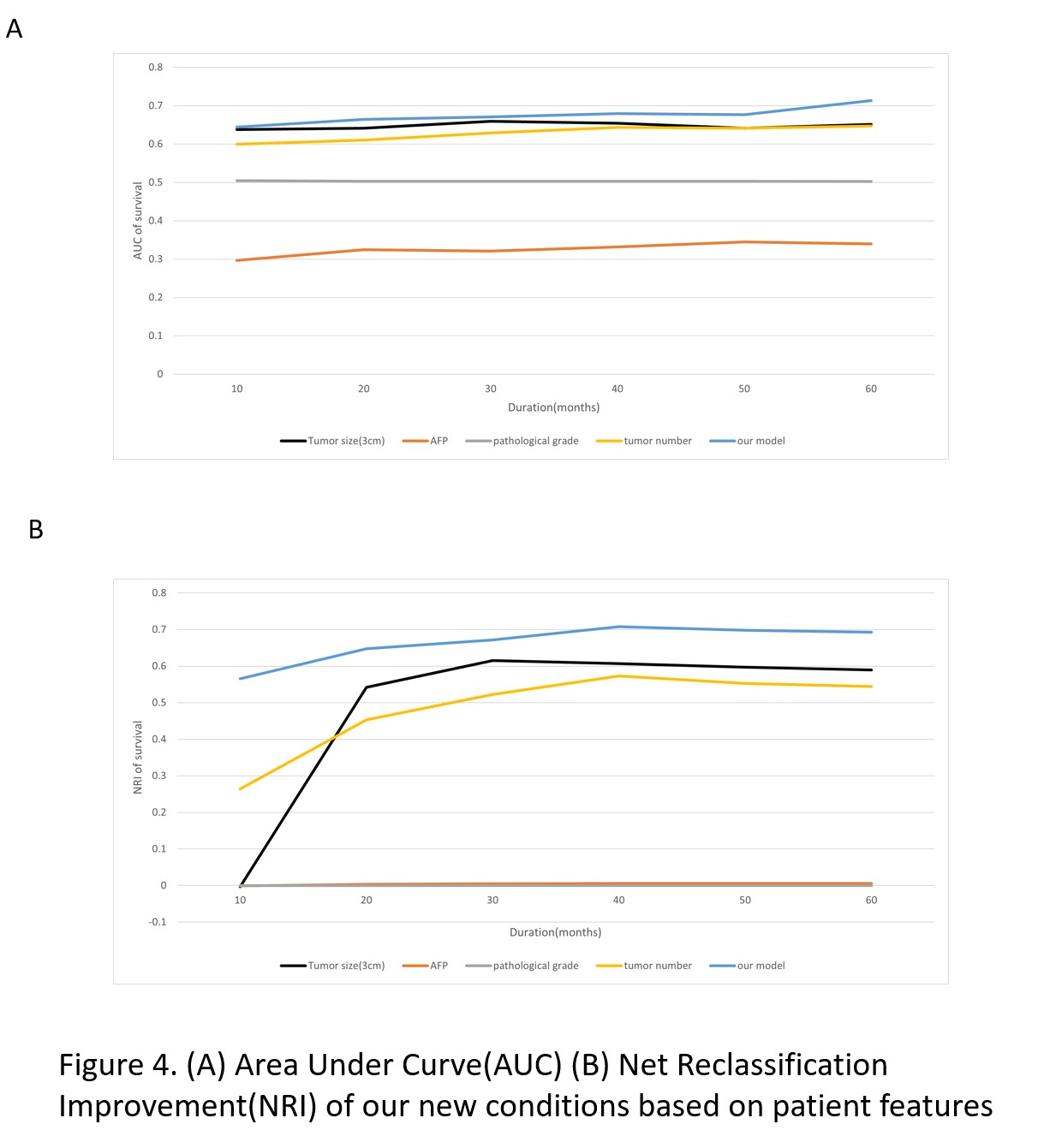

Supplement: Supplementary file 1 [file Data_Sheet_1.ZIP › Raw data/images/figure 4..jpg]

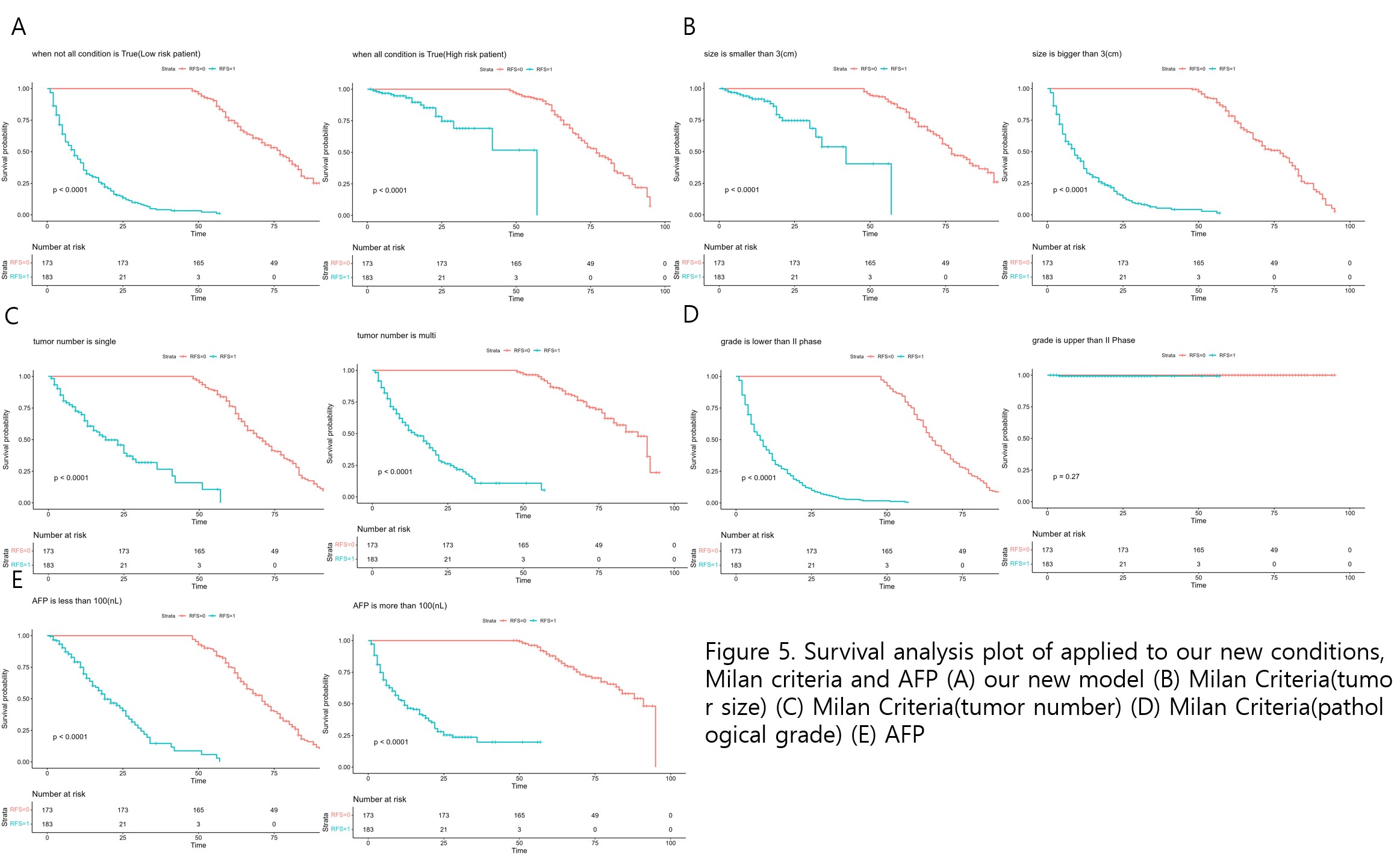

Supplement: Supplementary file 1 [file Data_Sheet_1.ZIP › Raw data/images/figure 5..jpg]

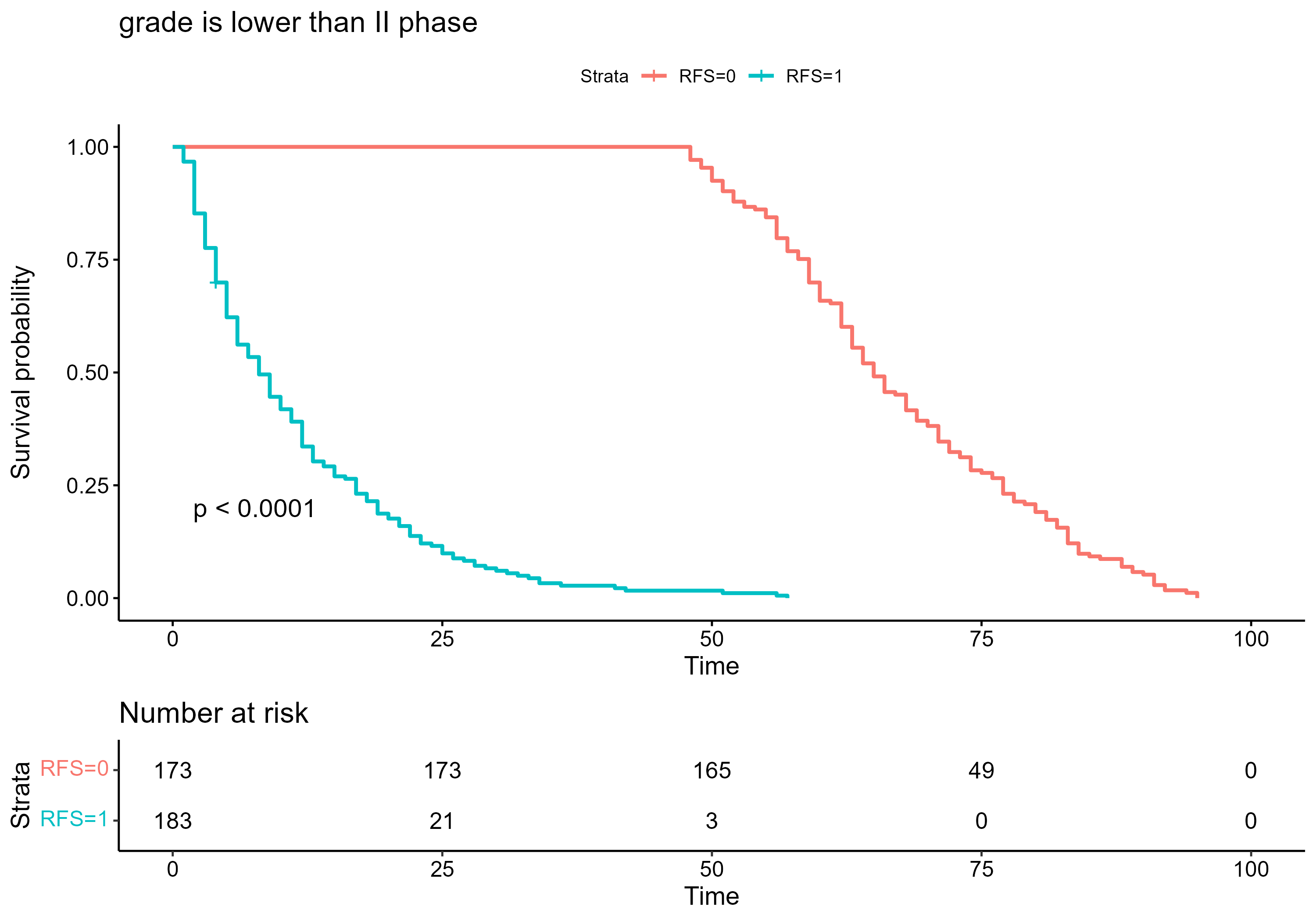

Supplement: Supplementary file 1 [file Data_Sheet_1.ZIP › Raw data/images/grade is lower than II phase.png]

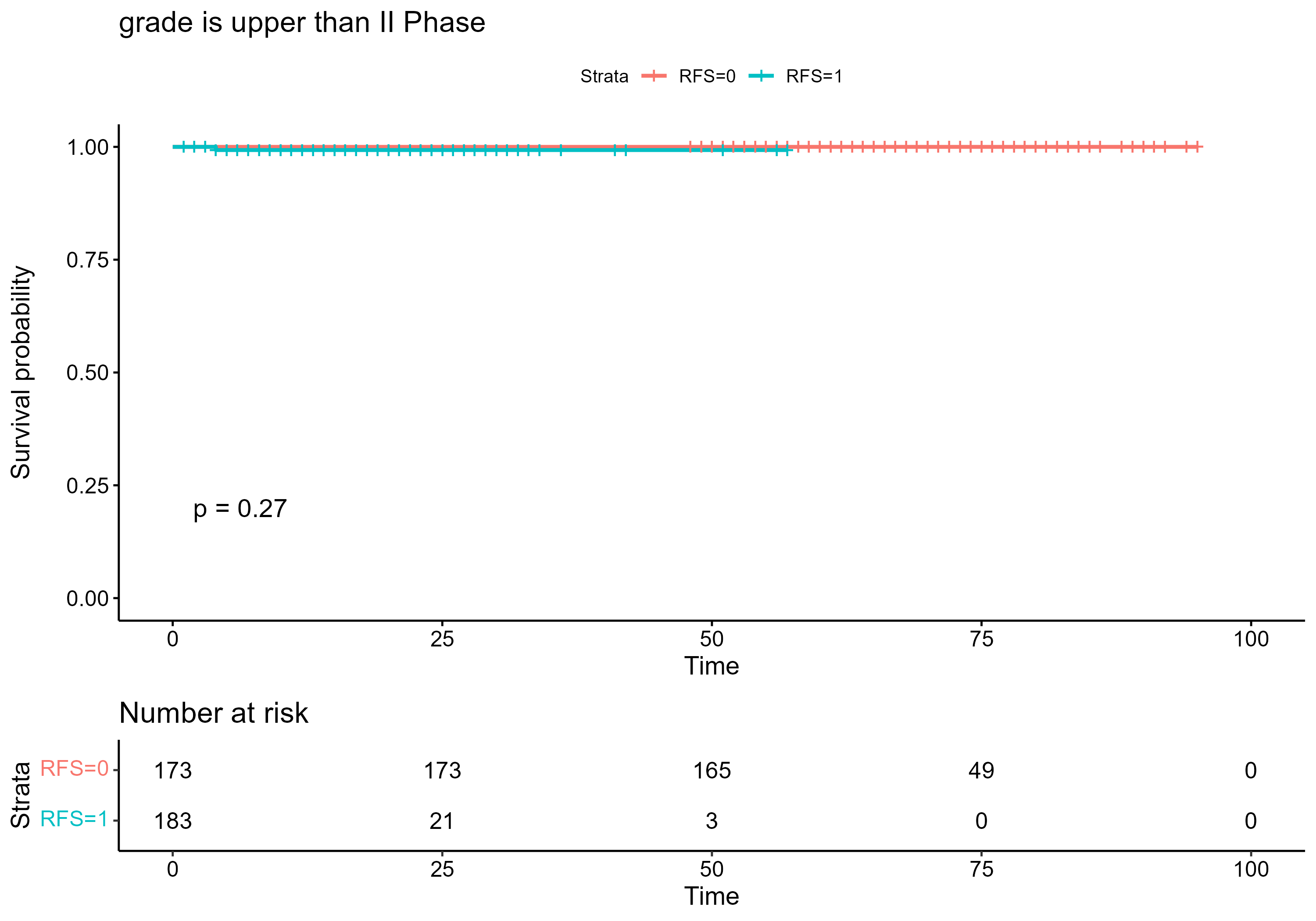

Supplement: Supplementary file 1 [file Data_Sheet_1.ZIP › Raw data/images/grade is upper than II Phase.png]

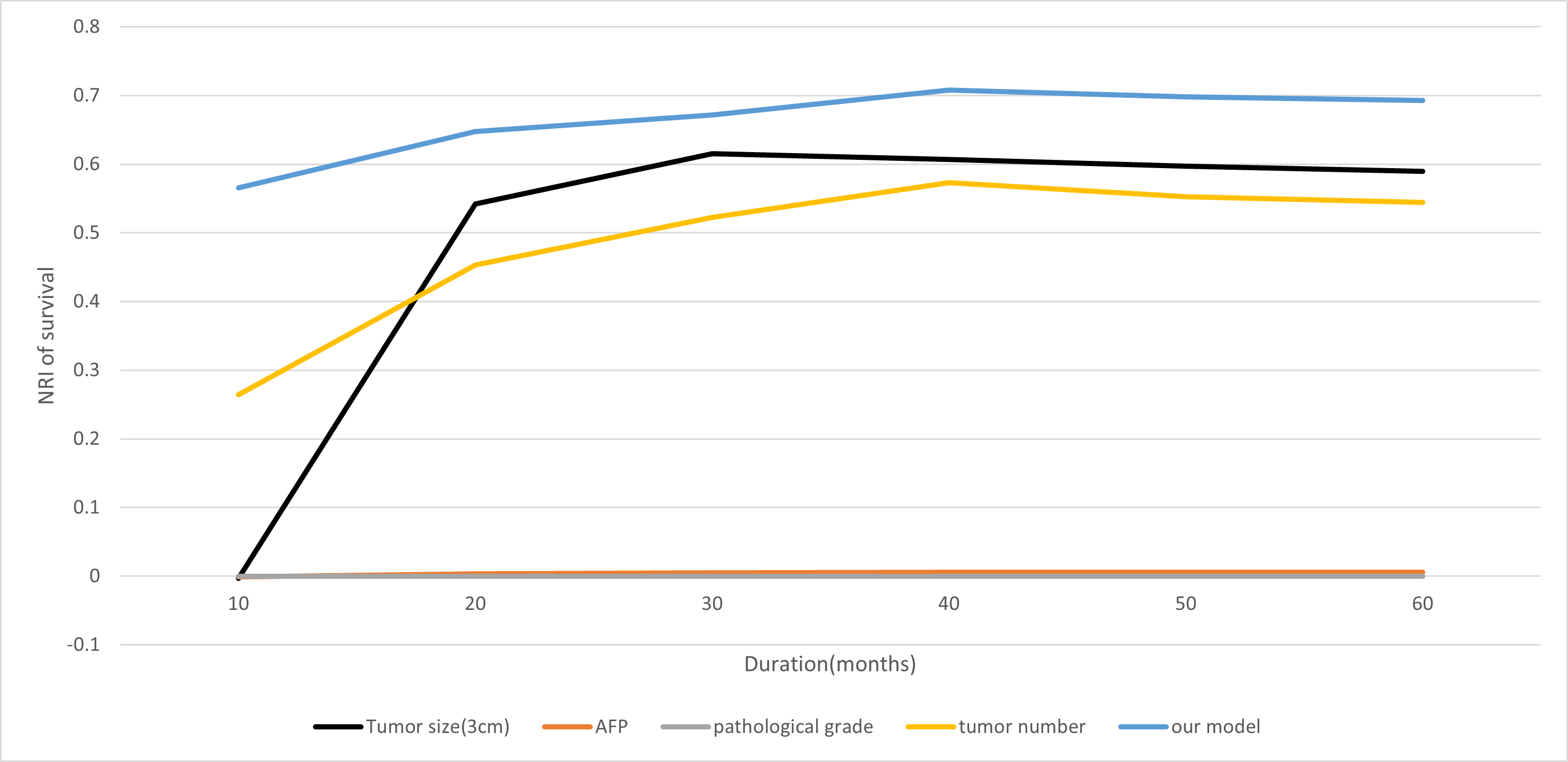

Supplement: Supplementary file 1 [file Data_Sheet_1.ZIP › Raw data/images/NRI.png]

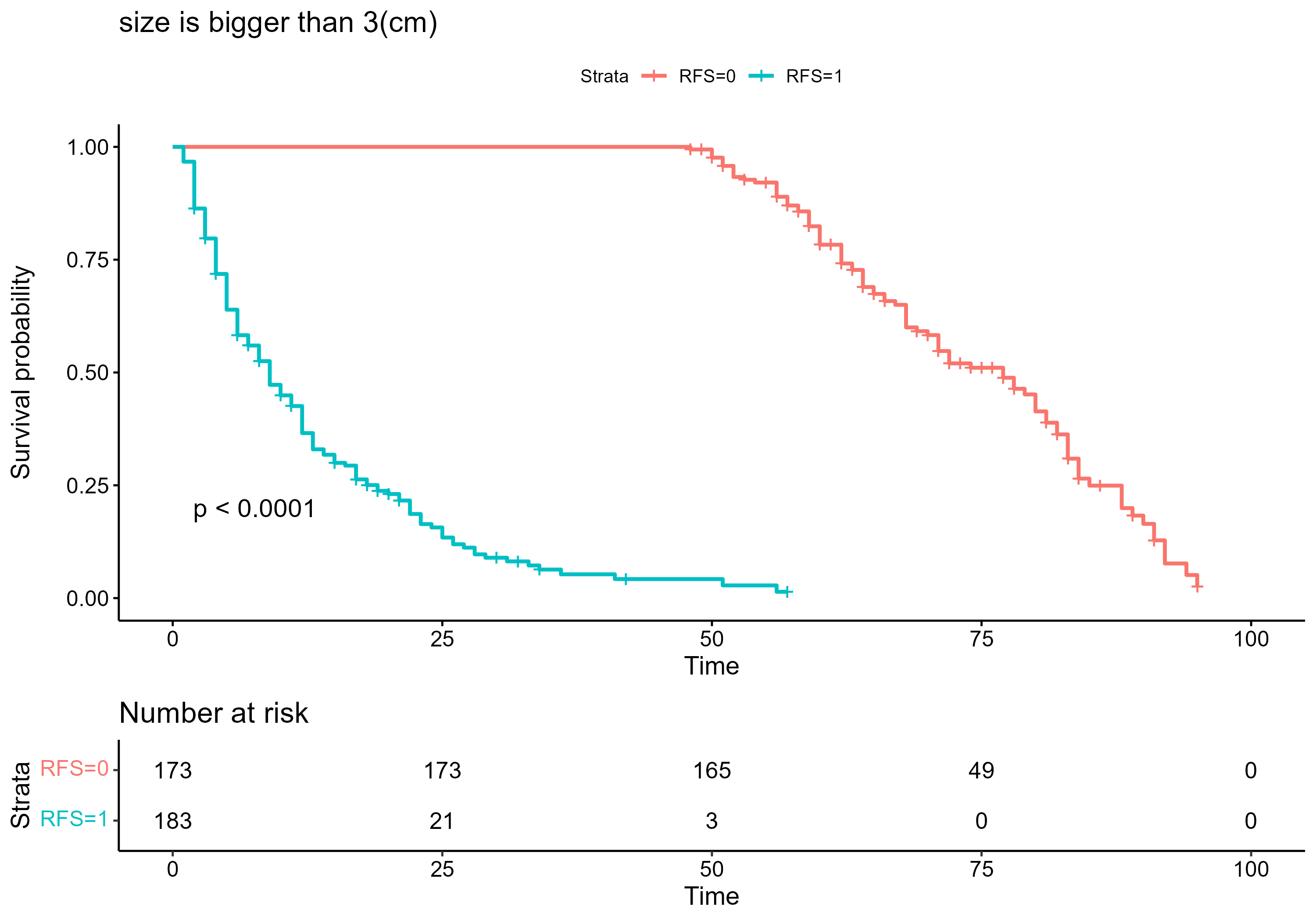

Supplement: Supplementary file 1 [file Data_Sheet_1.ZIP › Raw data/images/size is bigger than 3(cm).png]

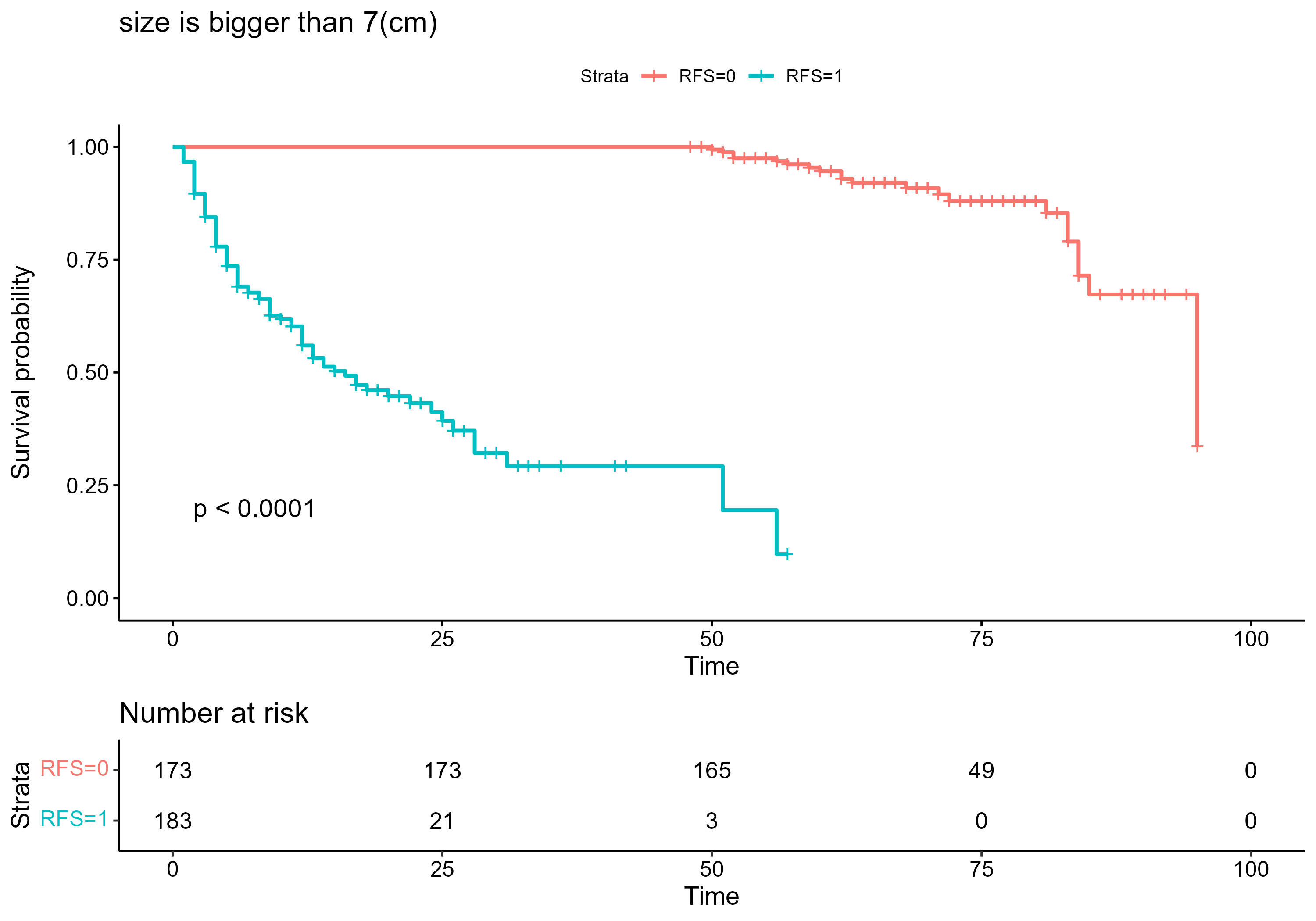

Supplement: Supplementary file 1 [file Data_Sheet_1.ZIP › Raw data/images/size is bigger than 7(cm).png]

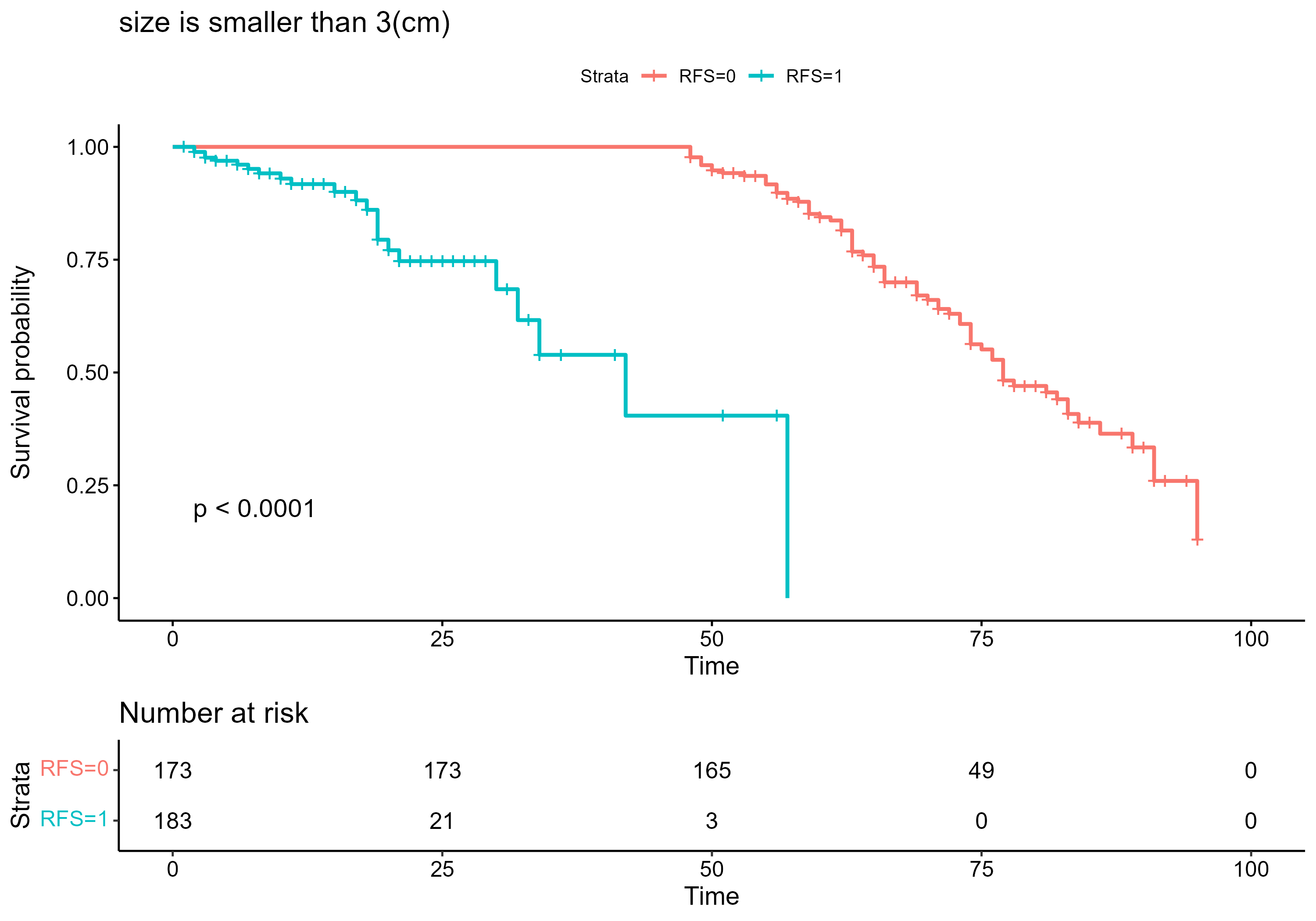

Supplement: Supplementary file 1 [file Data_Sheet_1.ZIP › Raw data/images/size is smaller than 3(cm).png]

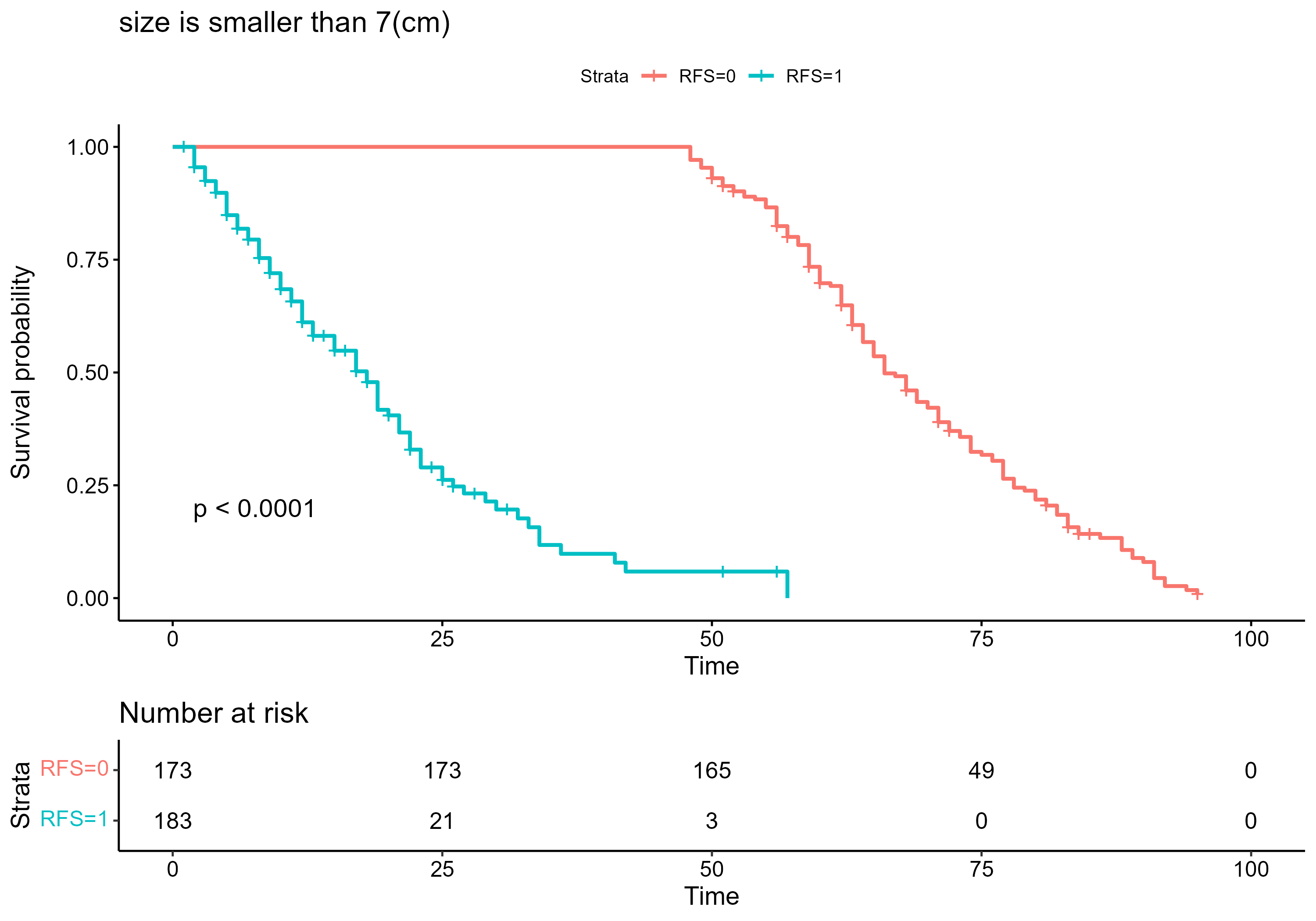

Supplement: Supplementary file 1 [file Data_Sheet_1.ZIP › Raw data/images/size is smaller than 7(cm).png]

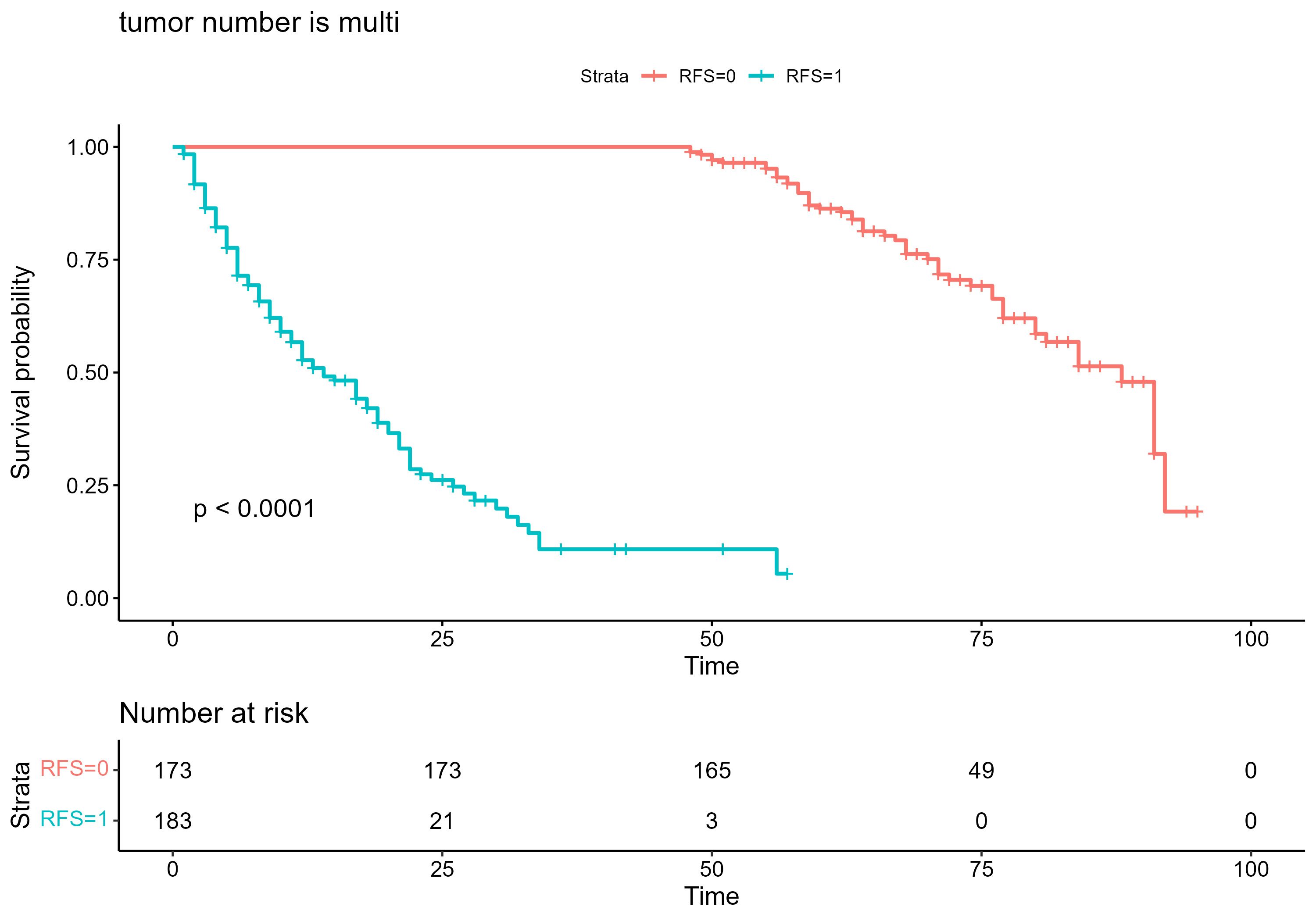

Supplement: Supplementary file 1 [file Data_Sheet_1.ZIP › Raw data/images/tumor number is multi.png]

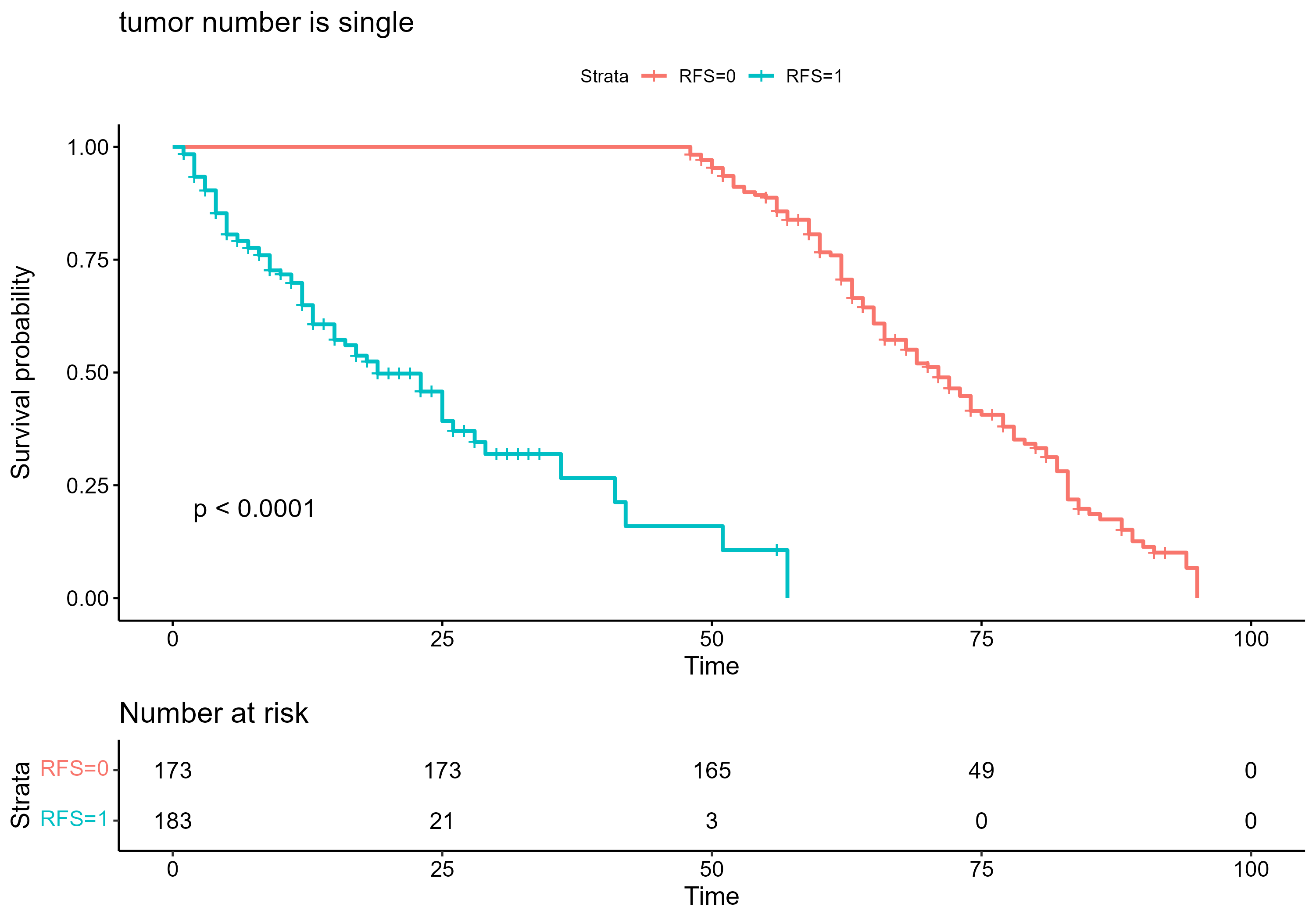

Supplement: Supplementary file 1 [file Data_Sheet_1.ZIP › Raw data/images/tumor number is single.png]

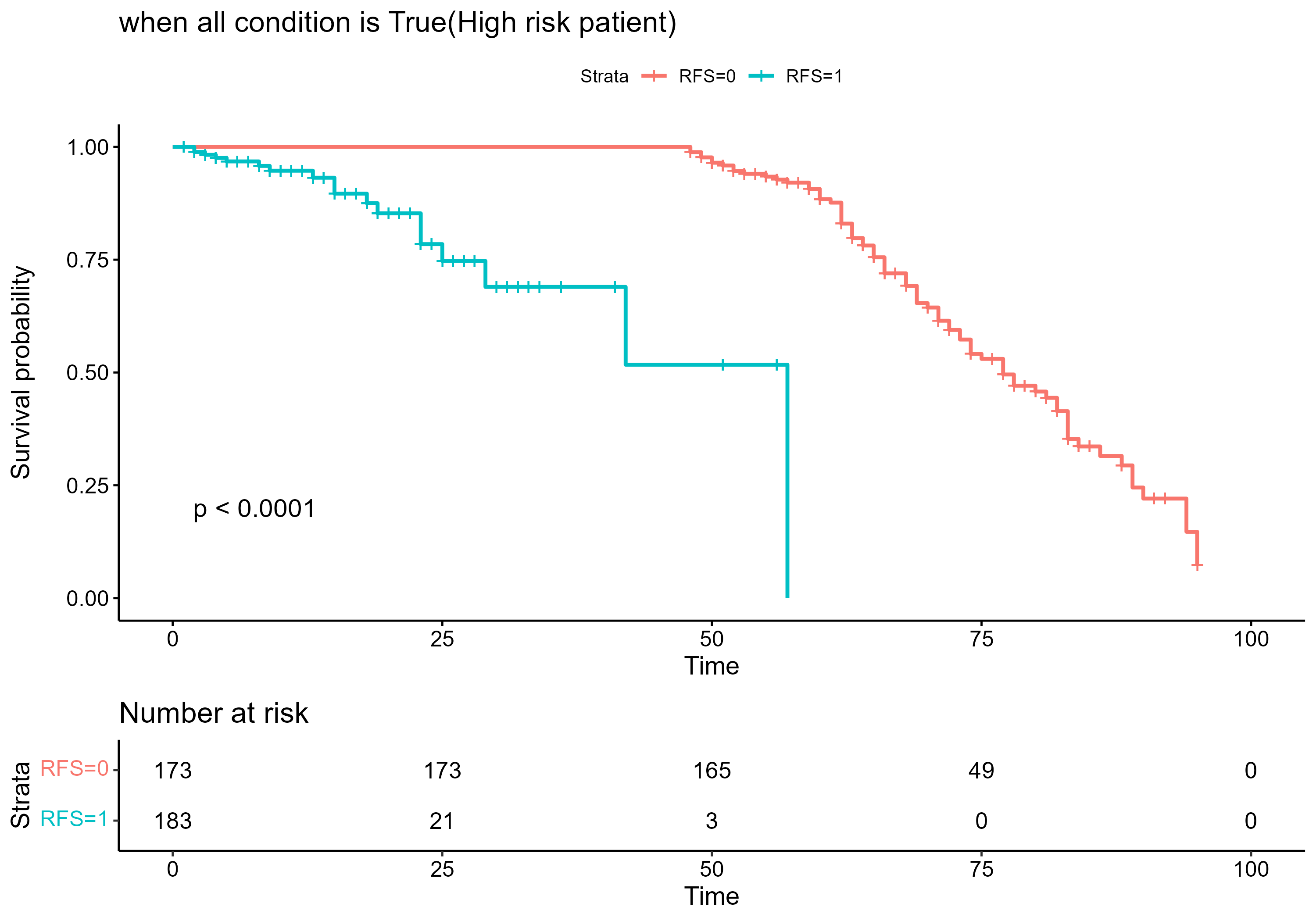

Supplement: Supplementary file 1 [file Data_Sheet_1.ZIP › Raw data/images/when all condition is True(High risk patient).png]

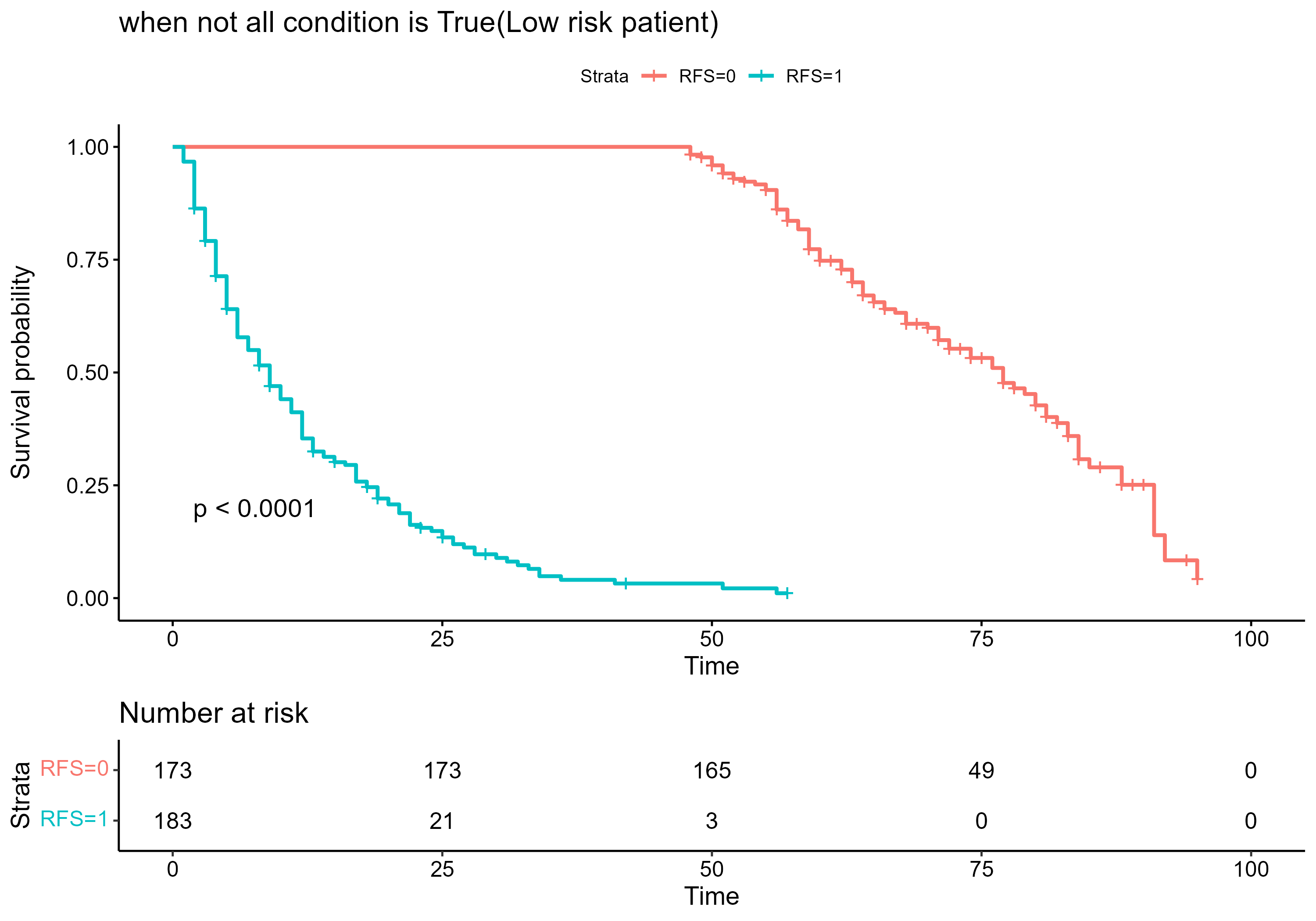

Supplement: Supplementary file 1 [file Data_Sheet_1.ZIP › Raw data/images/when not all condition is True(Low risk patient).png]

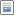

Supplement: Supplementary file 1 [file Data_Sheet_1.ZIP › Raw data/source data and codes/codes/tabnet/docs/_static/file.png]

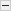

Supplement: Supplementary file 1 [file Data_Sheet_1.ZIP › Raw data/source data and codes/codes/tabnet/docs/_static/minus.png]

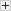

Supplement: Supplementary file 1 [file Data_Sheet_1.ZIP › Raw data/source data and codes/codes/tabnet/docs/_static/plus.png]
